# Improving gene isoform quantification with miniQuant

**DOI:** 10.1038/s41587-025-02633-9

**Published:** 2025-06-03

**Authors:** Haoran Li, Dingjie Wang, Qi Gao, Puwen Tan, Yunhao Wang, Xiaoyu Cai, Aifu Li, Yue Zhao, Andrew L. Thurman, Seyed Amir Malekpour, Ying Zhang, Roberta Sala, Andrea Cipriano, Chia-Lin Wei, Vittorio Sebastiano, Chi Song, Nancy R. Zhang, Kin Fai Au

**Affiliations:** 1 Gilbert S. Omenn Department of Computational Medicine and Bioinformatics, University of Michigan, Ann Arbor, MI USA; 2 Department of Biomedical Informatics, The Ohio State University, Columbus, OH USA; 3 Department of Internal Medicine, University of Iowa, Iowa City, IA USA; 4 Department of Obstetrics and Gynecology, Stanford University, Stanford, CA USA; 5 Institute for Stem Cell Biology and Regenerative Medicine, Stanford University, Stanford, CA USA; 6 The Jackson Laboratory for Genomic Medicine, Farmington, CT USA; 7 Division of Biostatistics, College of Public Health, The Ohio State University, Columbus, OH USA; 8 Department of Statistics and Data Science, The Wharton School, University of Pennsylvania, Philadelphia, PA USA; 9 Present Address: Gilbert S. Omenn Department of Computational Medicine and Bioinformatics, University of Michigan, Ann Arbor, MI USA

**Keywords:** Transcriptomics, RNA sequencing, Computational models, Statistical methods, Machine learning

## Abstract

RNA sequencing has been widely applied for gene isoform quantification, but limitations exist in quantifying isoforms of complex genes accurately, especially for short reads. Here we identify genes that are difficult to quantify accurately with short reads and illustrate the information benefit of using long reads to quantify these regions. We present miniQuant, which ranks genes with quantification errors caused by the ambiguity of read alignments and integrates the complementary strengths of long reads and short reads with optimal combination in a gene- and data-specific manner to achieve more accurate quantification. These results are supported by rigorous mathematical proofs, validated with a wide range of simulation data, experimental validations and more than 17,000 public datasets from GTEx, TCGA and ENCODE consortia. We demonstrate miniQuant can uncover isoform switches during the differentiation of human embryonic stem cells to pharyngeal endoderm and primordial germ cell-like cells.

## Abstract (web summary)

Gene isoforms are quantified with a machine learning method that optimally integrates long and short sequencing reads.

## Main

Gene isoforms, arising mainly from the alternative splicing of many genes (for example, in human^1^), enhance the diversity of RNAs, proteins and phenotypic traits^2^. Numerous studies of alternative splicing have shown critical roles in disease mechanisms^3^ and targeted therapies^4^. However, gene isoforms have not been studied as extensively as alternative splicing mechanisms because critical unresolved questions persist in gene isoform profiling.

**Fig. 1:**
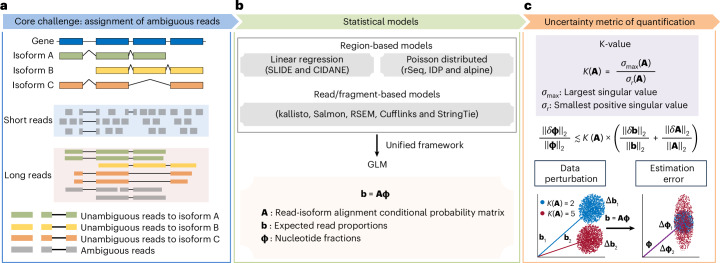
K-value indexes the uncertainty in gene isoform quantification. **a**, Schematic illustration of gene isoform structure and corresponding short and long reads assignment. Shared exonic sequences among isoforms lead to uncertainty in assigning reads to gene isoforms of origin, especially for short reads due to the limited read lengths. **b**, Overview of the statistical models used in gene isoform abundance estimation. Methods have been developed to estimate gene isoform abundance using region-based or read-/fragment-based models. Both models can be unified into a GLM framework **b** = **Aϕ**. **c**, Top, design of a new statistic, K-value, to index the gene isoform quantification error in RNA-seq data. Middle, for full-column-rank matrix **A**, when ${\Vert \delta {{ \textbf{A}}}\Vert }_{2} \ll {\Vert {{\textbf{A}}}\Vert }_{2}$, *K*(**A**) is low, and the error **b** − **Aϕ** is small, the relative quantification error $\frac{{\Vert \delta {\mathbf{\upphi}}\Vert}_{2}}{{\Vert{\mathbf{\upphi}}\Vert}_{2}}$ is bounded approximately by $K({\textbf{A}}) \times(\frac{{\Vert \delta {\textbf{b}}\Vert }_{2}}{{\Vert {\textbf{b}}\Vert }_{2}}+\frac{{\Vert \delta {{\textbf{A}}}\Vert }_{2}}{{\Vert {{\textbf{A}}}\Vert }_{2}})$ (Supplementary Note 3). Bottom, higher K-value indicates the model is more sensitive to perturbations, thus the quantification results are less reliable. Fix the value of **ϕ**; given same level of perturbations on **b**, the linear model **b** = **Aϕ** tends to have larger estimation errors *δ***ϕ** when the K-value of **A** is larger.

Unresolved questions pertain primarily to the read alignment uncertainty to gene isoform of origin. Since isoforms of a gene share exonic sequences, many short reads cannot be unambiguously assigned to their gene isoforms of origin^5,6^ (Fig. 1a). Although long-read RNA sequencing (RNA-seq) (for example, by the techniques of Pacific Biosciences (PacBio)^7^ and Oxford Nanopore Technologies (ONT)^8^) has been demonstrated to be qualitatively superior for identifying gene isoforms in many biomedical contexts^9^, there still exists a critical proportion of fragmental long reads that are probably assigned ambiguously to the gene isoform of origin, even for short isoforms^10,11^ (Supplementary Fig. 1). Most methods for estimating gene isoform abundance establish hierarchical models, where the gene isoform abundances are the unknown parameters controlling the emission probabilities of the observed reads. Gene isoform abundances are then estimated by maximizing the likelihood (Fig. 1b). Thus, gene isoform quantification can be viewed as a data deconvolution process incorporating information from both transcriptome (for example, exon–isoform structure) and data (for example, read length and alignment positions)^12^. Since these features of genes and data determine the difficulty of data deconvolution, the quantification errors can vary substantially across data and genes with different exon–isoform structures, that is, some genes can be quantified accurately at the isoform level, while others cannot. Therefore, we build a framework to determine whether a gene isoform can be quantified accurately and, thus, can be studied reliably.

Although longer read length of long-read RNA-seq data has been qualitatively shown to improve gene isoform quantification^13,14^, this has not been assessed rigorously. Since the coverage of current long-read sequencing data is still much lower than that of short-read sequencing data, it remains unclear to what extent, and for which genes, the benefits of longer read length offset the cost of reduced library size. It is not known how long reads and short reads should be integrated to harness the strengths of both technologies, or what the optimal design of hybrid sequencing experiments using both long reads and short reads should be.

Here, we propose the generalized condition number (referred to as ‘K-value’) as a gene- and data-specific proxy for gene isoform quantification error caused by data deconvolution. Some previous attempts of evaluating gene isoform quantification uncertainty simply adopted the number of isoforms to define gene isoform complexity^15–17^, which lacks a rigorous foundation in data science. Some other studies calculated the postquantification metrics, such as bootstrap-based computation of confidence interval^18,19^, information matrix^20,21^ and overdispersion^22,23^. Compared with the previous studies, K-value is a rigorous measurement of gene isoform complexity regarding quantification difficulty given read length, so it can be calculated before data collection and analysis and thus is useful to improve the design of data collection, such as the selection of short-read versus long-read sequencing. Using the K-value, we can sort and filter genes with regard to the reliability of their gene isoform quantifications (Fig. 1c). We show that, across different paradigms of RNA-seq quantification, K-value is a general and reliable proxy for quantification error. This is shown through mathematical derivation, simulation data, experimental validation and applications to vast amount of consortium-scale data (>17,000 datasets from GTEx^24,25^, TCGA^25,26^ and ENCODE^27^).

Next, we present the software miniQuant^28^ (https://github.com/Augroup/miniQuant) that harnesses long reads to improve gene isoform quantification especially for complex exon–isoform structures with high K-values. We also dissect the weakness of long-read-based quantification on the lowly-expressed gene isoforms because of the low coverage of relatively low-throughput long-read RNA-seq. To achieve optimal integration of long reads and short reads, miniQuant adopts a machine learning approach to adaptively find the gene- and data-specific weighting scheme between two data types. In benchmarks, miniQuant improves upon naive integration of long reads and short reads with uniform weight, or existing methods that rely on short reads alone or long reads alone.

Benefiting from the accurate quantification by miniQuant, we further characterize the switching of gene isoforms during the differentiation of human embryonic stem cells (ESC) to pharyngeal endoderm (PE) and primordial germ cell-like cells (PGC).

## Results

### Index gene isoform quantification error by K-value

**Fig. 2:**
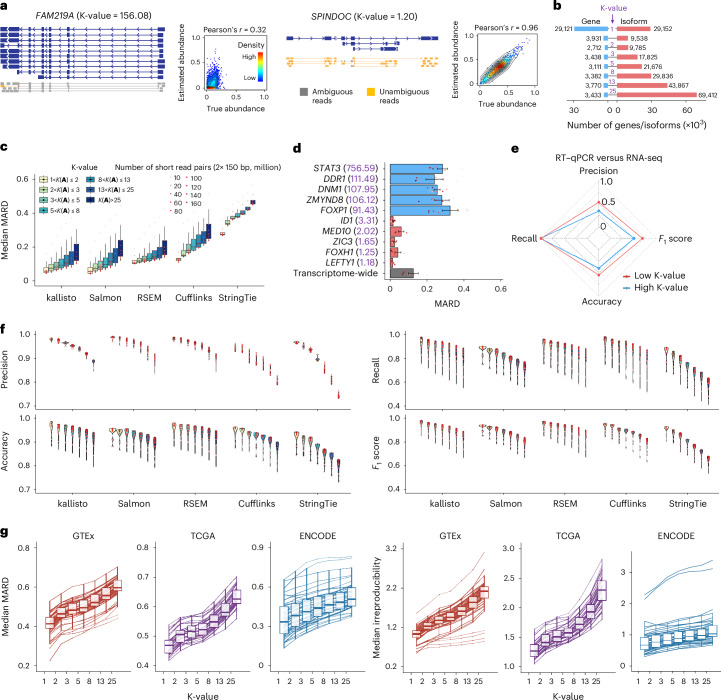
K-value is an indicator of gene isoform quantification error. **a**, Schematic illustration of the gene isoform structures and their corresponding aligned short reads of *FAM219A* (left) and *SPINDOC* (right). The two-dimension density plot (*n* = 200 simulations) represents the correlations between true and estimated abundance. **b**, Barplot representing the number of genes and corresponding isoforms within each K-value group. The first K-value group includes genes with K-value = 1. All subsequent K-value groups are genes with K-values between the two numbers above and below the line. The number of genes and isoforms are labeled on the left and right next to the bars, respectively. **c**, Boxplot showing the median MARD of genes within each K-value group across sequencing depths (*n* = 9) quantified using five different tools. Only genes with expression levels TPM > 1 are retained for visualization. In boxplots, the hinges represent the first and third quartiles, the center line represents the median, and the whiskers extend to the smallest and largest datapoints within 1.5 interquartile from the hinges. All boxplots in the subsequence analysis have the same definition unless specified. **d**, Barplot representing the MARD of ten indicated genes with low (red) and high (blue) K-values across sequencing depths (*n* = 9) quantified by kallisto. The overall MARD (gray) are calculated based on the median MARD of all genes with expression level TPM > 1. K-values are labeled in brackets along with the gene symbol. In barplots, data are presented as mean values ± s.e. All barplots in the subsequence analysis have the same definition unless specified. **e**, Comparison of the performance (precision, recall, accuracy and *F*_1_ score) between differentially expressed genes isoforms with low (red) and high (blue) K-values. **f**, Violin plot represents the performance (precision, recall, accuracy and *F*_1_ score) of identifying differentially expressed gene isoforms between ESC and DE across sequencing depths (*n* = 9) within each K-value group quantified using five different tools. **g**, Boxplot representing the median MARD (left) and irreproducibility (right) of genes within each K-value group per sample from GTEx (*n* = 54 human tissues), TCGA (*n* = 32 cancer types) and ENCODE (*n* = 46 cell lines and human tissues). Only genes with expression levels TPM > 1 are retained for visualization.

The deconvolution of ambiguous read–isoform alignment for gene isoform quantification can be represented in a form of a generalized linear model (GLM) $${\textbf{b}}={\textbf{A}}{\mathbf{\upphi}},$$ where $${\mathbf{\upphi} }={\left({\phi }_{1},\cdots ,{\phi }_{T}\right)}^{{\prime} }$$ and $${\mathbf{\uptheta}} ={\left(\right.\!{\theta }_{1},{\theta }_{2},\cdots ,}$$$${\left.{\theta }_{T}\right)}^{{\prime}}$$ denote the nucleotide fractions^29^ and the relative abundance of *T* gene isoforms, respectively, with $${\phi }_{t}=\frac{{\theta }_{t}{\widetilde{l}}_{t}}{\mathop{\sum }\nolimits_{r=1}^{T}{\theta }_{r}{\widetilde{l}}_{r}}$$ and $${\widetilde{l}}_{t}$$ being the effective length of gene isoform *t*; $${\textbf{b}}={\left({b}_{1},{b}_{2},\cdots ,{b}_{S}\right)}^{{\prime} }$$ denotes the read proportions for *S* exonic regions; $${\textbf{A}}=\left({A}_{{{st}}}\right)\in {{\mathbb{R}}}^{S\times T}$$ denotes the read-isoform alignments as a conditional probability matrix that incorporates exon–isoform structure and sequencing information (Fig. 1b and Supplementary Notes 1 and 2; Methods ‘K-value (generalized condition number)’). Thus, the estimation accuracy of **ϕ** and **θ** depends on **A**. For example, with short reads, because there are very few unique read-isoform alignments, the isoform quantification of gene *FAM219A* deviates by a large amount from the true values; in contrast, the estimates and true values are close for the *SPINDOC* isoforms since all have unique read alignments (Fig. 2a). As **A** represents the read–isoform alignments, we establish the statistic $$K\left({\textbf{A}}\right)=\frac{{\sigma }_{\max }}{{\sigma }_{r}}$$ (referred to as ‘K-value’) to measure the influence of ambiguous read alignment in gene isoform quantification, where *σ*_max_ and *σ*_r_ are the largest and smallest positive singular values of **A** (Fig. 1c; Methods ‘K-value (generalized condition number)’).

In mathematics, we demonstrate that the relative quantification error $\frac{{\|\delta {\mathbf{\upphi}} \|}_{2}}{{\|{\mathbf{\upphi} }\|}_{2}}$ is bounded by $K({\textbf{A}})\frac{{\|\delta {\textbf{b}}\|}_{2}}{{\|{\textbf{b}}\|}_{2}}$ and approximately bounded by $K({\textbf{A}})\frac{{\|\delta {\textbf{A}}\|}_{2}}{{\|{\textbf{A}}\|}_{2}}$ (Fig. 1c and Supplementary Note 3), where $\delta {\mathbf{\upphi}}$ denotes the absolute quantification error, $\delta {\textbf{b}}$ denotes the variation of sequencing coverage and $\delta {\textbf{A}}$ denotes perturbation of read-isoform alignments that arises primarily from false gene isoform identification. That is, K-value is an amplification factor of the input data variance to quantification error, and therefore genes with high K-values are prone to be quantified erroneously.

This association between K-value and quantification error can be validated and illustrated by the simulation data at different sequencing depths across five of the most commonly used gene isoform quantification tools^18,19,29–31^ (Methods ‘Existing methods,’ ‘Simulation data’ and ‘Performance evaluation’). To focus on the quantification error derived by data deconvolution (hereafter referred to as ‘deconvolution error’), we consider the genes with transcripts per million (TPM) > 1 in this section to avoid the dominating sampling error caused by the extremely small number of reads generated from low-expressed gene isoforms. We adopt the evaluation metric mean absolute relative difference^19^ (MARD) due to its superior robustness over relative error—with a range from 0 to 1, MARD effectively mitigates the impact of outliers while it preserves similar results to relative error (Supplementary Fig. 2; Methods ‘Simulation data’ and ‘Performance evaluation’).

**Extended Data Fig. 1:**
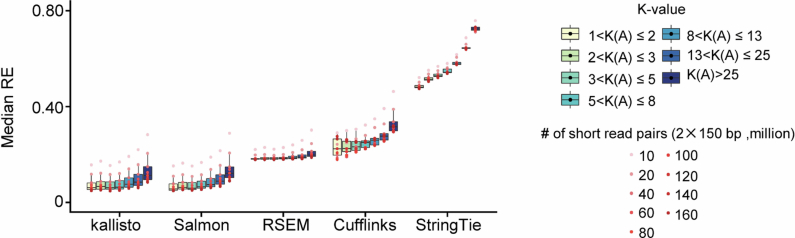
The association between relative error by short read-based tools and K-value. Boxplot shows the median RE of genes within each K-value group across sequencing depths (n = 9) quantified using five different short read-based tools. Only genes with expression levels TPM > 1 ware retained for visualization. RE: relative error. In box plots, the hinges represent the first and third quartiles, the center line represents the median, and the whiskers extend to the smallest and largest data points within 1.5 interquartile from the hinges. All box plots in the subsequence analysis have the same definition unless specified.

With 40 million short-read pairs, the median MARD of kallisto^18^ across genes is 0.0778 for the 9,538 GENCODE-annotated isoforms of 3,931 genes with 1 < *K*(**A**) ≤ 2 (Fig. 2b,c; Methods ‘Performance evaluation’). However, the median MARD increases almost threefold to 0.2174 for the 69,412 isoforms of 3,433 genes with *K*(**A**) > 25, and remains at a high level (0.1016) even when the number of short-read pairs increases to 160 million. In particular, these problematic genes include many crucial regulators associated with human ESC pluripotency or differentiation, such as *STAT3* (ref. ^32^), *FOXP1* (ref. ^33^), *DNM1*, *DDR1* and *ZMYND8* (refs. ^34–37^) (*K*(**A**) ≥ 90, average MARD ≥ 0.24 across nine sequencing depths from 10 million to 160 million short-read pairs). Quantification errors of these regulators are much higher than both the transcriptome-wide level (average median MARD 0.1259; Methods ‘Performance evaluation’) and the other regulators with low K-values, such as *FOXH1* (ref. ^38^), *MED10*, *LEFTY1*, *ID1* and *ZIC3* (refs. ^34–37^) (*K*(**A**) < 4, average MARD < 0.07; Fig. 2d). The strong association of quantification error with respect to K-value extends consistently from kallisto to the other widely used quantification software Salmon^19^, RSEM^29^, Cufflinks^31^ and StringTie^30^ (Fig. 2c and Extended Data Fig. 1).

Of note, K-value indexes the essential error caused by data deconvolution and it does not measure all possible components of quantification error, and thus the remarkable variation among tools represents their software specifics in taking care of different components of quantification errors.

**Extended Data Fig. 2:**
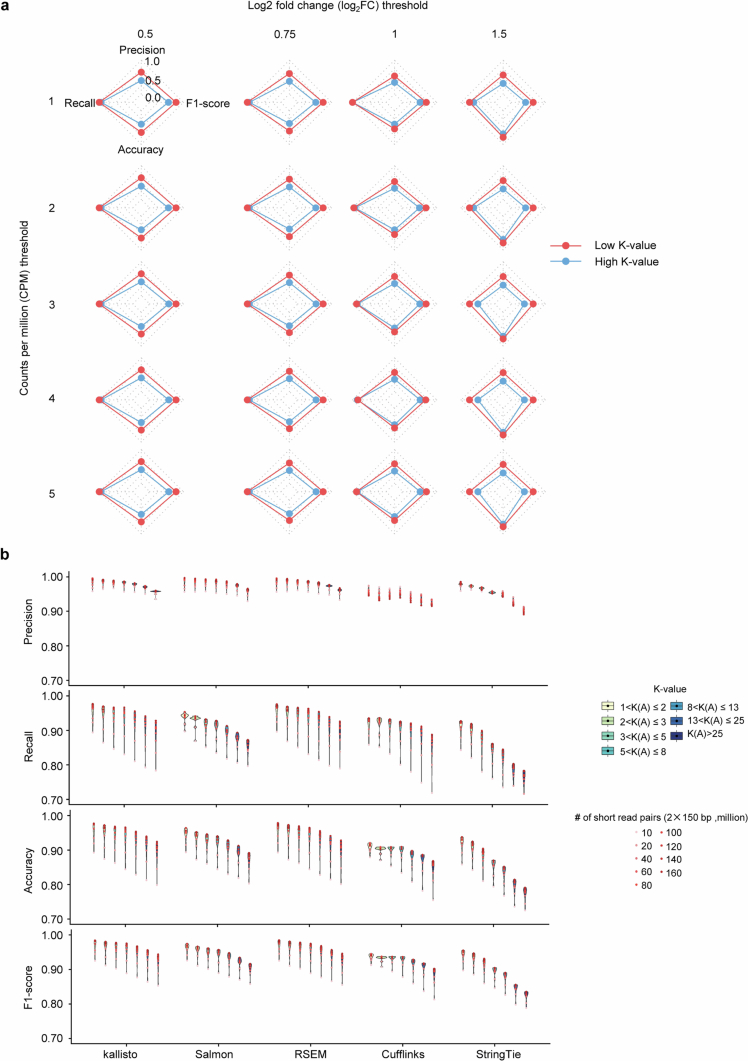
The association between the performance of differential gene isoform expression analysis and K-value. **a**, Comparison of the performance (precision, recall, accuracy, and F1-score) of differential gene isoform expression analysis between isoforms with low (red) and high (blue) K-values under different CPM and log_2_FC thresholds. The evaluation is based on 48 gene isoforms with low K-values (< 5) and 45 with high K-values (>25) measured with RT-qPCR in ESC and DE. Gene isoform expression levels in short-read sequencing data are quantified using kallisto. The significance of differentially expressed gene isoforms is assessed using the Student’s t-test for RT-qPCR data and edgeR for sequencing data under five expression (minimum CPM from 1 to 5 in at least two samples) and four (0.5, 0.75, 1, and 1.5) log_2_FC thresholds. **b**, The violin plot represents the performance (precision, recall, accuracy, and F1-score) of differential gene isoform expression analysis between GM12878 and K562 across sequencing depths (n = 9) within each K-value group quantified using five short read-based tools. The fold changes and significance of differential expression are assessed using the edgeR.

Hence, differential expression analysis for the gene isoforms with high K-values is also prone to error. As an experimental demonstration, the differential expression of 48 selected gene isoforms with low K-values and 45 with high K-values are calculated based on real short reads and quantitative PCR with reverse transcription (RT–qPCR), respectively, in the differentiation of human ESC (Methods ‘Real data,’ ‘Performance evaluation’ and ‘Experimental validation by RT–qPCR’): identification of truly differentially expressed gene isoforms in the group with low K-values outperforms that in the group with high K-values by higher precision, accuracy and *F*_1_ score (Fig. 2e). While the performance of identifying differentially expressed targets depends highly on the parametric thresholds (for example, log fold change and counts per million (CPM) in edgeR^39–41^), the low K-value group outperforms the high K-value one consistently regardless of the parametric changes (Extended Data Fig. 2a). This influence by K-value is further shown across the entire human transcriptome by four simulation datasets (Methods ‘Simulation data’ and ‘Performance evaluation’): the performance (that is, precision, recall, accuracy and *F*_1_ score) of identifying truly differentially expressed gene isoforms declines with increasing K-value, regardless of the choice of quantification tools (Fig. 2f and Extended Data Fig. 2b). For example, when gene isoforms are quantified by StringTie with 40 million short-read pairs, the precision, recall, accuracy and *F*_1_ score of differential expression analysis decrease by 0.2159, 0.2995, 0.1265 and 0.2700, separately, comparing the genes with low K-values (1–2) to those with high K-values (>25) (Fig. 2f). Therefore, sorting genes with K-values can guide the selection of reliable targets for rigorous downstream analysis at the gene isoform level.

**Extended Data Fig. 3:**
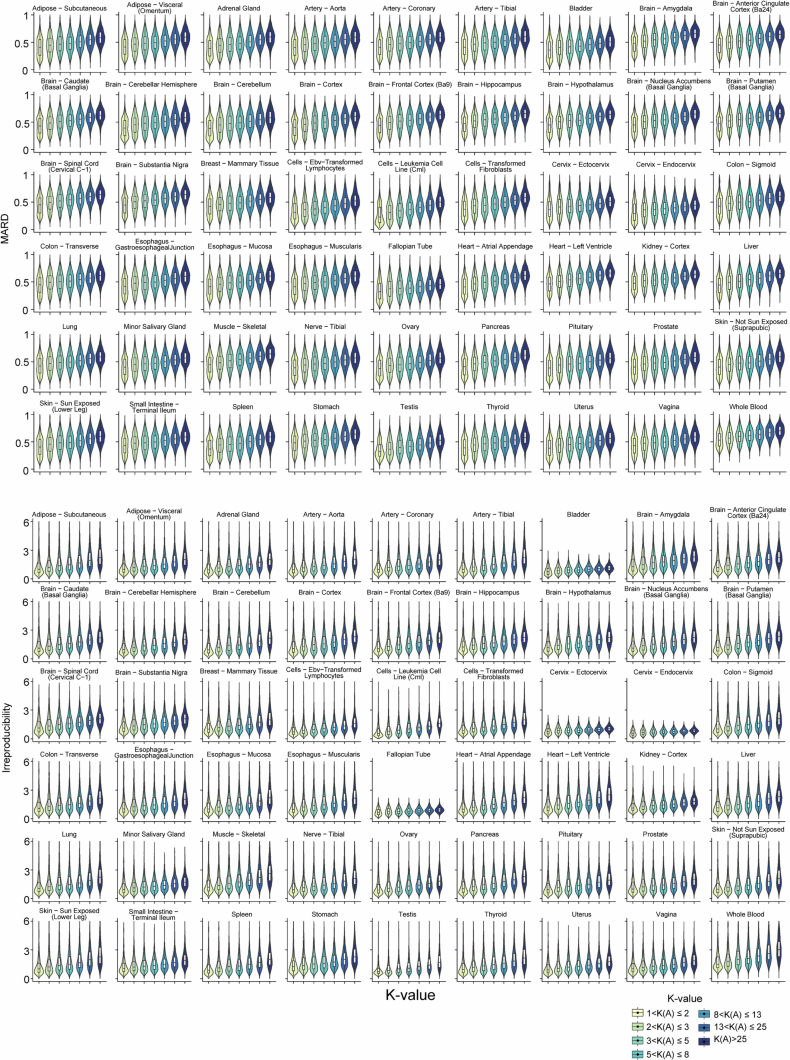
The association between the quantification error and K-value in the real data from GTEx consortium. The violin plot represents the MARD and irreproducibility within each K-value group in 54 GTEx human tissues. RSEM is used to quantify gene isoform abundance. The K-value here is estimated based on the annotation used in GTEx. Gene irreproducibility over 6 is removed before visualization. The n number of box plots are described in Supplementary Table 1.

**Extended Data Fig. 4:**
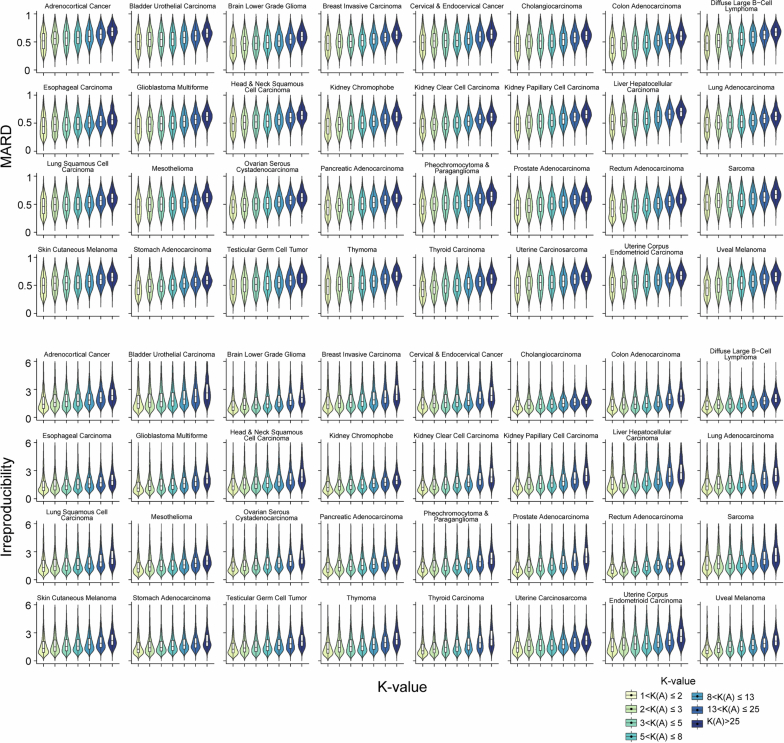
The association between the quantification error and K-value in the real data from TCGA consortium. The violin plot represents the MARD and irreproducibility within each K-value group in 32 TCGA cancer types. RSEM is used to quantify gene isoform abundance. The K-value here is estimated based on the annotation used in TCGA. Gene irreproducibility over 6 is removed before visualization. The n number of box plots are described in Supplementary Table 2.

**Extended Data Fig. 5:**
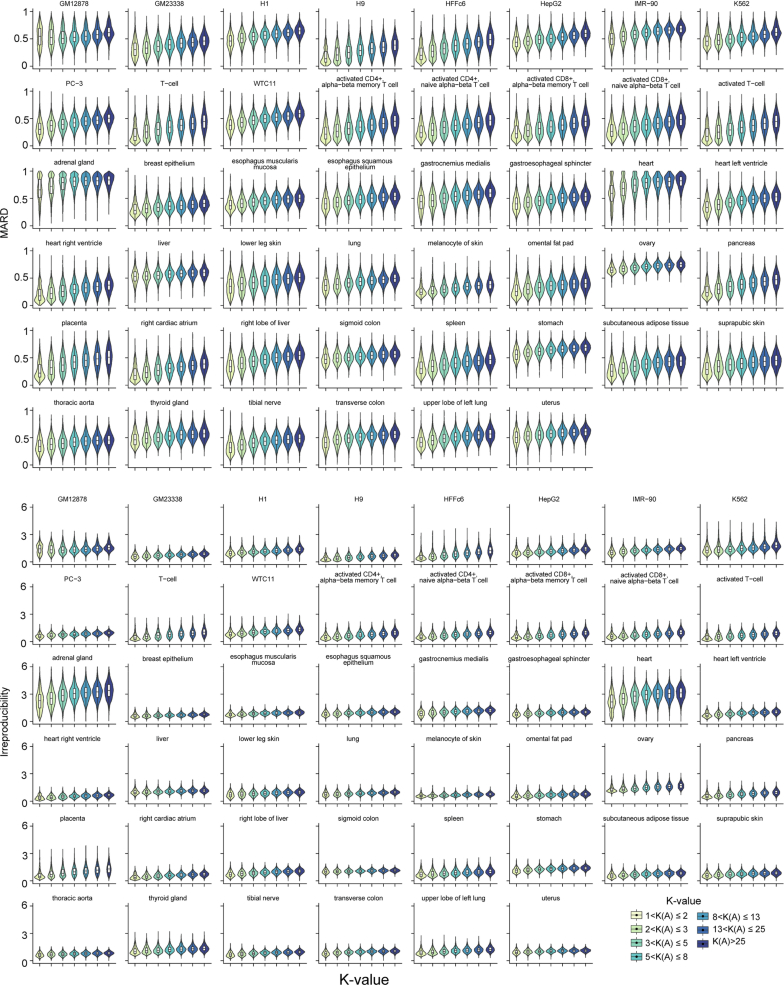
The association between the quantification error and K-value in the real data from ENCODE consortium. The violin plot represents the MARD and irreproducibility within each K-value group in 46 ENCODE human cell lines and tissues. The kallisto is used to quantify gene isoform abundance. Gene irreproducibility over 6 is removed before visualization. The n number of box plots are described in Supplementary Table 3.

Beyond simulation, K-value as a proxy for quantification error is also validated in the vast amount of real short-read data from three consortia: GTEx (54 human tissues; 7,862 datasets, read lengths from 76 to 106 bp), TCGA (32 cancer types; 9,185 datasets, read lengths from 48 to 100 bp) and ENCODE (46 cell lines and human tissues, 423 datasets, read lengths from 43 to 101 bp) (Supplementary Tables 1–3). Without groundtruth in the real data, the MARD and irreproducibility (that is, the coefficient of variation among replicates; Methods ‘Performance evaluation’) of gene isoform quantification are used to assess error and both increase with respect to K-value in almost all biological samples from three consortia (Fig. 2g and Extended Data Figs. 3–5). As K-values increase from 1 to >25, the consortium-wise median values of the transcriptome-wide median MARD increase by 0.1830, 0.1559 and 0.1721 in GTEx, TCGA and ENCODE data, separately; the corresponding overall median irreproducibility increases from 1.03 to 2.12, 1.27 to 2.30 and 0.67 to 1.03, separately.

Of note, the extent of these trends varies across samples (for example, cancer types, tissues and cell lines) and consortia. This variation is due to the critical differences in biological contexts (that is, different gene expressions), biological samples and resources (for example, various acquisition sources of cell lines), sequencing platforms, data quality, experimental sites and bioinformatics software used in these consortia, while K-value focuses on the influence of gene isoform complexity in quantification. Regardless of these differences in biological and technological settings, the positive associations of MARD and irreproducibility with respect to K-value hold in general. Therefore, K-value is a very robust index of the intrinsic quantification error in the inherent deconvolution procedure of RNA-seq data. Given gene isoform annotation and input data, miniQuant outputs K-value of each gene for users to interpret the reliability of the quantitative analysis at the gene isoform level.

### Long reads reduce deconvolution uncertainty

**Fig. 3:**
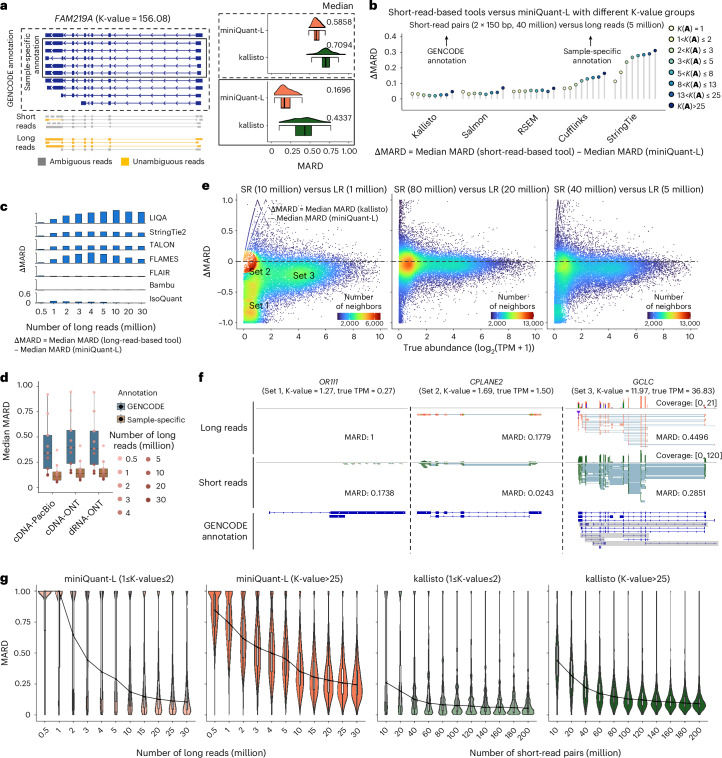
Long reads improve gene isoform quantification by reducing data deconvolution uncertainty. **a**, Left, schematic illustration of the gene isoform structure and corresponding aligned short reads of *FAM219A*. Right, raincloud plots represent the median MARD of *FAM219A* quantified by miniQuant-L and kallisto under GENCODE (top) and sample-specific (bottom) annotation (*n* = 200 simulations). **b**, Barcharts represent the ΔMARD between five short-read-based tools and miniQuant-L within eight different K-value groups under GENCODE and sample-specific annotation, respectively. **c**, Comparison of median MARD between seven long-read-based tools and miniQuant-L across nine different sequencing depths on cDNA-ONT data under GENCODE annotation. **d**, Comparison of median MARD among cDNA-PacBio, cDNA-ONT and dRNA-ONT protocols across sequencing depths (*n* = 9) under GENCODE and sample-specific annotations. **e**, Two-dimension scatterplot shows the ΔMARD between kallisto and miniQuant-L against gene expression levels on different short-read and long-read sequencing depth combinations. SR, short-read pairs. LR, long reads. **f**, Coverage tracks and gene isoform structures of three indicated genes, *OR1I1*, *CPLANE2* and *GCLC*, from Set 1, Set 2 and Set 3 in **e** (left), respectively. The K-value and TPM are labeled in the bracket under the gene symbols. *CPLANE2* is described in Supplementary Note 5. In *GCLC*, low-expressed (TPM < 1) gene isoforms are covered in gray. **g**, Violin plot shows the MARD of genes with low K-values (≤2) (*n* = 2,826 genes) and high K-values (>25) (*n* = 3,339 genes) by miniQuant-L and kallisto across 11 short-read and long-read sequencing depths. In box plots, the hinges represent the first and third quartiles, the center line represents the median, and the whiskers extend to the smallest and largest datapoints within 1.5 interquartile from the hinges.

Compared with short reads, long reads improve gene isoform quantification accuracy in two aspects: (1) less ambiguity of long-read alignment can reduce the uncertainty of data deconvolution. For example, given the GENCODE-annotated gene structure and 2 × 150 bp short-read pairs, the MARD of the gene *FAM219A* with extremely high K-value (156.08) is as large as 0.7094 by kallisto, while long reads reduce MARD to 0.5858 by the long-read-alone mode of miniQuant (referred to as ‘miniQuant-L’) (Fig. 3a; Methods ‘Community-wise gene isoform abundance estimation model’ and ‘Simulation data’). In parallel, (2) long reads can generate a more accurate sample-specific annotation. To avoid the bias caused by using a specific gene isoform identification tool, we mimic the sample-specific annotation by choosing the gene isoforms with TPM ≥ 1 from a human transcriptome profile obtained from the GENCODE annotation and a real dataset from H1-hESC cell line (Methods ‘Reference genome and gene annotations’). The MARD of *FAM219A* is as low as 0.1696 when long-read coverage and long-read-based sample-specific annotation are used. Extending the evaluation to the entire transcriptome, the reduction of the median MARD by miniQuant-L with the long-read-based sample-specific annotation over five short-read-based tools with the GENCODE annotation is 0.0138–0.2257, even when long reads are far less than short-read pairs (5 million versus 40 million) (Fig. 3b). Moreover, the reduction of the median MARD by miniQuant-L is associated with the K-value, that is, long reads make more remarkable quantification improvement (that is, ΔMARD) when the targets are of very high K-values and thus challenging in the short-read-based quantification (Fig. 3b). For example, the quantification improvement by miniQuant-L over StringTie is 0.3117 and 0.1720 for the genes with the K-values > 25 and from 1 to 2, respectively.

**Extended Data Fig. 6:**
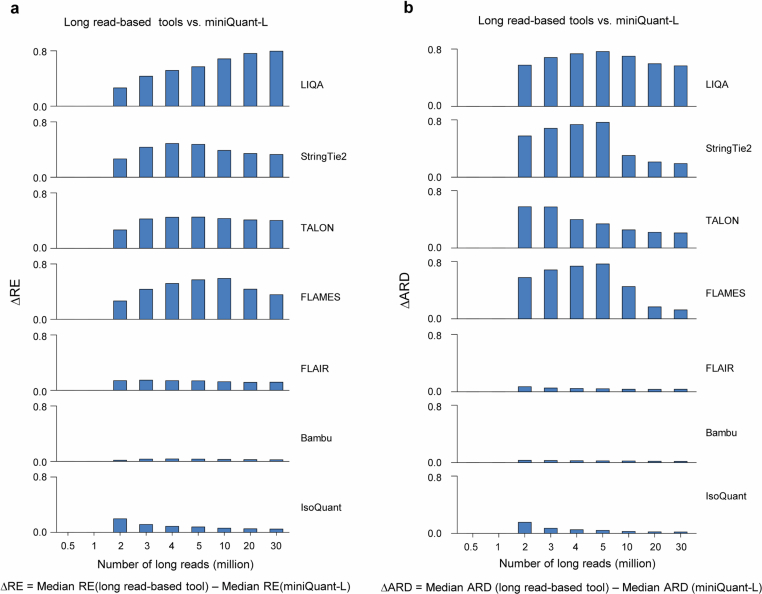
Comparison of the relative error and absolute relative difference among long-read based tools. **a**, Comparison of median RE estimated by miniQuant-L and seven long read-based tools across nine different sequencing depths on cDNA-ONT data under GENCODE annotation. Median REs shown here are from 0 to 1. RE: relative error. **b**, Comparison of median ARD at gene isoform level estimated by miniQuant-L and seven long read-based tools across nine different sequencing depths on cDNA-ONT data under GENCODE annotation. ARD: absolute relative difference.

MiniQuant-L uses the joint likelihood function that includes comprehensive data features so that the unique information of long reads can be fully taken into account (Supplementary Note 4). MiniQuant-L outperforms seven existing long-read-based gene isoform quantification tools^42–48^ (Fig. 3c). Especially, the average ΔMARD across nine sequencing depths (0.5–30 million) of miniQuant-L over TALON^47^, StringTie2 (ref. ^48^), FLAMES^44^ and LIQA^46^ is as high as 0.2130–0.5084, and the ΔMARD becomes more striking when >1 million long reads are used. Compared with Bambu^43^, FLAIR^45^ and IsoQuant^42^, miniQuant-L also performs better with the average ΔMARD of 0.0140, 0.0433 and 0.0776, respectively, and the difference of their performance varies mildly across nine sequencing depths (Fig. 3c). Evaluation based on the other metrics (that is, relative error and absolute relative difference (ARD)) delivers similar results (Extended Data Fig. 6).

We benchmark the performance of miniQuant-L among three different sequencing protocols, including PacBio cDNA sequencing (cDNA-PacBio), ONT cDNA sequencing (cDNA-ONT) and ONT direct RNA-seq (dRNA-ONT) (Methods ‘Simulation data’). With GENCODE annotation, the overall performance of miniQuant-L using cDNA-PacBio data outperforms cDNA-ONT and dRNA-ONT across nine sequencing depths (0.5–30 million), with an average median MARD of 0.4054 versus 0.4480 and 0.4402, respectively (Fig. 3d). This gap is attributed to the lower alignment errors and ambiguities of cDNA-PacBio reads, because of the lower sequencing error rate (1.12% versus 5.73% and 9.94% for cDNA-ONT and dRNA-ONT, respectively) and longer mean read length (2,732 bp versus 885 bp and 1,198 bp for cDNA-ONT and dRNA-ONT, respectively) (Methods ‘Simulation data’). When the sample-specific annotation is used, the errors are all reduced substantially (average median MARD 0.1411, 0.1710 and 0.1731 for cDNA-PacBio, cDNA-ONT and dRNA-ONT, separately), and the variance across nine sequencing depths becomes far smaller for all three protocols, underscoring the larger contribution of sample-specific annotation for gene isoform quantification over the choice of long-read sequencing protocols.

### Sampling error is critical in long-read-based quantification

Although long reads reduce the quantification error derived by data deconvolution (that is, deconvolution error), long-read-based quantification is limited by the substantial sampling error that results from the relatively low throughput given the current cost and yield.

For example, kallisto, with 10 million short-read pairs, reports smaller MARD than miniQuant-L, with 1 million long reads on 75.80% (24,420 of 32,215) genes, many (48.31%, 11,797 of 24,420) of which can be grouped into three sets (Fig. 3e and Supplementary Note 5): Set 1 (4,596 genes) with low abundance (0 < log_2_(TPM + 1) ≤ 2) and very large difference of errors between kallisto and miniQuant-L (−1 ≤ ΔMARD ≤ −0.7), Set 2 (1,845 genes) with low abundance (0<log_2_(TPM + 1) ≤ 2) and mild difference of errors (−0.2 ≤ ΔMARD < 0) and Set 3 (5,356 genes) with high abundance (3 ≤ log_2_(TPM + 1) ≤ 6) and considerable difference of errors (−0.35 ≤ ΔMARD < 0). The sampling error in the shallow sequencing depth dominates deconvolution error, which is remarkable in Set 1 and Set 2: their read numbers are relatively small due to low abundance (median TPM 0.28 and 1.14, respectively), whereas their deconvolution is relatively simple due to small K-values (median 1.00 and 1.51, respectively). In particular, the ΔMARD of Set 1, which is of extremely low abundance, is close to −1, because there are very few or no long reads but a reasonable number of short reads generated from these genes (for example, see gene *OR1I1* in Fig. 3f, left). Both K-values and abundance in Set 3 (median K-value 10.29 and median TPM 19.86) are far higher than in Set 1 and Set 2, so long-read-based quantification is more favorable in Set 3 than short-read-based quantification, which narrows the gap between their quantification errors. However, the total number of long reads is very low and there are many isoforms in the genes with high K-values, so some isoforms may contain few or no long reads and thus very high MARD (for example, see gene *GCLC* in Fig. 3f, right). Therefore, the MARD of miniQuant-L is still smaller than kallisto (median ΔMARD −0.1787) in Set 3.

**Extended Data Fig. 7:**
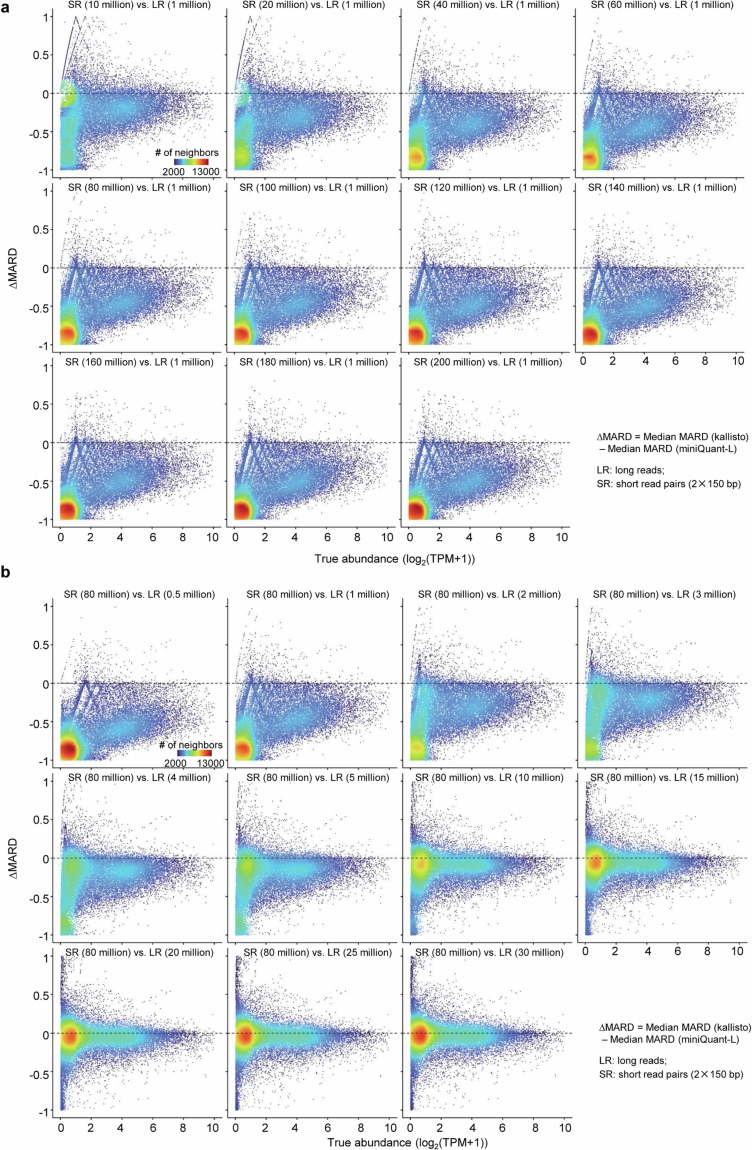
Comparisons of MARD between kallisto and miniQuant-L with respect to different sequencing depths. **a**, Comparisons of MARD between kallisto and miniQuant-L across 11 distinct combinations of long- and short-read sequencing depths. Scatter plots depicting ∆MARD versus true gene isoform abundance. Long reads are fixed at 1 million, while the number of short read pairs increases from 10 million to 200 million. **b**, Comparisons of MARD between kallisto and miniQuant-L across 11 distinct combinations of long- and short-read sequencing depths. Scatter plots depicting ∆MARD versus true gene isoform. Short read pairs are fixed at 80 million, while the number of long reads increases from 0.5 million to 30 million.

As the size of long reads remains fixed at 1 million while short reads increase, the difference of quantification errors (that is, |ΔMARD|) enlarges extensively (Extended Data Fig. 7a). In particular, most genes in Set 2 shift to Set 1, where the isoforms of low-expressed genes can be quantified with enhanced accuracy by increasing short reads but are still dropped out by long reads. ΔMARD becomes stable beyond 80 million short-read pairs. When the size of short reads is fixed at 80 million read pairs and long reads increase, |ΔMARD| shrinks dramatically and Set 1 shifts to Set 2 (Extended Data Fig. 7b). With 20 million long reads or more, Set 1 of the drop-out genes becomes negligible and ΔMARD distribution is almost symmetric around 0, that is, short-read- and long-read-based quantification errors plateau at a comparable level (Fig. 3e, middle).

The data size for obtaining quantification error plateau varies with respect to K-values. Considering two special cases of low K-value (≤2) and high K-value (>25), long-read-based quantification error plateau at the same sequencing depth (20 million) but short-read-based one plateau at different thresholds (60 million and 120 million, respectively) (Fig. 3g). It highlights that long-read-based quantification is less influenced by the uncertainty of data deconvolution than short-read-based quantification.

Considering the current typical depth (5 million long reads and 40 million short-read pairs), long-read-based quantification still underperforms for 78.50% (25,290/32,215) genes (ΔMARD < 0), where Set 1 and Set 3 are of considerable size and error (Fig. 3e, right). In sum, gene isoform quantification with long reads alone suffers from relatively low throughput and remains error-prone for low-expressed genes and isoforms.

### Gene- and data-specific integration of long and short reads

**Fig. 4:**
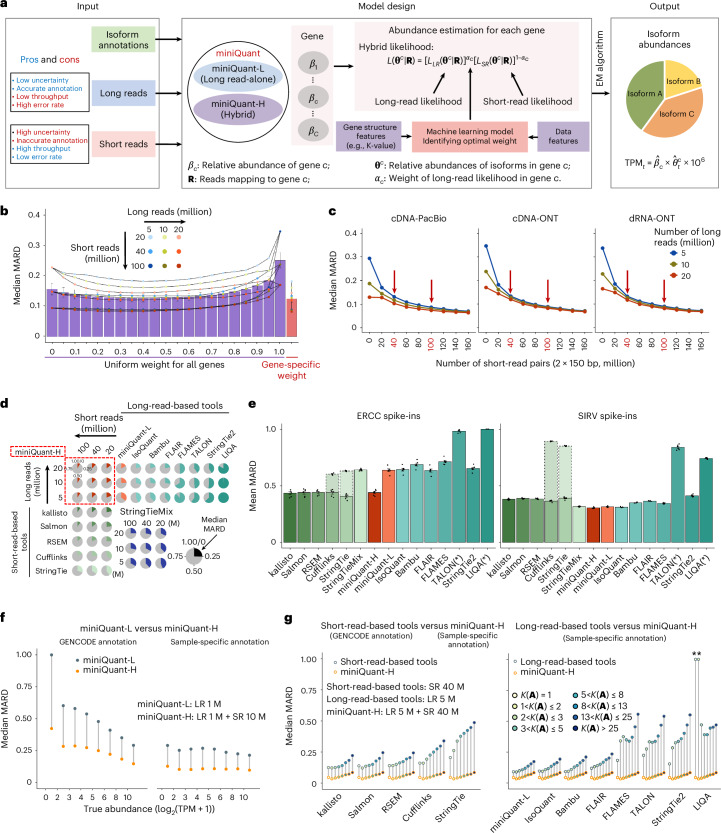
MiniQuant-H improves the accurate quantification of gene isoform. **a**, Schematic illustration of miniQuant to estimate gene isoform abundance. Left, miniQuant leverages the strengths of long and short reads. Middle, miniQuant-L and miniQuant-H are developed for two data scenarios. The miniQuant-H integrates the likelihood of both long and short reads with gene- and data-specific weight *α*_c_, which is determined by a machine learning model with data and gene structure features (for example, K-value) as input. Right, gene isoform abundances estimated from the hybrid likelihood using the EM algorithm. **b**, Comparison of the median MARD by miniQuant-H with uniform (from 0 to 1, purple) and gene-specific (red) weight across combinations of short-read and long-read sequencing depths (*n* = 9 combinations) under GENCODE annotation. **c**, Lines represent median MARD by miniQuant-H with increasing short-read sequencing depths under GENCODE annotation. Long reads across three sequencing depths with three protocols (cDNA-PacBio, cDNA-ONT and dRNA-ONT) are used. Red arrows, recommended combinations of sequencing depths. **d**, Comparison of the median MARD by five short-read tools, eight long-read tools and miniQuant-H across three short-read and three long-read sequencing depths under GENCODE annotation. **e**, Barplot showing the mean MARD of ERCC (left) and SIRV (right) spike-in transcripts of SIRV-set4 from the LRGASP consortium (*n* = 6 biological replicates). Bars within the dashed line indicate the de novo sample-specific annotation identified by Cufflinks and StringTie. Asterisk, technological issues specified in Supplementary Note 6. **f**, Dot chart represents the median MARD by miniQuant-L and miniQuant-H with different expression levels under GENCODE (left) and sample-specific (right) annotations. **g**, Dot chart showing the median MARD by short-read-based (left), long-read-based (right) and miniQuant-H tools within different K-value groups. Asterisk, LIQA does not quantify genes with single isoform. LR, long reads; SR, short-read pairs (2 × 150 bp). M, million.

**Extended Data Fig. 8:**
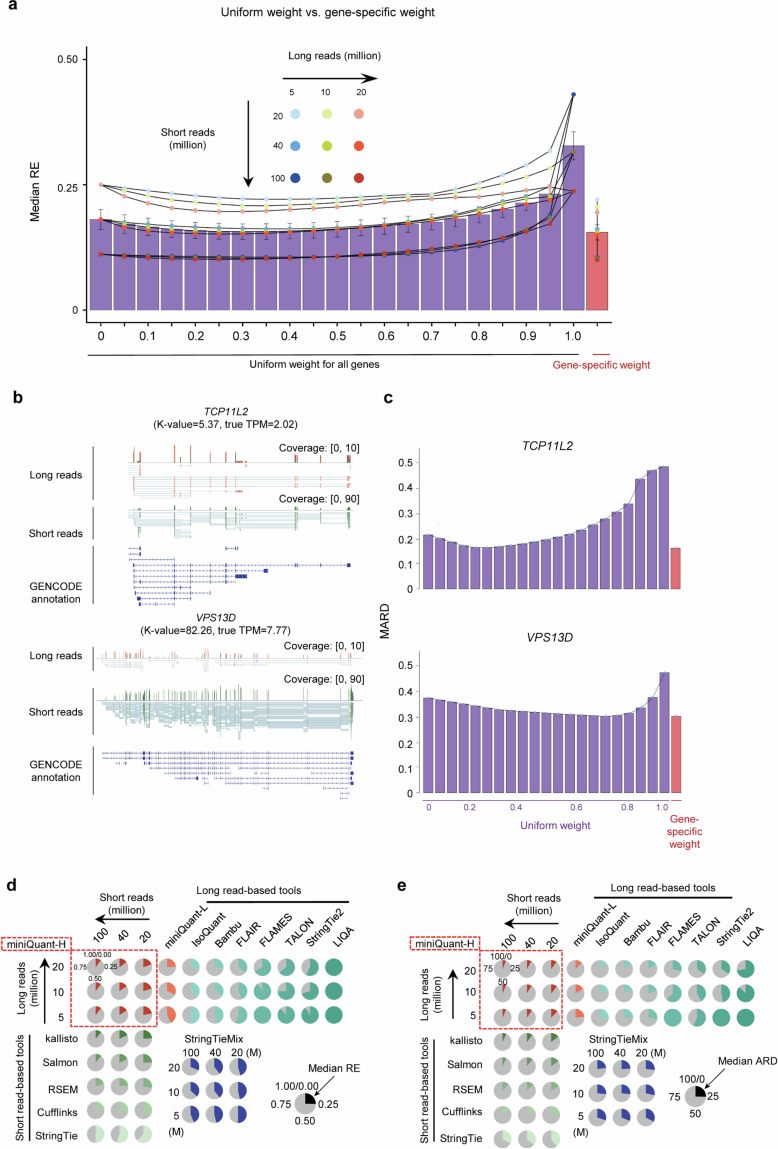
Performance of miniQuant-H on additional metrics and example genes. **a**, Comparison of the median RE between miniQuant-H with uniform (0 to 1, purple) and gene-specific (red) weight across combinations of three short-read and three long-read sequencing depths (n = 9 combinations) under GENCODE annotation. The quantified expression level of miniQuant-H is equal to miniQuant-L when the uniform weight is 1. RE: relative error. In bar plots, data are presented as mean values ± standard error. **b**, Coverage tracks and gene isoform structures of two indicated genes, *TCP11L2* and *VPS13D*. The K-value and TPM were labeled in the bracket under the gene symbols. **c**, Comparison of the MARD of two indicated genes between models with uniform (from 0 to 1, purple) and gene-specific (red) weight under GENCODE annotation. The quantified expression level of miniQuant-H is equal to miniQuant-L when the uniform weight is 1. A combination of 5 million long reads and 40 million short read pairs is used as input for miniQuant-H. **d-e**, Comparison of the median RE (**d**, gene level) and median ARD (**e**, gene isoform level) estimated from five short-read, eight long-read, mix mode of StringTie, and miniQuant-H tools across three short-read and three long-read sequencing depths under GENCODE annotation. Median REs shown here are from 0 to 1. RE, relative error. ARD: absolute relative difference.

Integration of long reads with short reads can improve deconvolution error and sampling error, respectively. Because sampling error depends on sequencing depth and gene isoform abundance, and deconvolution error depends on the complexity of read-isoform alignments (that is, K-value), a sophisticated integration should be gene- and data-specific. To this end, we developed miniQuant-H (that is, a hybrid mode of miniQuant), to accurately quantify gene isoforms, where a learning-based weighting scheme is customized for each gene given input data features and gene structure features, including K-value to achieve an optimal balance of long reads and short reads (Fig. 4a and Supplementary Note 4; Methods ‘Community-wise gene isoform abundance estimation model’). Across a large range of combinatorial sequencing depths of long reads (5, 10, 20 million) and short reads (20, 40, 100 million), miniQuant-H consistently reports lower quantification errors when gene- and data-specific weights are applied compared with a uniform weight across all genes, and both outperform the quantifications by long reads alone and short reads alone (that is, with uniform weights 1 and 0, respectively) (Fig. 4b; Extended Data Fig. 8a; Methods ‘Community-wise gene isoform abundance estimation model’). An integration weight close to 1 is more likely to be chosen by miniQuant-H for genes with complex isoform structures, where quantification error is contributed largely by deconvolution error and thus long reads are favorable to reduce it. For example, with 5 million long reads and 40 million short-read pairs, the gene *VPS13D* has a high K-value (82.26) and relatively high abundance (TPM 7.77), so long reads are heavily weighted (0.75) by miniQuant-H to obtain a minimum MARD (Extended Data Fig. 8b,c), that is, long reads contribute more than short reads to improving the quantification of this complex gene. In contrast, the optimal weight of long reads is as small as 0.25 to quantify the gene *TCP11L2* with low K-value (5.37) and relatively low abundance (TPM 2.02), because long reads are not much needed to resolve deconvolution with a low K-value but short reads with higher throughput are more favorable to reduce sampling error caused by low abundance.

Among three common types of long reads, cDNA-PacBio delivers the best quantification when integrated with short reads by miniQuant-H, which is the same as the benchmark of the quantification by long reads alone due to higher sequencing accuracy and longer length of cDNA-PacBio data (Figs. 3d and 4c).

Regardless of the type of long reads, quantification error decreases substantially with respect to the size of integrated short reads and the improvement becomes marginal and negligible beyond 40 million and 100 million short-read pairs, respectively (Fig. 4c). For example, miniQuant-H integrates 5 million cDNA-ONT long reads with 40 million and 100 million short-read pairs reports the median MARD 0.1333 and 0.0887, respectively, while the median MARD of miniQuant-L is as high as 0.3466 (Fig. 3d). Hence, in perspective of cost efficiency, the addition of 40–100 million short-read pairs is suggested for improving gene isoform quantification with long reads. The results are consistent across a wide range (from 5 to 20 million) of long reads.

We compare the performance of miniQuant-H with five short-read- and eight long-read-based gene isoform quantification software, including miniQuant-L, across multiple depths of long reads and short reads (Fig. 4d; Methods ‘Simulation data’). Overall, miniQuant-H achieves a lower average median MARD (0.1249) than all short-read- (0.1505–0.3555) and long-read-based tools (0.2515–0.9394). The evaluation based on the other error indices relative error and ARD shows similar results (Extended Data Fig. 8d,e; Methods ‘Performance evaluation’). When miniQuant-H adopts the sample-specific annotation that can be constructed by long reads accurately^13,14,49,50^, the average median MARD is as low as 0.0581.

The superior performance of miniQuant-H is further validated by the real data (8.20–17.17 million cDNA-ONT and 49.04–55.01 million 2 × 100 bp Illumina) from two sets of synthetic spike-in transcripts (External RNA Controls Consortium (ERCC) and spike-in RNA variant (SIRV)) with known relative abundance from the LRGASP consortium^13^ (Supplementary Fig. 3 and Supplementary Table 4; Methods ‘Real data’). All 92 ERCC transcripts contain single isoforms so there is no deconvolution error. However, they span a concentration range of six orders of magnitude and thus sampling error is critical for low-expressed isoforms in long-read-based quantification. Therefore, the long-read-based tools (except for the technical issues of TALON and LIQA; Supplementary Note 6) underperform the short-read-based ones (average mean MARD 0.6348–0.7134 versus 0.4066–0.4419; Fig. 4e, left), whereas miniQuant-H outputs the comparable accuracy with the short-read-based ones (average mean MARD 0.4403).

Being distinct with ERCC, 44 SIRV transcripts from four gene loci are of the same abundance at a high level but are of different gene isoform complexities (Supplementary Fig. 3 and Supplementary Note 7). Long reads are favorable to reducing the deconvolution errors of these targets. Therefore, the long-read-based tools (except for the technical issues of TALON and LIQA; Supplementary Note 6) output lower errors for SIRV than ERCC (average mean MARD 0.3111–0.4112 versus 0.6348–0.7134) and also generally outperform the short-read-based ones (average mean MARD 0.3649–0.3915 in SIRV) (Fig. 4e, right), while miniQuant-H outputs the lowest average mean MARD 0.3047. Overall, miniQuant-H is among the best quantification tools for both ERCC and SIRV transcripts with different transcript complexity and abundance, as it integrates the strengths of both short reads and long reads. It should be noted that these short-read-based quantifications use the groundtruth annotation as the input, and thus the error difference with the long-read-based quantification is not remarkable. When the short-read-based quantification adopts the annotation identified by short reads in SIRV targets, the error increases strikingly (average mean MARD 0.8579 and 0.8509 for Cufflinks and StringTie, respectively; Fig. 4e, right). In sum, so far, an optimal strategy for gene isoform quantification is to integrate long reads and short reads (for example, by miniQuant-H) with an accurate sample-specific annotation that could be constructed by long reads alone^13,14,42,44,45,47,49–54^ or again by integration of long reads and short reads^13,14,42,45,47,49,50,52^.

The mix mode of StringTie (StringTieMix)^55^ integrates long reads and short reads for gene isoform quantification by assigning each short read to a unique isoform that is supported by the greatest number of long reads among all compatible isoforms. Compared with the short-read-alone mode of StringTie, StringTieMix with additional long reads performs only slightly better in the simulated datasets (average median MARD 0.3555 versus 0.3322; Fig. 4d) and the SIRV spike-ins (average mean MARD 0.3915 versus 0.3175), and even underperforms in the ERCC spike-ins (average mean MARD 0.4066 versus 0.6405). These results indicate the necessity of a sophisticated model for the integration of long reads and short reads.

The advantage of miniQuant-H can be dissected and illustrated with respect to gene abundance and K-value, considering the improvement of sampling error and deconvolution error, respectively. In particular, miniQuant-H extensively addresses the above-mentioned limitation of long-read-based quantification in the context of low-throughput and low-expressed targets. Taking shallow data (1 million long reads) as an example, miniQuant-H integrates it with 10 million short-read pairs and reduces the median MARD of miniQuant-L by 0.4471 and 0.1524 with the input of GENCODE and sample-specific annotations, respectively. The improvement shrinks as gene abundance increases when GENCODE annotation is used (Fig. 4f). Notably, for low-expressed genes (log_2_(TPM + 1) ≤ 2), miniQuant-H achieves a remarkable median MARD reduction (0.5775). Hence, these results underscore the importance of adding the cost-effective short reads to the low-throughput long reads towards accurate gene isoform quantification. Of note, the improvement remains considerable yet within a narrow range (0.1172–0.1634) regardless of gene abundance when the sample-specific annotation is adopted, because the more accurate annotation has dramatically enhanced the quantification of all gene isoforms.

**Extended Data Fig. 9:**
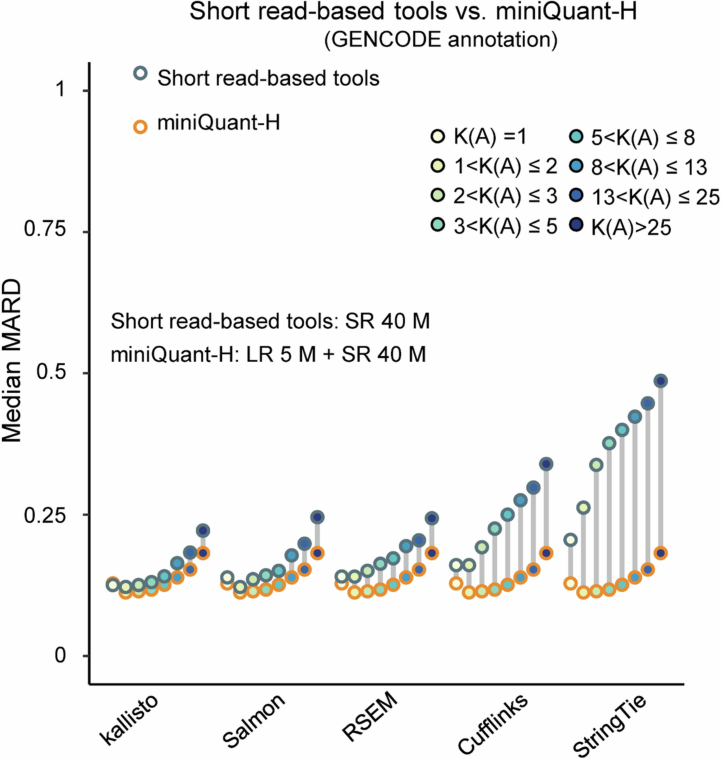
Comparison of MARD between short read-based tools and miniQuant-H under GENCODE annotation. The dot chart shows the median MARD of short read-based tools and miniQuant-H within different K-value groups under GENCODE annotation. 40 million short reads are used for short read-based tools, and a combination of 5 million long reads and 40 million short reads are used as input for miniQuant-H. LR, long reads. SR, short-read pairs (2×150 bp). M, million.

In parallel, miniQuant-H solves the long-standing challenge of quantifying the isoforms of complex genes. For instance, in the context of 5 million long reads and 40 million short-read pairs, the error reduction by miniQuant-H over all five short-read-based tools generally increases with the K-value (Fig. 4g and Extended Data Fig. 9). When the K-value exceeds 25, the reductions of the average median MARD across the five short-read-based tools reach 0.2224 and 0.1252 with the input of sample-specific and GENCODE annotations in miniQuant-H, respectively. The particular improvement of genes with high K-value is also demonstrated in the real data of spike-in RNAs: with additional long reads, miniQuant-H makes more clear improvement over the short-read-based tools on the set of complicated targets with high K-values (that is, SIRV set with median K-value 15.80) than the set of simple targets (that is, the ERCC set with median K-value 1, where all ERCC genes have single isoforms) (Fig. 4e).

Since the groundtruth annotation of spike-in RNAs is used by all tools, the improvement by miniQuant-H resulted largely from the addition of long reads to the quantification model, particularly for complicated genes. Consistent results exist in the simulation data (Supplementary Note 8): in the scenario of 5 million long reads and 40 million short-read pairs, when all tools use the sample-specific annotation, miniQuant-H improves the median MARD by 15.42% and 12.79% compared with kallisto and Salmon, respectively, in genes with K-value > 25, but only 12.35% and −1.48%, respectively, in genes with K-value = 1.

Comparing miniQuant-H with long-read-based tools, a similar positive association of the error reduction and K-value also exists: genes with high K-values typically contain many isoforms, resulting in an increasing probability of containing low-expressed isoforms (Fig. 3f, right). Even though exon–isoform structure is complex (that is, high K-value), the deconvolution of the added short reads can be guided by long reads in miniQuant-H and thus enhances quantification of isoforms with low expression. These results indicate the complicated interaction of sampling error and deconvolution error, which is associated with gene isoform abundance and K-value.

### Isoform switching during human ESC differentiation

**Fig. 5:**
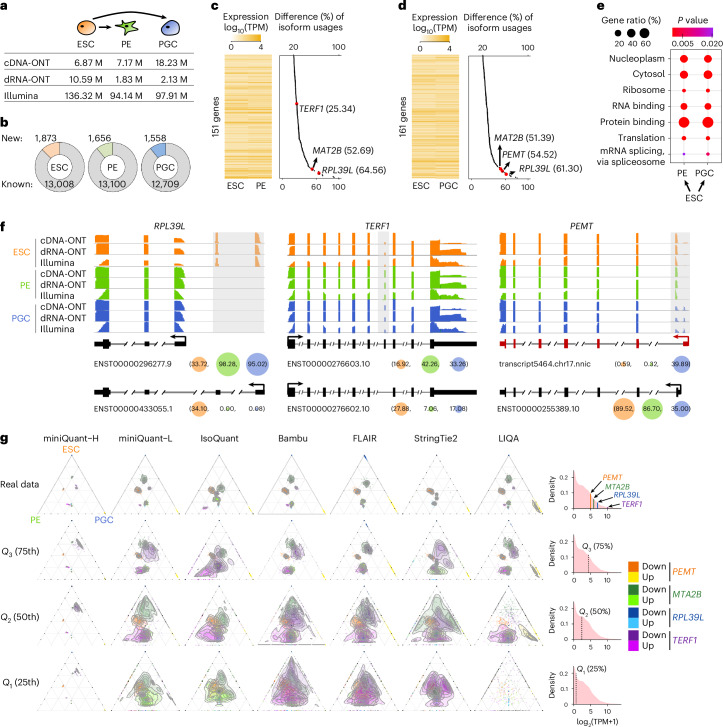
Dynamics of isoform switching during human ESC differentiation to PE and PGC. **a**, Number of long reads and short-read pairs. **b**, Number of gene isoforms with TPM ≥ 1. Known, annotated by GENCODE annotation; New, not annotated by GENCODE annotation. **c**,**d**, Left, expression heatmap of 151 genes with isoform switches from ESC to PE (**c**) and 161 genes from ESC to PGC (**d**). Right, for each gene, the maximal difference of the usage ratios (ranging from 0 to 100%) of its isoforms between ESC and PE (**c**) and between ESC and PGC (**d**). Results by cDNA-ONT and Illumina data are shown. **e**, GO analysis of 151 (ESC to PE) and 161 (ESC to PGC) isoform-switching genes identified in both differentiation trajectories (see Methods, ‘Analyses of isoform switching’). Only the GO terms with one-sided exact *P* value < 0.05 are shown. **f**, Three example genes with isoform switches. For each gene isoform, the usage ratios (ranging from 0 to 100%) in three samples by cDNA-ONT and Illumina data are specified within parentheses. Certain intron regions are condensed for readability. **g**, Ternary and contour plots showing the relative enrichment of the isoform usage ratios across ESC, PE and PGC for four genes with isoform switches (Fig. 5f and Extended Data Fig. 10d) and corresponding density. Each gene has two isoforms, where one is used predominantly in ESC (Down, deep color), and the other is used mainly in PE and/or PGC (Up, light color); 100 bootstrap sampling of cDNA-ONT reads for four genes was conducted, with sizes corresponding to real abundances of four genes (Real data) and quartiles (*Q*_3_, *Q*_2_, *Q*_1_) of all gene abundances in ESC. Isoforms used solely in one of the ESC, PE and PGC will be located at the triangle vertices without contour. The contour plot is not generated for extremely sparse points such as LIQA at the 25th percentile. The distribution of all gene abundances in ESC are shown on the right and the bootstrap sampling sizes are indicated correspondingly. Due to technological issues, the FLAMES and TALON are not shown here (Supplementary Note 13).

With data integration, quantification error reduction by miniQuant-H can greatly benefit the downstream qualitative analyses of gene isoforms, such as finding the subtle but meaningful alteration of isoform usage (that is, isoform switching), which is key to finding isoform-specific functions^56–58^. In the proof of concept, we apply miniQuant to investigate isoform switching during human ESC differentiation to PE^59^ and PGC^60^, which are generated by the in vitro differentiation platforms we have developed recently(Fig. 5a and Supplementary Table 5; Methods ‘Generation of sequencing data from ESC, DE, PE and PGC’). On average, 14,635 expressed (TPM ≥ 1) isoforms are detected in these three samples, and over 10.92% of them (ESC, 1,873 of 14,881; PE, 1,656 of 14,756; PGC, 1,558 of 14,267) are new ones that are not annotated by GENCODE (Fig. 5b).

**Extended Data Fig. 10:**
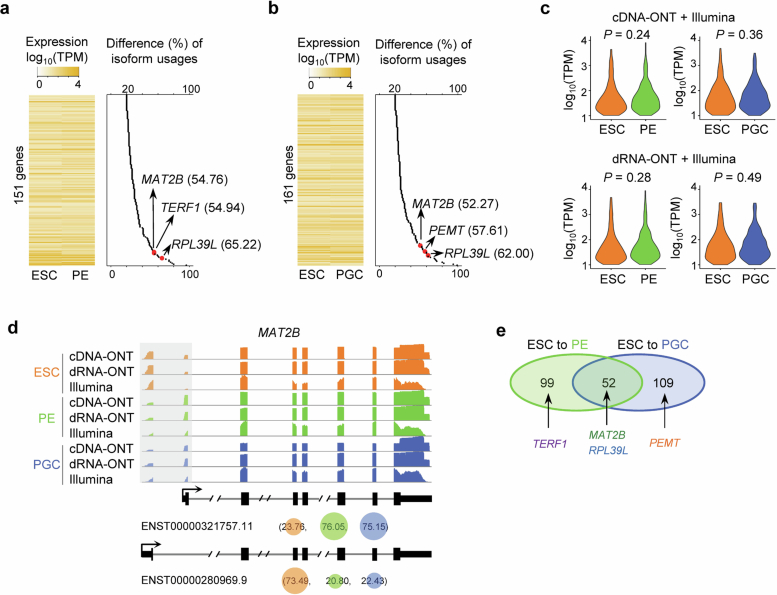
Isoform switching during the differentiation of human ESC to PE and PGC. **a**, Expression heatmap of 151 genes with isoform switches from ESC to PE (left); and for each gene, the maximal difference of the usage ratios (ranging from 0% to 100%) of its isoforms between ESC and PE are plotted (right). These results are based on analyses utilizing dRNA-ONT and Illumina data. **b**, Parallel to panel **a**, but presenting 161 genes with isoform switches from ESC to PGC. These results are based on analyses utilizing dRNA-ONT and Illumina data. **c**, Violin plots comparing the overall expression of isoform-switching genes during the differentiation ESC to PE (n = 151 genes, left) and PGC (n = 161 genes, right). The *P* values are calculated by the two-sided Wilcoxon rank-sum test. These results are based on analyses utilizing cDNA-ONT plus Illumina data (top), and dRNA-ONT plus Illumina data (bottom). **d**, Genome browser showing an example gene with isoform switches. For each gene isoform, the usage ratios (ranging from 0% to 100%) in three samples are specified within parentheses, which are based on analyses utilizing cDNA-ONT and Illumina data. To improve readability, certain intron regions are condensed. **e**, Overlap of isoform-switching genes identified in the two differentiation trajectories.

Across the two differentiation courses, 151 (ESC to PE) and 161 (ESC to PGC) genes with isoform switches are identified, respectively (Fig. 5c,d, Extended Data Fig. 10a,b and Supplementary Tables 6–9; Methods ‘Analyses of isoform switching’), yet their overall gene expression levels do not show clear changes (Extended Data Fig. 10c). For example, despite gene-level expression remaining relatively stable between ESC (TPM = 59.46) and PGC (TPM = 63.97), *MAT2B* undergoes marked isoform switching during differentiation (Extended Data Fig. 10d). *MAT2B* functions in maintenance and differentiation of human pluripotent stem cells^61^. Its two isoforms with alternative usages encode protein variants that differ by 20 amino acids at the N-terminal end and, although both can regulate cell growth, only the protein encoded by ENST00000280969.9 (dominantly in ESC) affects apoptosis^62^.

Functional enrichment analysis reveals that the protein products of these isoform-switching genes localize largely within the nucleoplasm and cytosol, participating in the regulation of mRNA splicing and translation by their RNA and protein-binding capabilities (Fig. 5e). Some of them are components of ribosomes, such as *RPL39L*, whose mouse homolog contributes to the pluripotency and differentiation of mouse ESCs^63^ and is required for spermatogenesis^64^ and male fertility^65^. *RPL39L* utilizes a distal promoter (ENST00000433055.1) specifically in ESC, whereas there is a substantial increase in the engagement of the proximal promoter (ENST00000296277.9) in PE and PGC (33.72% versus ≥95.02%; Fig. 5f, left).

Rather than being shared in both differentiation trajectories, some isoform switches are specific to the transition from ESC to PE (99 genes) or from ESC to PGC (109 genes) (Extended Data Fig. 10e). For example, the isoform switch of *TERF1* from ESC to PE skips an internal exon that alters the coding sequence (Fig. 5f, middle). *TERF1* regulates telomere length^66^, and its mouse homolog serves as a stem cell marker and is critical for both the induction and maintenance of pluripotency^67^. In addition, *PEMT*, which encodes a phosphatidylethanolamine N-methyltransferase linked to the quality of human sperm^68^, harbors isoform switches unique to the ESC to PGC differentiation (Fig. 5f, right). Moreover, a new *PEMT* isoform that is not annotated by GENCODE, is specifically activated in PGC. The predicted protein product of this new isoform (199 amino acids) lacks the initial 37 amino acids that are encoded in the dominant isoform in ESC and PE. This truncated region is predicted as a cytoplasmic domain by InterPro^69^. These isoform switches are supported not only by both cDNA-ONT and dRNA-ONT (Fig. 5f and Extended Data Fig. 10d), but also by several long-read-based quantification tools (Fig. 5g).

These important isoform switches in *PEMT*, *MAT2B*, *RPL39L* and *TERF1* may have been overlooked by long reads alone if they were not extremely highly expressed (the 82.09th–99.07th transcriptome-wide percentile and TPM from 30.60 to 1,077.09 in ESC) (Fig. 5g; Methods ‘Analyses of isoform switching’). Under the typical size of long reads (for example, 6 million cDNA-ONT), these isoform switches become ambiguous as the sequencing depth on these genes are downsampled to be equivalent to the ones with the 75th percentile abundance (TPM = 18.90) (Fig. 5g): the isoform quantification by long-read-based tools (except technical issues of FLAMES and TALON) becomes notably uncertain due to greater sampling error. When the gene abundance is at the 50th (that is, the median level) or 25th percentile, the isoform switching patterns are indistinguishable even using long-read-based tools (including miniQuant-L), especially for *TERF1*, which undergoes relatively minor isoform usages change (25.34%; Fig. 5c).

In contrast, miniQuant-H robustly reports clear and consistent isoform switching patterns across a large extent of gene abundance (Fig. 5g), because it integrates short reads to eliminate the drawback of the relatively low throughput of long reads. Indeed, important isoform switches may exist in genes with high-to-moderate expression level, which have been indicated by prevalent studies of alternative splicing in many genes^33,57,70,71^. Hence, so far, integration of long reads with short reads is an optimal strategy to accurately quantify gene isoforms and thus to uncover more isoform-specific expression and functions.

## Discussion

It is widely acknowledged that the alignment ambiguity of short reads, resulting from the complexity of gene isoforms, leads to critical quantification errors, and that long reads can mitigate this issue to some extent. Here, beyond the conventional descriptive conclusions, we investigated these problems with the K-value in a rigorous way so that we can implement miniQuant to rank genes with their risks in gene isoform studies and reduce gene isoform quantification errors by integrating long reads and short reads in a gene- and data-specific manner. We also illustrate the strengths of long reads in quantifying gene isoforms with high K-values.

However, despite long-read sequencing techniques being capable of generating reads over 100 kb, the presence of fragmented molecules and imperfections in library preparation (for example, RNA integrity and the completeness of reverse transcription) lead to a considerable proportion of fragmented reads in long-read RNA-seq (Fig. 1a, Supplementary Fig. 1b and Supplementary Note 9). Hence, deconvolution error remains a critical component of the quantification errors of gene isoforms. In contrast, sampling error that is associated with data size can be reduced as the throughput and cost of long-read sequencing are improving rapidly. The data size at which sampling errors plateau depends on the abundance and structure of gene isoforms as well as read length, although we have shown the thresholds as 80 million short-read pairs and 20 million long reads in the example of the human ESC transcriptome.

Given the current yield and cost of long reads, data integration remains the optimal approach to achieve accurate gene isoform quantification. In fact, integration of long reads and short reads has been demonstrated well in other problems, such as Telomere-to-Telomere genome assembly^72–76^ and gene isoform identification^13,14,42,45,47,49,50,52^. As shown, the addition of 40–100 million short-read pairs to long reads can substantially decrease the errors presented in the quantification by long reads alone, at only marginal cost compared with the expense of long reads.

In addition, gene isoform structure is crucial to the deconvolution error, so the use of sample-specific annotation instead of the reference annotation (for example, GENCODE) reduces quantification errors substantially, as shown in several tests mentioned above. Given that sample-specific annotation can be constructed using long reads and refined with short reads, data integration is compatible with both gene isoform identification and quantification and offers an end-to-end solution to transcriptome profiling. This strategy could be extended to single-cell long-read RNA-seq, which typically generates a paired set of short reads, which also calls for the development of another sophisticated method.

Beyond gene isoforms, transcripts from different genomic loci could also share critical amounts of repetitive sequences such as transposable elements (TEs). Therefore, the current implementation of miniQuant-H has been generalized to account for the ambiguity of read alignment across genes, where the balanced weight between long reads and short reads is determined at the ‘community’ level rather than at the gene level (Fig. 4a and Supplementary Note 4; Methods ‘Community construction’): each community contains genes and the corresponding isoforms that share read alignments so that the ambiguity of the origin of reads, that is, data deconvolution, exists only within a community. Therefore, TE-derived transcripts could also be quantified with miniQuant, although the specific features of TE-derived transcripts should be further considered to achieve optimal performance.

Besides these types of errors in the ‘ideal’ quantification model, a few other features of real data that can influence quantification, such as sequencing bias with respect to 5′/3′ ends, GC content and starting position, can also be incorporated into the joint likelihood function of miniQuant. Although certain factors have been considered by short-read-based tools^6,12,19,29,77–82^, similar efforts should also be made for long-read-based tools. If quantification errors can be dissected and considered by downstream quantitative analysis models, such as differential expression analysis, co-expression network construction and cell clustering in single-cell long-read RNA-seq, it is possible to enhance overall performance.

However, many upstream steps, including data generation and data preprocessing, can give rise to other types of data variations that are difficult to model. For example, RNA secondary structure, especially for long transcripts, could result in imperfections in reverse transcription for cDNA sequencing or incompleteness/errors of direct RNA-seq. The other sources of data collection errors include internal polyA and internal base pairing with ‘primer/oligo’ during reverse transcription and PCR. In addition, alignment error, such as the misalignment around micro-exons or alternative splice sites with small differences, is harmful to quantification: the particularly high quantification errors in the spike-in transcripts of SIRV5 and SIRV7 are both caused by the misalignment of long reads between isoforms, where alternative splice sites share only very minimal differences (for example, only 7 bp between two isoforms of SIRV5, Supplementary Figs. 4 and 5 and Supplementary Note 7). Consequently, the quantification error decreases slightly with the accuracy of long reads (Figs. 3d and 4c). Therefore, the rapidly advancing long-read sequencing techniques, with their rising yields, enhanced accuracy and decreasing cost, will be very useful for both transcriptome identification and quantification.

## Methods

### K-value and miniQuant method

#### K-value (generalized condition number)

Consider a gene consisting of *T* isoforms with relative abundances ${{\mathbf{\uptheta} }=\left({\theta }_{1},{\theta }_{2},\cdots ,{\theta }_{T}\right)}^{{\prime} }$. The fractions of nucleotides can be calculated as ${\mathbf{\upphi}}_{t}=\frac{{\theta }_{t}{\widetilde{l}}_{t}}{\mathop{\sum }\nolimits_{j=1}^{T}{\theta }_{j}{\widetilde{l}}_{j}},$ where ${\widetilde{l}}_{t}$ is the effective length of gene isoform *t*. Further assume the gene consists of *S* disjoint regions defined by read-isoform alignment (Supplementary Notes 1 and 2)^49^, and the read count for region *s* is *N*_*s*_, 1 ≤ *s* ≤ *S*. $N=\mathop{\sum }\nolimits_{s=1}^{S}{N}_{s}$ is the total number of reads mapped to the gene in the sample, ${\widetilde{r}}_{s}$ is the effective length of region *s* (Supplementary Note 2) and $1\left\{s\in t\right\}$ is the indicator function that region *s* is contained in gene isoform *t*. The least squares solution for **ϕ** of the linear system **b** = **Aϕ** is then calculated, where ${\textbf{b}}={\left({b}_{1},{b}_{2},\cdots ,{b}_{S}\right)}^{{{\prime} }}={(\frac{{N}_{1}}{N},\frac{{N}_{2}}{N},\cdots ,\frac{{N}_{S}}{N})}^{{{\prime }}}$ are the read proportions for regions, and ${\textbf{A}}=\left({A}_{{st}}\right)\in {{\mathbb{R}}}^{S\times T}$ is the read-isoform alignment conditional probability matrix with ${A}_{{st}}=\frac{{\widetilde{r}}_{s}}{{\widetilde{l}}_{t}}\times 1\left\{s\in t\right\}$ (Supplementary Note 1).We propose the generalized condition number, K-value, as a key quantitative index of a gene’s quantification error. Specifically, K-value is defined as

$$K\left({\textbf{A}}\right)=\frac{{\sigma }_{\max }}{{\sigma }_{r}},$$

where *σ*_max_ and *σ*_*r*_ are the maximum and minimum positive singular values of **A**, respectively (Supplementary Note 1). The calculation of K-values takes into account both the gene isoform structure and the read length of data. The K-values presented in this study are calculated with a 150-bp read length and a filtered GENCODE annotation (see Methods ‘Annotations and datasets’ for details) unless otherwise specified.

### MiniQuant method

#### Community construction

Assume sequencing produces *N* short reads and *M* long reads. MiniQuant categorizes reads into *C* gene communities, with each read mapped to at most one community, that is, there are no shared reads across communities. Within a community, a read can be mapped to multiple gene isoforms or genes.

Consider a specific community *c* containing *N*^*c*^ short reads and *M*^*c*^ long reads that can be mapped to *T*^*c*^ gene isoforms. Within community *c*, denote the abundances of the *T*^*c*^ gene isoforms as ${{\mathbf{\uptheta}} }^{c}={\left({\theta }_{1}^{c},\ldots ,{\theta }_{{T}^{c}}^{c}\right)}^{{\prime} }$ such that $\mathop{\sum }\nolimits_{t=1}^{{T}^{c}}{\theta }_{t}^{c}=1$.

#### Community-wise gene isoform abundance estimation model

In the hybrid mode of miniQuant (miniQuant-H), the following likelihood function is used for quantification

$$L\left({{\mathbf{\uptheta} }}^{c}|{\textbf{R}}\right)={\Big[{L}_{{{\rm{LR}}}}\left({{\mathbf{\uptheta} }}^{c}|{\textbf{R}}\right)\Big]}^{{\alpha }_{c}}{\Big[{L}_{{{\rm{SR}}}}\left({{\mathbf{\uptheta} }}^{c}|{\textbf{R}}\right)\Big]}^{1-{\alpha }_{c}},$$

where ${\textbf{R}}={\left({R}_{1},\cdots ,{R}_{{N}^{c}+{M}^{c}}\right)}^{{\prime} }$ are sequences of all reads in community *c*, ${L}_{{{\rm{LR}}}}\left({{\mathbf{\uptheta} }}^{c}|{\textbf{R}}\right)$ and ${L}_{{{\rm{SR}}}}\left({{\mathbf{\uptheta} }}^{c}|{\textbf{R}}\right)$ are likelihood functions of long reads and short reads, respectively, and *α*_c_ is a community-specific weight for long reads obtained by a machine learning model. In the likelihood of long reads, an isoform-specific read length distribution is adapted (Supplementary Note 4). Note that *α*_c_ = 1 corresponds to the long-read-alone mode of miniQuant (miniQuant-L).

For a given *α*_c_, the maximum likelihood estimate of **θ**^*c*^ (denoted as ${\hat{\mathbf{\uptheta}}}^{{c},{\alpha}_{c}}$) is obtained using the expectation-maximization (EM) algorithm^83^ (see Supplementary Note 10 for details).

Based on simulation data (Methods ‘Simulation data’) with known true relative abundance **θ**^*c*^, the actual MARDs ${{\textbf{y}}}_{c}=({y}_{c,{\alpha }_{c}})$ are calculated using ${\hat{\mathbf{\uptheta}}}^{{c},{\alpha}_{c}}$ for each community *c* with weight ${\alpha }_{c}\in {{\mathcal{A}}}_{c}=\{\mathrm{0,0.05},.,\mathrm{0.95,1}\}$. The input vector **x**_*c*_ consists of read and gene structure features of community *c* (Supplementary Table 10). For a combination of *N* short reads and *M* long reads, a multi-output regression gradient boosting model ${\mathcal{g}}_{N,M}$ implemented by XGBoost (v.2.0.3)^84^ is trained to predict **y**_*c*_ using **x**_*c*_. The hyperparameters are shown in Supplementary Table 11.

For a community with input **x**_*c*_, the predicted MARDs ${\hat{{\textbf{y}}}}_{c}=({\hat{y}}_{c,{\alpha }_{c}})_{{\alpha }_{c}\in {{\mathcal{A}}}_{c}}$ and the optimal community-specific weight ${\alpha }_{c}^{{\rm{optimal}}}$ in ${{\mathcal{A}}}_{c}$ can be found by

$${\hat{{\textbf{y}}}}_{c}={{\mathcal{g}}}_{N,M}\left({{\textbf{x}}}_{c}\right),$$

$${\alpha }_{c}^{{\rm{optimal}}}={{\rm{argmin}}}_{{\alpha }_{c}}\left({\hat{y}}_{c,{\alpha }_{c}}\right).$$

The estimated relative abundances ${\hat{\mathbf{\uptheta}}}^{c}$ in community *c* are then chosen as

$${\hat{\mathbf{\uptheta}}}^{c}={\hat{\mathbf{\uptheta}}}^{{c},{\alpha}_{c}^{\rm{optimal}}}.$$

Considering the variety of length distributions, read truncation profiles, and error profiles in different sequencing platforms and protocols, we have built protocol-specific models available online at https://miniquant.s3.us-east-2.amazonaws.com/pretrained_models.tar.gz. Pretrained models for three different sequencing protocols (cDNA-PacBio, cDNA-ONT and dRNA-ONT) with 15 combinations of different short-read and long-read sequencing depths are provided. When applied to real data, the pretrained model with sequencing depths that most closely resemble the real dataset would be chosen.

Given the distinct gene isoforms structure features for spike-in transcripts from regular ones, spike-in specific models (available online at https://miniquant.s3.us-east-2.amazonaws.com/SIRV_pretrained_models.tar.gz) are also trained by further fine-tuning the cDNA-ONT pretrained model on the spike-in transcripts. Six spike-in specific models are trained by withholding one real spike-in replicate from training data for evaluation.

#### Calculation of sample-wise gene isoform expression level

Denote the community-wise relative abundances of the *C* communities as ${\mathbf{\upbeta}} ={\left({\beta }_{1},\ldots ,{\beta }_{C}\right)}^{{\prime} }$, such that $\mathop{\sum }\nolimits_{c=1}^{C}{\beta }_{c}=1$. In community *c*, the estimated relative abundances of the *T*^*c*^ gene isoforms are ${\hat{\mathbf{\uptheta} }}^{c}={\left({\hat{\theta }}_{1}^{c},\ldots ,{\hat{\theta }}_{{T}^{c}}^{c}\right)}^{{\prime} }$, with $\mathop{\sum }\nolimits_{t=1}^{{T}^{c}}{\hat{\theta }}_{t}^{c}=1$. Let ${\widetilde{l}}_{t}^{c}$ be the effective length of gene isoform $t\:(1\le t\le {T}^{c})$ in community *c*. Then, the TPM value of gene isoform *t* in community *c* can be calculated by

$${{\rm{TPM}}}_{t}^{c}={\hat{\beta }}_{c}\times {\hat{\theta }}_{t}^{c}\times {10}^{6},$$

where ${\hat{\beta }}_{c}=\frac{{M}^{c}}{\mathop{\sum }\nolimits_{i=1}^{C}{M}^{i}}$ if estimated by long reads, or ${\hat{\beta }}_{c}=\frac{\mathop{\sum }\nolimits_{t=1}^{{T}^{c}}\frac{{N}_{t}^{c}}{{\widetilde{l}}_{t}^{c}}}{\mathop{\sum }\nolimits_{c=1}^{C}\mathop{\sum }\nolimits_{i=1}^{{T}^{c}}\frac{{N}_{i}^{c}}{{\widetilde{l}}_{i}^{c}}}$ with ${N}_{t}^{c}={N}^{c}\times \frac{{\hat{\theta}}_{t}^{c}{\widetilde{l}}_{t}^{c}}{\mathop{\sum }\nolimits_{i=1}^{{T}^{c}}{\hat{\theta}}_{i}^{c}{\widetilde{l}}_{i}^{c}}$ if estimated by short reads. In this study, miniQuant-L estimates ${\hat{\beta }}_{c}$ by long reads, while miniQuant-H estimates ${\hat{\beta }}_{c}$ by short reads.

### Existing methods

MiniQuant is compared with five short-read-based quantification tools (kallisto^18^, Salmon^19^, RSEM^29^, Cufflinks^31^, StringTie^30^), seven long-read-based quantification tools (IsoQuant^42^, Bambu^43^, FLAIR^45^, FLAMES^44^, TALON^47^, StringTie2 (ref. ^48^), LIQA^46^) and the mix mode of StringTie (StringTieMix)^55^ that integrates short reads and long reads to evaluate its effectiveness. See Supplementary Table 12 for details about the application of existing methods.

Several technical issues have been identified during the application of existing methods. Long-read-based quantification methods return fewer isoforms than indicated in the annotation (Supplementary Note 6). In subsequent analyses, these missed isoforms are treated as having an expression level of 0. Additionally, long-read-based methods TALON, FLAIR, FLAMES and LIQA provide quantification results in counts instead of TPM. For these methods, the counts are transformed into TPM by ${{\rm{TPM}}}_{t}=\frac{{N}_{t}}{{\sum }_{i=1}^{T}{N}_{i}}\times {10}^{6}$, where *N*_*t*_ represents the number of long reads assigned to isoform *t*.

### Annotations and datasets

#### Reference genome and gene annotations

Datasets were aligned to the hg38 reference genome using minimap2 (ref. ^85^) (v.2.24-r1122) for long reads and Hisat2 (ref. ^86^) (v.2.1.0) for short reads. Short reads were also mapped to transcriptome using Bowtie2 (ref. ^87^) (v.2.4.1). The parameters used for minimap2, Hisat2 and Bowtie2 are shown in Supplementary Table 12.

A filtered GENCODE annotation (v.39) was used in this study unless otherwise specified (Supplementary Note 11).

Additionally, sample-specific annotations for real datasets were constructed by selecting gene isoforms with TPM ≥ 1 in the quantification results generated using kallisto with the above filtered GENCODE annotation. These sample-specific annotations were designed to mimic the gene isoforms that have sufficient expression level to be identified using long reads and avoid the bias caused by using a specific identification tool.

When analyzing synthetic ERCC and SIRV spike-in transcripts, the spike-in transcripts were added into the reference genome and gene annotations.

#### Real data

Real datasets from three consortia (GTEx^24,25^, TCGA^25,26^ and ENCODE^27^); data from synthetic ERCC and SIRV spike-in transcripts from the LRGASP consortium^13^ (Supplementary Tables 1–4) and ESC, PE and PGC data (Supplementary Table 5; Methods ‘Generation of sequencing data from ESC, DE, PE and PGC’) were analyzed using miniQuant and existing methods.

Real data ESC, definitive endoderm (DE), GM12878 (ref. ^88^) and K562 (ref. ^89^) were used to generate simulation datasets. The gene K-values in GTEx and TCGA datasets were calculated using their corresponding versions of GENCODE annotations. The true relative abundances of transcripts in a real dataset (also called groundtruths, in terms of TPM) were quantified using kallisto, except for ERCC and SIRV spike-in transcripts. The groundtruths for ERCC and SIRV spike-in transcripts were established based on their concentrations within the samples. The concentrations were obtained from the ‘conc (amoles/µl)’ column in the design table (https://www.lexogen.com/wp-content/uploads/2021/06/SIRV_Set4_Norm_sequence-design-overview_20210507a.xlsx). The concentrations of ERCC transcripts were scaled to yield a sum of 10^6^ and are then treated as the groundtruths. The same procedure was applied to determine the groundtruths for SIRV transcripts.

See Supplementary Note 12 for details about the estimation of long-read characteristics from real data.

#### Simulation data

Various short-read and long-read RNA-seq datasets (cDNA-Illumina, cDNA-PacBio, cDNA-ONT, dRNA-ONT) were generated with GENCODE annotation and groundtruths quantified using kallisto.

In the differential expression analysis, the groundtruths quantified from different data replicates were used for data simulations, forming two pairs of comparisons: DE replicates versus ESC replicates and K562 replicates versus GM12878 replicates. In other analysis, the ESC (H1-hESC cell line) replicate 1 was used as groundtruth. The parameters used for simulation are shown in Supplementary Table 12.

Polyester^90^ software was used to simulate short reads (cDNA-Illumina); 11 datasets of 2 × 150 bp paired-end reads were generated at different sequencing depths: 10, 20, 40, 60, 80, 100, 120, 140, 160, 180 and 200 million read pairs.

IsoSeqSim (https://github.com/yunhaowang/IsoSeqSim) was used to simulate PacBio circular consensus sequencing reads. The tool truncates gene isoform sequences based on given probabilities and introduces uniform sequencing errors. Nine sets of cDNA-PacBio circular consensus sequencing reads are generated at varying sequencing depths: 0.5, 1, 2, 3, 4, 5, 10, 20 and 30 million.

Although NanoSim^91^ has been used actively for long reads simulation studies, it should be noted that it tends to overtruncate the simulated reads on both 5′ and 3′ ends compared with real ONT data. This overtruncation leads to shorter read length, ignoring the 3′-end bias of long reads, such as in cDNA-ONT data (Supplementary Fig. 6).

To produce more realistic simulation data, we developed miniSim (https://github.com/Tidesun/MiniSim), utilizing kernel density estimation (KDE) models from real ONT data to estimate truncation read length. KDE models based on dRNA-ONT and cDNA-ONT from real data were used to address sample and protocol differences. Users can access pretrained generic models or train custom ones with the kde_estimate.py script. This additional kernel density model is not applied in the miniQuant method, thus enabling a fair comparison between miniQuant and existing tools. Error-profile models are built using the same approach as NanoSim. With miniSim, 22 sets of cDNA-ONT and dRNA-ONT reads are generated at varying sequencing depths: 0.5, 1, 2, 3, 4, 5, 10, 15, 20, 25 and 30 million. Unless otherwise specified, cDNA-ONT data were used as long reads in this study.

Simulation data used to train the gradient boosting model have groundtruth based on ESC replicate 1. Combinations of different short- and long-read sequencing depths (10, 20, 40 and 100 million for short reads and 1, 5, 10 and 20 million for long reads) were used to create pretrained models on different data scenarios. An additional replicate was simulated for training the model. See Supplementary Note 13 for additional simulation datasets used in specific analyses.

### Performance evaluation

For the evaluation of quantification performances, all isoform groundtruth and estimated TPMs less than 0.01 are truncated to 0, and only the genes and isoforms with true abundance TPM ≥ 0.01 in groundtruth are considered in the evaluation, which is the same as in Salmon^19^ (Supplementary Note 14). For quantification using sample-specific annotations, the set of evaluated genes and isoforms is further restricted to the genes and isoforms included in the sample-specific annotations. Genes with K-values equal to NA (Supplementary Note 2) are also excluded. For ERCC and SIRV transcripts, their quantification outputs are scaled to have a sum of 10^6^, respectively, before evaluation.

At the gene isoform level, similar to previous studies^18,19^, ARD is used to evaluate quantification accuracy for each gene isoform *t*, calculated as

$${{\rm{ARD}}}_{t}=\left\{\begin{array}{c}\frac{\left|{\theta }_{t}-{\hat{\theta }}_{t}\right|}{{\theta }_{t}+{\hat{\theta }}_{t}},\,{\theta }_{t}\ne 0\,{\rm{or}}\,{\hat{\theta }}_{t}\ne 0,\\ \, 0,\,{\theta }_{t}={\hat{\theta }}_{t}=0,\end{array}\right.$$

where *θ*_*t*_ and ${\hat{\theta }}_{t}$ are the true and estimated abundances. The upper bound of ARD is 1, and ARD is 0 when the estimated value perfectly matches the true value. The ‘median ARD’ in this study is the median across isoforms in the same dataset or the same group of isoforms unless otherwise specified.

At the gene level, considering a gene with *T* isoforms and abundance ${\mathbf{\uptheta} }={({\theta }_{1},{\theta }_{2},\cdots ,{\theta }_{T})}^{{\prime} }$, the MARD and RE metrics are used to evaluate the overall quantification accuracy across all *T* gene isoforms, which can be calculated as

$${\rm{MARD}}=\frac{1}{T}\mathop{\sum }\limits_{t=1}^{T}{{\rm{ARD}}}_{t},$$

$$\rm{RE}=\frac{{\Vert {\mathbf{\uptheta} }-\widehat{{\mathbf{\uptheta}} }\Vert }_{2}}{{\Vert {\mathbf{\uptheta} }\Vert }_{2}}.$$

MARD is adopted primarily in this study given its robustness: MARD ranges from 0 to 1, while RE is between 0 and +∞. MARD is also robust to outliers with extremely small abundance. MARD values of different genes could be further summarized for dataset-level evaluations. The ‘median MARD’ in this study is the median across genes in the same dataset or the same group of genes unless otherwise specified, while the ‘average median MARD’ in this study is average across datasets with different sequencing depths unless otherwise specified. Similarly, the ‘median RE’ in this study is median across genes.

Additionally, for real data with multiple replicates, the true relative abundances **θ** are unknown. In such cases, the average abundance among multiple replicates serves as the groundtruth for calculating MARD.

Irreproducibility is used to evaluate at the gene level the overall variation among multiple replicates across gene isoforms, which is calculated as

$${\rm{Irreproducibility}}=\frac{1}{T}\mathop{\sum }\limits_{t=1}^{T}\frac{{{\rm{s.d.}}}_{t}}{{u}_{t}}$$

where s.d._*t*_ and *u*_*t*_ are the sample s.d. and sample mean of the estimated abundance of gene isoform *t* across multiple replicates. The ‘median irreproducibility’ in this study is median across genes.

When analyzing data consortia, the median of median MARD (irreproducibility) is calculated as the median across cell lines (tissues, or cancer types) of the median MARD (irreproducibility).

For differential expression analysis, the fold changes of gene isoform *t* are calculated using edgeR^39–41^ (v.3.40.2, parameters shown in Supplementary Table 12). True fold changes FC_*t*_ are calculated based on groundtruths, while estimated fold changes ${\overline{{\rm{FC}}}}_{t}$ are calculated based on quantification results. Only isoforms with CPM greater than or equal to 1 in at least two data replicates are included in the differential expression analysis. The edgeR method requires count input. However, the StringTie methods provide only TPM outputs. To overcome this discrepancy, the count information is extracted using a script available in the StringTie manual (https://ccb.jhu.edu/software/stringtie/dl/prepDE.py). To determine the fold changes in the groundtruth, the isoform groundtruths are transformed into expected counts ${N}_{t}^{(E)}=\frac{{\theta }_{t}{\widetilde{l}}_{t}}{{\sum }_{i=1}^{T}{\theta }_{t}{\widetilde{l}}_{t}}\times N$.

Next, gene isoforms with mean groundtruth TPM ≥ 0.01 in at least one of the two conditions are categorized into positive (truly differentially expressed) $${\mathbb{T}}{\mathbb{=}}\left\{{t|}\left|{{\log }_{2}{\rm{FC}}}_{t}\right|\ge 1\,{\rm{and}}\; {\rm{adjusted}}\; {\textit{P}}\;{\rm{value}}\le 0.05\right\}$$ and negative (not truly differentially expressed) $${\mathbb{F}}{\mathbb{=}}\left\{{t|}\left|{{\log }_{2}{\rm{FC}}}_{t}\right|\right.$$$$\left.< 1\,{\rm{or}}\; {\rm{adjusted}}\; {\textit{P}}\;{\rm{value}} > 0.05\right\}$$, where *P* values are adjusted to control the false discovery rate using the Benjamini–Hochberg method^92^.

Based on the estimated fold changes, predicted positives and predicted negatives can also be distinguished with the same thresholds. Then, true positives (TP), true negatives (TN), false positives (FP) and false negatives (FN) can be identified to calculate the precision, recall, accuracy and *F*_1_ score: ${\rm{Precision}}=\frac{{\rm{TP}}}{{\rm{TP}}+{\rm{FP}}}$, ${\rm{Recall}}=\frac{{\rm{TP}}}{{\rm{TP}}+{\rm{FN}}}$, ${\rm{Accuracy}}=\frac{{\rm{TP}}+{\rm{TN}}}{{\rm{TP}}+{\rm{TN}}+{\rm{FP}}+{\rm{FN}}}$, ${F_1}\, {\rm{score}}=2 \times \frac{{\rm{Precision}} \times {\rm{Recall}}}{{\rm{Precision}}+{\rm{Recall}}}$.

### Generation of sequencing data from ESC, DE, PE and PGC

Using the in vitro differentiation systems we have developed recently, we generated DE and PE^59^ and PGC^60^ from human ESC (H1-hESC cell line), respectively. Total RNA was extracted by TRIzol Reagent (Thermo Fisher Scientific, cat. no. 15596026), followed by polyA^+^ RNA enrichment using the Dynabeads mRNA Purification Kit (Thermo Fisher Scientific, cat. no. 61006). ONT sequencing libraries were prepared using the SQK-PCS109 kit for PCR-cDNA sequencing (cDNA-ONT) and the SQK-RNA002 kit for direct RNA-seq (dRNA-ONT). Sequencing was conducted using ONT MiniON/GridION flow cells with R9.4.1 chemistry, with real-time base calling performed by Guppy (v.4.2.3). Only the long reads with qscore ≥ 7 (pass) were used for this study. Paired-end Illumina RNA-seq data (2 × 150 bp) were generated in Novogene company, with three replicates for each of four samples (ESC, DE, PE, PGC) and sequencing adapters were trimmed by Cutadapt (v.1.8.1)^93^ with the parameters ‘-q 20,20 -m 20 --max-n 0.01 --trim-n’. Sequencing data are summarized in Supplementary Table 5. A total of 32.26 million cDNA-ONT reads, 14.55 million dRNA-ONT reads and 328.37 million Illumina read pairs were generated.

### Experimental validation by RT–qPCR

In the RT–qPCR experiments, 50 isoforms from genes with high K-values (K-value > 25) and 50 isoforms from genes with low K-values (K-value < 5) were selected as target isoforms.

For primer design, isoform-specific exons were utilized through an inhouse Python script (https://github.com/Tidesun/RT-qPCR-isoform-selection). Primer pairs that target genomic regions containing introns larger than 1 kb or exon–intron junctions were the top priority. Primers for RT–qPCR are listed in Supplementary Table 14.

After total RNA extraction from ESC and DE samples, cDNA was synthesized using High-Capacity cDNA Reverse Transcription Kit (Applied Biosystems, cat no. 4374966). RT–qPCR experiments were conducted with PowerUp SYBR Green Master Mix (Applied Biosystems, cat no. A25742) and on a QuantStudio 5 Real-Time PCR System. Each isoform from each sample was tested in triplicate. Seven out of the 100 targeted regions (five isoforms from high K-value genes and two low K-value) failed in at least two replicates and were excluded. A potential reason is that the selected target regions may not be suitable for primer design. The fold changes of target isoforms were calculated following $${2}^{-\Delta \Delta {C}_{T}}$$ methods^94^: the fold change of target isoform *i* equals $${2}^{-\left(\left({\bar{C}}_{T,i,{\rm{DE}}}-{\bar{C}}_{T,{\rm{ref}},{\rm{DE}}}\right)-\left({\bar{C}}_{T,i,{\rm{ESC}}}-{\bar{C}}_{T,{\rm{ref}},{\rm{ESC}}}\right)\right)}$$, where $${\bar{C}}_{T,i,{\rm{ESC}}}$$ and $${\bar{C}}_{T,i,{\rm{DE}}}$$ are the mean *C*_*T*_ value of isoform *i* across the three replicates in ESC and DE datasets, respectively, and $${\bar{C}}_{T,{\rm{ref}},{\rm{ESC}}}$$ and $${\bar{C}}_{T,{\rm{ref}},{\rm{DE}}}$$ are the mean *C*_*T*_ value across the three replicates for reference gene DDB1 (ref. ^95^) in ESC and DE datasets, respectively. The *P* values were calculated by performing Student’s *t*-test on $${{\Delta C}_{T,i,{\rm{DE}},j}=C}_{T,i,{\rm{DE}},j}-{\bar{C}}_{T,{\rm{ref}},{\rm{DE}}}$$ and $${{\Delta}{C}_{T,i,{\rm{ESC}},j}}=$$$${C}_{T,i,{\rm{ESC}},j}-{\bar{C}}_{T,{\rm{ref}},{\rm{ESC}}}$$, where *j* = 1,2,3 represents the replicate. The *P* values were then adjusted using the Benjamini–Hochberg method^92^. The fold changes *t* statistics, degree of freedom and *P* values of the qPCR results are shown in Supplementary Table 15. Among the 93 isoforms, 39 were determined as truly differentially expressed and 54 as truly nondifferentially expressed.

The parameters for comparing the qPCR and real short-reads differential analysis results (Extended Data Fig. 4a) were tested with the combinations of log_2_ fold change threshold (0.5, 0.75, 1 and 1.5) and CPM threshold (1, 2, 3, 4 and 5).

### Analyses of isoform switching

For identification of gene isoforms, based on the hg38 reference genome and GENCODE annotation (v.39), IsoQuant (v.3.3.0) was run with the parameter ‘-d nanopore’ for cDNA-ONT data merged from ESC, PE and PGC, and with the parameters ‘-d nanopore --stranded forward’ for dRNA-ONT data merged from ESC, PE and PGC. IsoQuant outputs both known gene isoforms that are included in GENCODE, and new gene isoforms that are not included in GENCODE. For each of three samples (ESC, PE and PGC), two sets of gene annotations were derived using IsoQuant from cDNA-ONT and dRNA-ONT data. To estimate the abundance of these gene isoforms, for each sample, miniQuant-H was run separately with the two sets of gene annotations and the two dataset combinations: (1) cDNA-ONT derived annotation and cDNA-ONT plus Illumina data; (2) dRNA-ONT derived annotation and dRNA-ONT plus Illumina data. Of note, for each sample, the Illumina data were combined from three replicates when running miniQuant-H.

To identify genes with isoform switches during the two differentiation paths, we consider only genes with sufficient expression levels (TPM ≥ 10). Taking the differentiation path (ESC to PE) as an example, the usage ratio (ranging from 0 to 100%) for each isoform within a gene in ESC and PE samples is calculated, respectively. A gene isoform is denoted as a switched isoform if the absolute difference in its usage ratio between ESC and PE is ≥20%. A gene is considered as isoform-switching if it has ≥1 switched isoform. To improve reliability, for a gene to be conclusively considered as undergoing isoform switching, it must demonstrate such changes across the two sets of data: cDNA-ONT plus Illumina and dRNA-ONT plus Illumina.

For the statistics of gene isoforms identified by IsoQuant and quantified by miniQuant-H, we considered only those isoforms with TPM ≥ 1 in both cDNA-ONT plus Illumina data and dRNA-ONT plus Illumina data. New gene isoforms that are independently identified from cDNA-ONT and dRNA-ONT were deemed coidentified if they shared identical splicing junctions. Gene ontology (GO) analysis was carried out using DAVID (v.2021)^96^, with one-sided exact *P* value.

### Reporting summary

Further information on research design is available in the Nature Portfolio Reporting Summary linked to this article.

## Online content

Any methods, additional references, Nature Portfolio reporting summaries, source data, extended data, supplementary information, acknowledgements, peer review information; details of author contributions and competing interests; and statements of data and code availability are available at 10.1038/s41587-025-02633-9.

## Supplementary information

Supplementary Information Supplementary Figs. 1–6 and Notes 1–14.

Reporting Summary

Supplementary Tables 1–15 Supplementary Tables 1–15.

Supplementary Code 1 Supplementary code to reproduce the results. The folder ‘Figure_visualization’ stores the code to reproduce the figures. The folder ‘MetricCalculation’ stores the code to calculate the evaluation metrics used in the results. The folder ‘miniQuant’ stores the software of miniQuant to reproduce the quantification results.

## Data Availability

Short-read datasets from three consortia, GTEx^24,25^, TCGA^25,26^ and ENCODE^27^ and their corresponding accession numbers and download links are introduced in Supplementary Tables 1–3. Short-read and long-read datasets with synthetic ERCC and SIRV spike-in transcripts from the LRGASP consortium^13^ and their corresponding accession numbers are introduced in Supplementary Table 4. The sequencing data of ESC, DE, PE and PGC generated in this study are available at the Gene Expression Omnibus (GEO) under the accession number GSE265988 and are described in Supplementary Table 5. The short-read RNA-seq datasets^88,89^ used for generating simulation datasets and their corresponding accession numbers and download links are introduced in Supplementary Table 13.

## Code availability

MiniQuant is available at https://github.com/Augroup/miniQuant. The Supplementary Code to reproduce the result is available via Zenodo at 10.5281/zenodo.14889304 (ref. ^28^). MiniSim and pretrained KDE models are available at https://github.com/Tidesun/MiniSim.
