# Supplementary material for: Improving gene isoform quantification with miniQuant
Source: Nat Biotechnol. 2025 Jun 3;44(3):477–89. doi: 10.1038/s41587-025-02633-9 (PMC12346831; doi:10.1038/s41587-025-02633-9)
Supplement: Supplementary file 1 — Supplementary Figs. 1–6 and Notes 1–14. [file 41587_2025_2633_MOESM1_ESM.pdf]

nature biotechnology

Supplementary information https://doi.org/10.1038/s41587-025-02633-9

# Improving gene isoform quantification with miniQuant

In the format provided by the authors and unedited

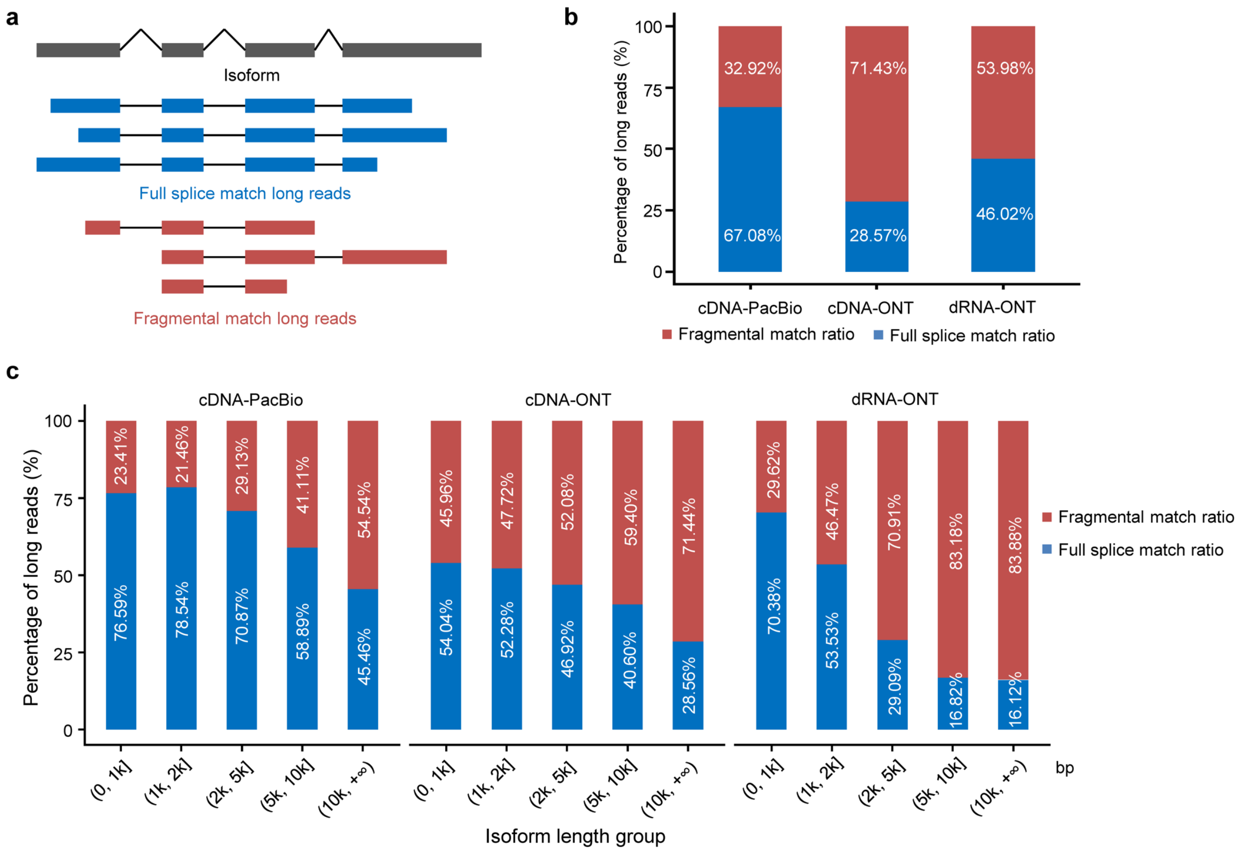


**Supplementary Fig. 1 | Overview of long reads evaluated from SQANTI3.**

**a,** Schematic illustration of the full splice match (FSM) and fragmental match (FM) long reads from SQANTI3.

**b,** Percentages of FM and FSM long reads in three different protocols. The proportions are computed using SQANTI3 on ESC rep1 from the LRGASP consortium.

**c**, Percentages of FM and FSM long reads among different isoform groups. The proportions are computed using SQANTI3 on ESC rep1 from the LRGASP consortium.

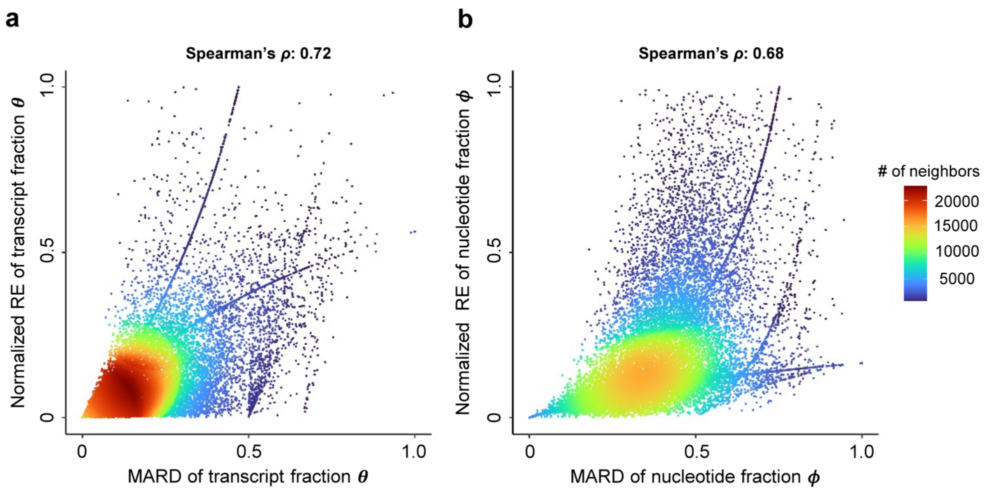


**Supplementary Fig. 2 | The correlation between two quantification error metrics based on nucleotide and transcript fractions.**

**a-b,** The two-dimensional density plot represents the correlation between mean absolute relative difference (MARD) and relative error (RE) calculated based on **a**, transcript fractions and **b**, nucleotide fractions. The kallisto is employed to quantify simulation data of 40 million 2x150 bp short read pairs. Genes with the top 1% RE are considered outliers and are removed, and RE values are then scaled to the range from 0 to 1.

**a**

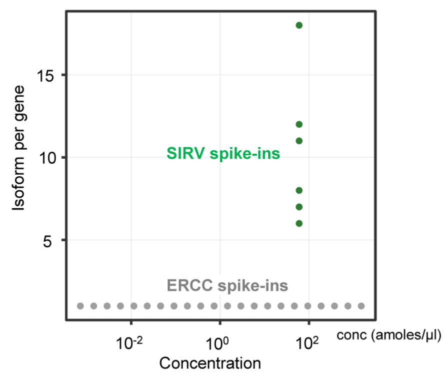


**b**

| Gene loci | Number of isoforms | K-value |
| --- | --- | --- |
| SIRV1 | 8 | 6.23 |
| SIRV2 | 6 | 15.04 |
| SIRV3 | 11 | 15.80 |
| SIRV4 | 7 | 5.73 |
| SIRV5 | 12 | 20.50 |
| SIRV6 | 18 | 16.12 |
| SIRV7 | 7 | 24.20 |

**c**

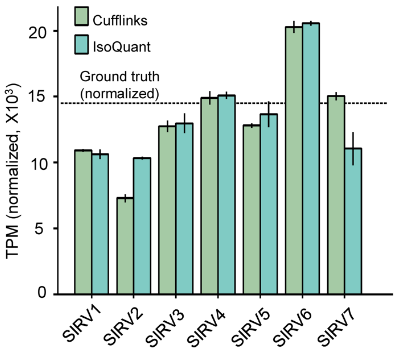


**Supplementary Fig. 3 | The ERCC and SIRV spike-ins.**

**a,** Concentrations and complexity of 69 SIRV (corresponding to 7 gene loci) and 92 ERCC spike-in transcripts in SIRV-Set 4. Data is obtained from https://www.lexogen.com/wp-content/uploads/2021/06/SIRV_Set4_Norm_sequence-design-overview_20210507a.xlsx.

**b,** Isoform numbers and K-values of SIRV genes. The SIRV set4 spike-in genomic sequences and the annotations in GTF format are available from https://lrgasp.github.io/lrgasp-submissions/docs/reference-genomes.html.

**c**, Comparison of the TPM between Cufflinks and IsoQuant on 7 SIRV gene loci. The average values and standard deviations of TPM among samples ESC and DE are estimated for visualization (n=6 biological replicates). In bar plots, data are presented as mean values +/- standard error.

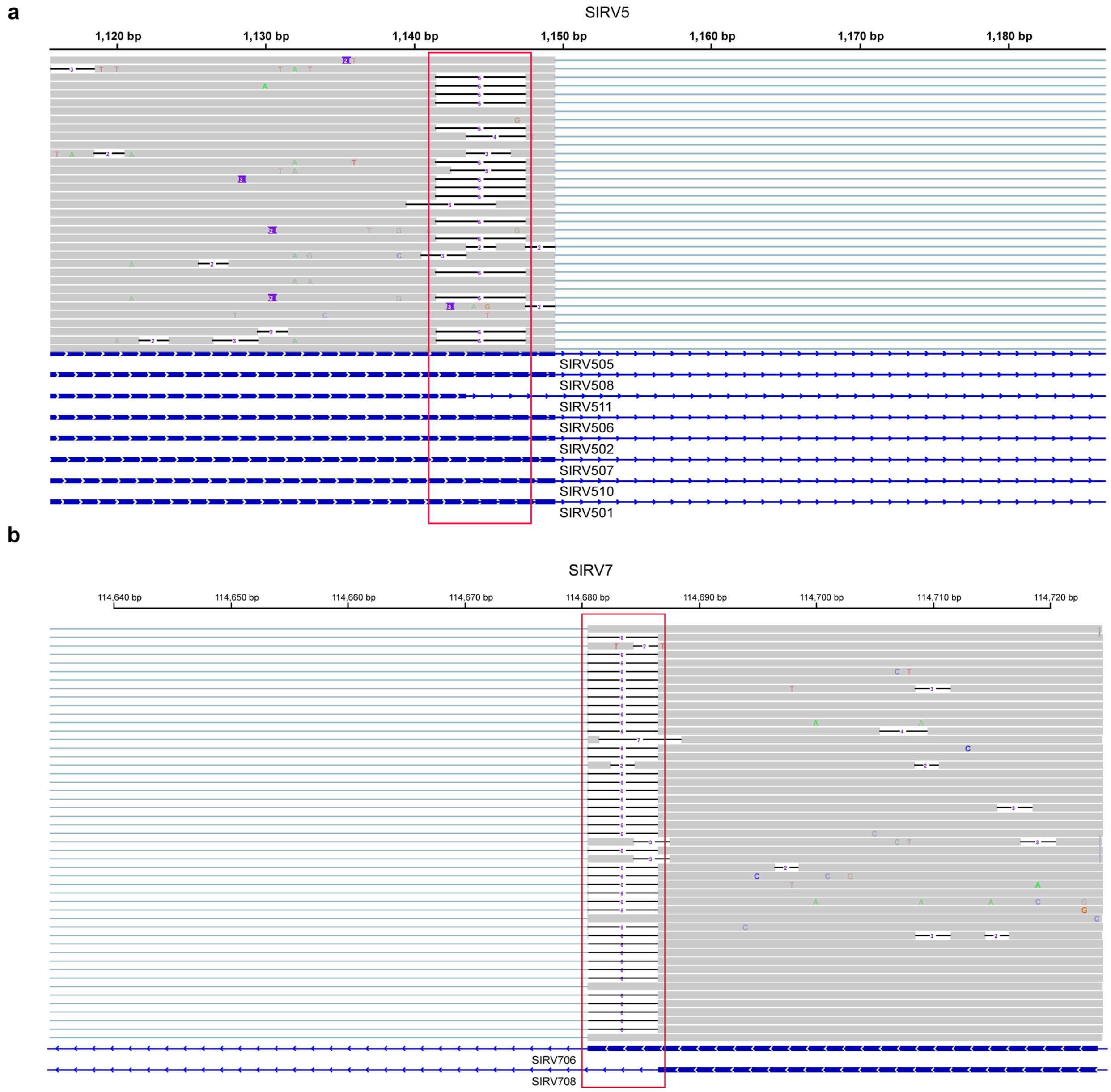


**Supplementary Fig. 4 |** Reads alignment tracts of (**a**) SIRV5 and (**b**) SIRV7 in the ESC sample. Regions with error mapping are marked with red boxes.

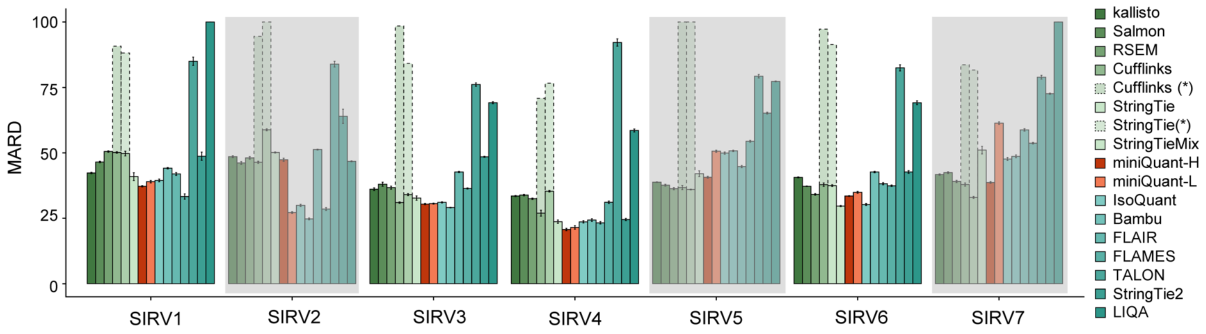


**Supplementary Fig. 5 |** Comparison of the MARD among 15 tools on 7 SIRV gene loci. The average values and standard deviations of MARD among samples ESC and DE are estimated for visualization (n=6 biological replicates). *, de novo sample-specific annotation identified by Cufflinks and StringTie. In bar plots, data are presented as mean values +/- standard error.

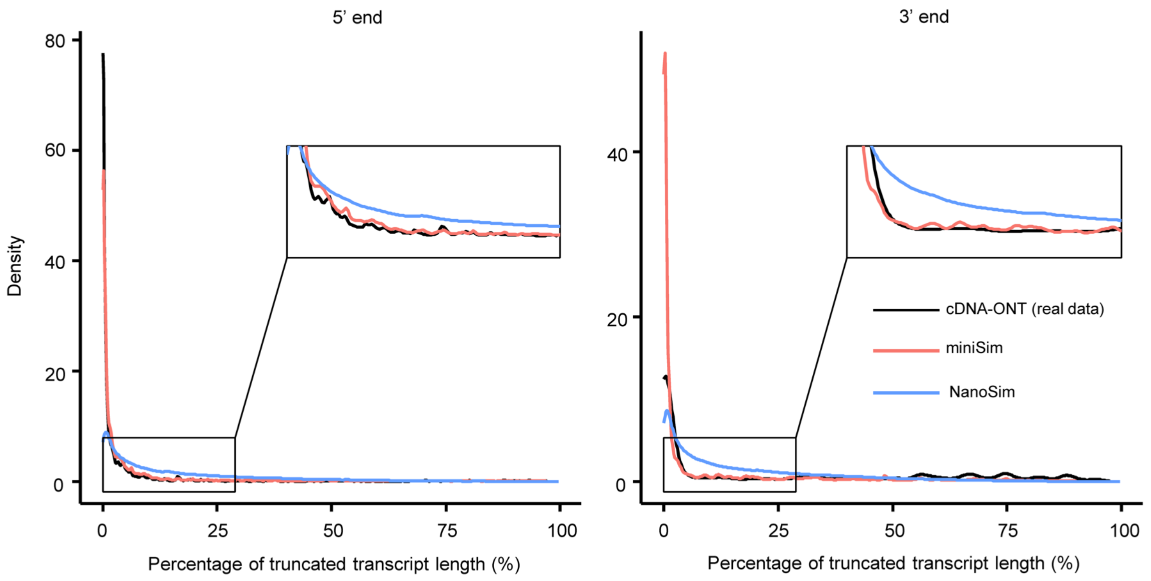


**Supplementary Fig. 6 |** The empirical distribution of truncated read lengths between real data (red, cDNA-ONT on ESC) and simulated data (simulated with miniSim and NanoSim).

**Supplementary Notes 1: Theorical re-examination of previous short read-based methods under generalized linear model framework and the construction of conditional probability matrix for K-value.**

In this note, current short read-based methods are adapted into a generalized linear model (GLM) framework, and the structure of the read-isoform alignment conditional probability matrix $\boldsymbol{A}$ (also see Supplementary Notes 2) utilized in quantification error analysis and K-value calculation is demonstrated. (See Supplementary Notes 4 for the model of miniQuant.)

## 1.1. Current methods

For simplicity, consider a basic model where only the gene isoforms of origin and the start sites of reads are taken into account. Without loss of generality, consider a gene consisting of $T$ expressed gene isoforms with relative abundances $\boldsymbol{\theta} = (\theta_1, \theta_2, \cdots, \theta_T)'$. Assume $N$ independent reads $R_1, R_2, \cdots, R_N$ with the same read length $L$ are generated from the gene, and the start site of each read is uniformly distributed over its gene isoform of origin. The likelihood functions of read-based models and region-based models[1] are presented below.

- **1.1.1. Read-based models**.

When the $N$ reads are from a short-read sequencing platform, the probability of $R_n, 1 \leq n \leq N$ originating from gene isoform $t$ is given by

$$\phi_t = P(R_n \in t) = \frac{\theta_t \tilde{l}_t}{\sum_{j=1}^{T} \theta_j \tilde{l}_j},$$

where $\tilde{l}_t = l_t - L + 1$ is the effective length of gene isoform $t$, with $l_t$ being the length of gene isoform $t$. Under the uniform start site distribution, the probability of observing $R_n$ is

calculated as

$$P(\text{observing } R_n) = \sum_{t=1}^{T} P(\text{observing } R_n | R_n \in t) P(R_n \in t)$$

$$= \sum_{t=1}^{T} \frac{1}{\tilde{l}_t} \cdot 1\{R_n \text{ maps to gene isoform } t\} \cdot \phi_t \,, \tag{S1}$$

where $1\{R_n \text{ maps to gene isoform } t\}$ is the indicator function that read $R_n$ can be mapped to gene isoform $t$. Then, the likelihood function of the read-based model is

$$L_1(\boldsymbol{\phi}) = \prod_{n=1}^{N} \sum_{t=1}^{T} \frac{1}{\tilde{l}_t} \cdot 1\{R_n \text{ maps to gene isoform } t\} \cdot \phi_t \,. \tag{S2}$$

- **1.1.2. Region-based models**

Instead of directly modeling the probability of observing each read, region-based models split the gene into disjoint regions and summarize all reads into region read counts, which are then modeled with certain distributions. Assume the gene consists of $S$ disjoint regions[2] (Supplementary Notes 2), and the read count in region $s$ is $N_s, 1 \le s \le S$. $(N_1, N_2 \cdots, N_S)'$ follows a multinomial distribution, with probability parameters $p_s, 1 \le s \le S$. $p_s$'s are the probability that a randomly selected read $R_n$ falls into region $s$, formulated as

$$p_s = P(\, R_n \in s) = \sum_{t=1}^{T} P(R_n \in s | R_n \in t) P(R_n \in t) = \sum_{t=1}^{T} \frac{\tilde{r}_s}{\tilde{l}_t} \cdot 1\{s \in t\} \cdot \phi_t \,,$$

where $1\{s \in t\}$ is the indicator function that region $s$ is contained in gene isoform $t$, and $\tilde{r}_s$ is the effective length of region $s$ (Supplementary Notes 2). The likelihood function of the multinomial model is

$$L_2(\boldsymbol{\phi}) = \prod_{s=1}^{S}\left(\sum_{t=1}^{T}\frac{\tilde{r}_s}{\tilde{l}_t}\cdot 1\{s \in t\}\cdot \phi_t\right)^{N_s}. \tag{S3}$$

Since $N$ is usually very large and $p_s$'s are very small, the region read counts can be approximated by a Poisson distribution[2-5]. The likelihood function of the basic Poisson model with mean parameter $\mu_s = Np_s, 1 \le s \le S$ is

$$L_3(\boldsymbol{\phi}) = \prod_{s=1}^{S}\frac{e^{-\mu_s}\mu_s^{N_s}}{N_s!}$$

$$= \prod_{s=1}^{S}\frac{\exp\left\{-N\sum_{t=1}^{T}\frac{\tilde{r}_s}{\tilde{l}_t}\cdot 1\{s \in t\}\cdot \phi_t\right\}\cdot\left(N\sum_{t=1}^{T}\frac{\tilde{r}_s}{\tilde{l}_t}\cdot 1\{s \in t\}\cdot \phi_t\right)^{N_s}}{N_s!}. \tag{S4}$$

Another approximation utilizes normal distribution[6-9]. Assume region read proportions $b_s = \frac{N_s}{N}, 1 \le s \le S$ have the same variance $\tau^2$, then $b_s \sim N(p_s, \tau^2) 1 \le s \le S$, and the likelihood function of the normal model is:

$$L_4(\boldsymbol{\phi}) = \prod_{s=1}^{S}\frac{1}{\tau\sqrt{2\pi}}\exp\left\{-\frac{(b_s - p_s)^2}{2\tau^2}\right\}$$

$$= \prod_{s=1}^{S}\frac{1}{\tau\sqrt{2\pi}}\exp\left\{-\frac{1}{2\tau^2}\left(b_s - \sum_{t=1}^{T}\frac{\tilde{r}_s}{\tilde{l}_t}\cdot 1\{s \in t\}\cdot \phi_t\right)^2\right\}. \tag{S5}$$

- **1.1.3. Model equivalence in GLM framework**

Next, the equivalence between Eqs. (S2), (S3), (S4), and (S5) is shown in a GLM framework.

The disjoint regions involved in region-based models are partitioned so that reads falling into the same region map to the same set of gene isoforms. Denote the reads in region $s$ as $R_j^s, 1 \le j \le N_s, 1 \le s \le S$, and the set of gene isoforms to which these reads are

mapped as $\mathcal{T}^s, 1 \le s \le S$. By Eq. (S1), $P(\text{observing } R_j^s), 1 \le j \le N_s$ are equal because they have the same potential gene isoforms of origin $\mathcal{T}^s$. Consequently, reads belonging to the same region can be grouped in Eq. (S2), which can be further simplified as

$$L_1(\boldsymbol{\phi}) = \prod_{n=1}^{N} \sum_{t=1}^{T} \frac{1}{\tilde{l}_t} \cdot 1\{R_n \text{ maps to gene isoform } t\} \cdot \phi_t$$

$$= \prod_{s=1}^{S} \left( \sum_{t=1}^{T} \frac{1}{\tilde{l}_t} \cdot 1\{s \in t\} \cdot \phi_t \right)^{N_s} \propto L_2(\boldsymbol{\phi}). \tag{S6}$$

Therefore, the multinomial model (Eq. (S3)) is equivalent to the read-based model (Eq. (S2)), as their likelihood functions are equal up to a constant and thus result in the same maximum likelihood estimator (MLE) of $\boldsymbol{\phi}$. In addition, equivalence between the read-based model and the Poisson model has been proved[10], which induces the equivalence among the three models (Eqs. (S2), (S3) and (S4)), leading to the same MLEs.

Although the likelihood functions of multinomial, Poisson, and normal models (Eqs. (S3), (S4) and (S5)) look very different, the mean parameters of their observed variables can be expressed in GLM format. Specifically, the normal model constructs the relationship between region read proportions and fractions of nucleotides by

$$E[b_s] = \sum_{t=1}^{T} \frac{\tilde{r}_s}{\tilde{l}_t} \cdot 1\{s \in t\} \cdot \phi_t, 1 \le s \le S; \tag{S7}$$

multinomial and Poisson models, instead, construct the relationship between region read counts and fractions of nucleotides by

$$E[N_s] = N \left( \sum_{t=1}^{T} \frac{\tilde{r}_s}{\tilde{l}_t} \cdot 1\{s \in t\} \cdot \phi_t \right), 1 \le s \le S, \tag{S8}$$

which is mathematically equivalent to Eq. (S7) if both sides are divided by $N$. Both Eq.

(S7) and Eq. (S8) are in a GLM format with the identity link function.

In terms of estimation, GLMs estimate $\boldsymbol{\phi}$ by maximizing likelihood functions. The MLE of the normal model (Eq. (S5)) approximates the MLEs of the read-based model, the multinomial model as well as the Poisson model[10].

**1.2. Read-isoform alignment conditional probability matrix**

Quantification error properties are derived based on the normal model. In matrix notation, finding the MLE for the normal model (i.e., linear model, Eq. (S7)) is equivalent to solving the least squares problem

$$\widehat{\boldsymbol{\phi}} = \underset{\boldsymbol{\phi}}{\operatorname{argmin}} \|\boldsymbol{b} - \boldsymbol{A}\boldsymbol{\phi}\|_2^2 , \tag{S9}$$

where $\boldsymbol{b} = (b_1, b_2, \cdots, b_S)'$ are the read proportions for regions, $\boldsymbol{A} = (A_{st}) \in \mathbb{R}^{S \times T}$ is the read-isoform alignment conditional probability matrix with $A_{st} = P(R_n \in s | R_n \in t) = \frac{\tilde{r}_s}{\tilde{l}_t} \cdot 1\{s \in t\}$. Eq. (S9) is also equivalent to finding the least square solution of the linear system

$$\boldsymbol{b} = \boldsymbol{A}\boldsymbol{\phi} \tag{S10}$$

for $\boldsymbol{\phi}$. This linear system is the basis of the definition of K-value.

**Remark S1.** Most read-based methods enhance the basic model (Eq. (S2)) by accounting for additional sequencing information. For example, RSEM[11] considers fragment lengths and orientations of paired-end reads, and Salmon[12] integrates GC content into the model.

**Remark S2.** When computing the likelihood function, read-based methods often organize reads into equivalence classes to improve the computation efficiency. In the basic model, equivalence classes coincide with regions, because reads in the same region have the same individual contribution to the likelihood function in Eq. (S2). More complex read-based models may define equivalence classes in different ways[4,12,13]. Nevertheless, it has been shown that read counts in equivalence classes are sufficient statistics for $\boldsymbol{\phi}$[4].

**Remark S3.** Existing methods also differ in their formulation of $\boldsymbol{A}$ due to the diverse sequencing information and different equivalence classes they incorporate. In this note, the most fundamental model without software-specific details is adopted to derive common error properties for gene isoform quantification.

**Remark S4.** The analysis of quantification errors induced by long-read sequencing data aligns with this framework, with two major modifications: 1) the probability that a randomly selected read is from gene isoform $t$ is $\theta_t$ instead of $\phi_t$, because long-read sequencing does not involve fragmentation and thus the number of reads generated from gene isoform $t$ is no longer related to its length; 2) the format of $\boldsymbol{A}$ is different as the lengths of long reads are gene isoform-specific.

**Supplementary Notes 2: Definition of gene regions and the special cases in K-value calculation.**

In this note, the definition of gene regions is introduced and the special cases in K-value calculation are discussed.

Based on the transcript annotation, a gene is partitioned into fragments with segmentations at exon-exon junction points and start/end points of all its isoforms. Then, given the read length, for each potential read start site (or possible position of read alignment), the fragments to which the read is aligned are identified. A single read may be aligned to more than one fragment, and read start sites aligned to the same combination of fragments form a region. The effective length $\tilde{r}_s$ of region $s$ is the count of read start sites aligned to the combination of fragments corresponding to region $s$. Therefore, the matrix $\boldsymbol{A} \in \mathbb{R}^{S \times T}$ is a region-by-isoform matrix, with regions defined by read-isoform alignment. Given that many genes are not annotated with isoforms that have start/end/junction points different from the gene start/end points and the exon-exon junction points, the term "exon-isoform structure" is also used for matrix $\boldsymbol{A}$ for simplicity.

In this study, K-values are calculated based on 150 bp read length. However, since the matrix $\boldsymbol{A}$ is created based on read-isoform alignments, isoforms shorter than the read length cannot be aligned. Genes for which all the annotated isoforms are less than or equal to 150 bp in length have K-values undefined (equal to NA). A total of 698 genes fall into this category when K-values are calculated using GENCODE annotation (version 39) and 150 bp read length.

99.91% of the 29,257 genes with K-value 1 have a single isoform. There are some special cases where 27 genes with multiple isoforms have K-values equal to 1. 19 genes have

only one of the annotated isoforms greater than 150 bp. In such case the K-values of such genes are equal to 1. For two of the genes, distinct isoforms result in identity matrix $\boldsymbol{A}$, thus K-value 1. Additionally, miniQuant allows for one base of error when determining regions. Six of the genes have two nearly identical annotated isoforms that differ by only a single base, leading to matrix $\boldsymbol{A}$ with identical columns and K-value 1.

**Supplementary Notes 3: Relationship between quantification error and K-value.**

In this note, the relationship between quantification error and K-value is derived for both full-column-rank and non-full-column rank matrix $\boldsymbol{A}$.

## 3.1. Quantification error for full-column-rank genes

In this section, the sensitivity of the least squares solution (LSS) $\widehat{\boldsymbol{\phi}}$ of Eq. (S10) to minor perturbations in $\boldsymbol{A}$ and $\boldsymbol{b}$ is explored. Assume $\boldsymbol{A} = (A_{st}) \epsilon \mathbb{R}^{S \times T}$ is a non-square matrix with full column rank, i.e., $\mathrm{rank}(\boldsymbol{A}) = T$. Theoretical analysis is conducted in the following three cases: (i) only a perturbation in $\boldsymbol{b}$; (ii) only a perturbation in $A$; (iii) perturbations in both $A$ and $b$. Given the asymptotic normality of MLEs for GLMs[14], the sensitivity of $\widehat{\boldsymbol{\phi}}$ in GLMs would be similar to that in a linear model. Thus, the following derivations are based on the linear model Eq. (S10).

- **3.1.1. Sensitivity to only a perturbation in $\boldsymbol{b}$**

In real-world scenarios, the observed $\boldsymbol{b} \in \mathbb{R}^S$ of $S$ regions are often inaccurate due to subtle perturbations arising from variations in sequencing depths. In this subsection, the magnitude of quantification error is explored when only $\boldsymbol{b}$ is perturbed.

**Theorem S1.** Assume $\boldsymbol{b}$ is perturbed to $\widehat{\boldsymbol{b}} = \boldsymbol{b} + \delta \boldsymbol{b}$ in linear system Eq. (S10)*.* Denote the LSS of Eq. (S10) as $\boldsymbol{\phi}$, and the solution to the perturbed least squares problem (LSP) of Eq. (S10) by $\widehat{\boldsymbol{\phi}} = \boldsymbol{\phi} + \delta\boldsymbol{\phi}$. Then,

$$\frac{\|\delta\boldsymbol{\phi}\|_2}{\|\boldsymbol{\phi}\|_2} \leq \sec\rho \cdot K(\boldsymbol{A}) \cdot \frac{\|\delta\boldsymbol{b}\|_2}{\|\boldsymbol{b}\|_2}, \quad \text{(S11)}$$

where $\sec\rho = \frac{\|\boldsymbol{b}\|_2}{\|\boldsymbol{A\phi}\|_2}$, $\rho$ is the angle between $\boldsymbol{b}$ and $\boldsymbol{A\phi}$*.*

**Proof.** Rearranging the terms in

$$A(\boldsymbol{\phi} + \delta\boldsymbol{\phi}) = \boldsymbol{b} + \delta\boldsymbol{b},$$

we have

$$\boldsymbol{A}\delta\boldsymbol{\phi} = \boldsymbol{b} - \boldsymbol{A}\boldsymbol{\phi} + \delta\boldsymbol{b}. \tag{S12}$$

Multiply both side with $(\boldsymbol{A}'\boldsymbol{A})^{-1}\boldsymbol{A}'$,

$$\delta\boldsymbol{\phi} = (\boldsymbol{A}'\boldsymbol{A})^{-1}\boldsymbol{A}'(\boldsymbol{b} - \boldsymbol{A}\boldsymbol{\phi}) + (\boldsymbol{A}'\boldsymbol{A})^{-1}\boldsymbol{A}'\delta\boldsymbol{b}.$$

Given that

$$(\boldsymbol{A}'\boldsymbol{A})^{-1}\boldsymbol{A}'\,(\boldsymbol{b} - \boldsymbol{A}\boldsymbol{\phi}) = (\boldsymbol{A}'\boldsymbol{A})^{-1}\boldsymbol{A}'\boldsymbol{b} - (\boldsymbol{A}'\boldsymbol{A})^{-1}\boldsymbol{A}'\,\boldsymbol{A}\boldsymbol{\phi} = \boldsymbol{\phi} - \boldsymbol{\phi} = \boldsymbol{0},$$

we have

$$\delta\boldsymbol{\phi} = (\boldsymbol{A}'\boldsymbol{A})^{-1}\boldsymbol{A}'\delta\boldsymbol{b}.$$

Let $\boldsymbol{A}^{+} = (\boldsymbol{A}'\boldsymbol{A})^{-1}\boldsymbol{A}'$, which is the Moore-Penrose inverse of $\boldsymbol{A}$.

According to the properties of matrix norm, we have

$$\|\delta\boldsymbol{\phi}\|_2 \leq \|\boldsymbol{A}^{+}\|_2 \cdot \|\delta\boldsymbol{b}\|_2. \tag{S13}$$

For matrix $\boldsymbol{A}^{+}$, we have $(\boldsymbol{A}^{+})' = \boldsymbol{A}(\boldsymbol{A}'\boldsymbol{A})^{-1}$ and

$$\boldsymbol{A}^{+}(\boldsymbol{A}^{+})' = (\boldsymbol{A}'\boldsymbol{A})^{-1}\boldsymbol{A}'\boldsymbol{A}(\boldsymbol{A}'\boldsymbol{A})^{-1} = (\boldsymbol{A}'\boldsymbol{A})^{-1}.$$

Hence,

$$\|\boldsymbol{A}^{+}\|_2^2 \cdot \|\boldsymbol{A}\|_2^2 = \|\boldsymbol{A}^{+}(\boldsymbol{A}^{+})'\|_2 \cdot \|\boldsymbol{A}'\boldsymbol{A}\|_2 = \|(\boldsymbol{A}'\boldsymbol{A})^{-1}\|_2 \cdot \|\boldsymbol{A}'\boldsymbol{A}\|_2 = K^2(\boldsymbol{A}),$$

i.e.,

$$\|\boldsymbol{A}^{+}\|_2 \cdot \|\boldsymbol{A}\|_2 = K(\boldsymbol{A}).$$

Therefore, combing with Eq. (S13), we obtain

$$\begin{aligned}\frac{\|\delta\boldsymbol{\phi}\|_2}{\|\boldsymbol{\phi}\|_2} &\leq \|\boldsymbol{A}^{+}\|_2 \cdot \frac{\|\delta\boldsymbol{b}\|_2}{\|\boldsymbol{\phi}\|_2} \\ &= \|\boldsymbol{A}^{+}\|_2 \cdot \|\boldsymbol{A}\|_2 \cdot \frac{\|\boldsymbol{b}\|_2}{\|\boldsymbol{A}\|_2 \cdot \|\boldsymbol{\phi}\|_2} \cdot \frac{\|\delta\boldsymbol{b}\|_2}{\|\boldsymbol{b}\|_2} \\ &= K(\boldsymbol{A}) \cdot \frac{\|\boldsymbol{b}\|_2}{\|\boldsymbol{A}\|_2 \cdot \|\boldsymbol{\phi}\|_2} \cdot \frac{\|\delta\boldsymbol{b}\|_2}{\|\boldsymbol{b}\|_2}. \end{aligned} \tag{S14}$$

Since

$$\frac{\|\boldsymbol{b}\|_2}{\|\boldsymbol{A}\|_2\cdot\|\boldsymbol{\phi}\|_2}\le\frac{\|\boldsymbol{b}\|_2}{\|\boldsymbol{A\phi}\|_2}=\sec\rho,$$

combing with Eq. (S14), we get

$$\frac{\|\delta\boldsymbol{\phi}\|_2}{\|\boldsymbol{\phi}\|_2}\le\sec\rho\cdot K(\boldsymbol{A})\cdot\frac{\|\delta\boldsymbol{b}\|_2}{\|\boldsymbol{b}\|_2}.\ \blacksquare$$

**Theorem S2.** Assume $\boldsymbol{b}$ is perturbed to $\widehat{\boldsymbol{b}}=\boldsymbol{b}+\delta\boldsymbol{b}$ in linear system Eq. (S10). Denote the LSS of Eq. (S10) as $\boldsymbol{\phi}$, and the solution to the perturbed LSP of Eq. (S10) as $\widehat{\boldsymbol{\phi}}=\boldsymbol{\phi}+\delta\boldsymbol{\phi}$. Let $\boldsymbol{A}=\boldsymbol{U\Sigma V}'$ be the singular value decomposition (SVD) of $\boldsymbol{A}$, then,

$$\delta\boldsymbol{\phi}=\sum_{i=1}^{T}\frac{\boldsymbol{u}_i'\delta\boldsymbol{b}}{\sigma_i}\boldsymbol{v}_i=\begin{bmatrix}\frac{\boldsymbol{u}_1'\delta\boldsymbol{b}}{\sigma_1}v_{11}+\frac{\boldsymbol{u}_2'\delta\boldsymbol{b}}{\sigma_2}v_{21}+\cdots+\frac{\boldsymbol{u}_T'\delta\boldsymbol{b}}{\sigma_T}v_{T1}\\ \frac{\boldsymbol{u}_1'\delta\boldsymbol{b}}{\sigma_1}v_{12}+\frac{\boldsymbol{u}_2'\delta\boldsymbol{b}}{\sigma_2}v_{22}+\cdots+\frac{\boldsymbol{u}_T'\delta\boldsymbol{b}}{\sigma_T}v_{T2}\\ \vdots\\ \frac{\boldsymbol{u}_1'\delta\boldsymbol{b}}{\sigma_1}v_{1T}+\frac{\boldsymbol{u}_2'\delta\boldsymbol{b}}{\sigma_2}v_{2T}+\cdots+\frac{\boldsymbol{u}_T'\delta\boldsymbol{b}}{\sigma_T}v_{TT}\end{bmatrix},\tag{S15}$$

where

$\boldsymbol{\Sigma}=\begin{bmatrix}\sigma_1 & \cdots & \vdots\\ \vdots & \ddots & \vdots\\ \vdots & \cdots & \sigma_T\\ 0 & \cdots & 0\\ \vdots & \ddots & \vdots\\ 0 & \cdots & 0\end{bmatrix}$ is a $S\times T$ rectangular diagonal matrix, with diagonal entries $\sigma_1\ge\sigma_2\ge\cdots\ge\sigma_T>0$ being the singular values of $\boldsymbol{A}$. $\boldsymbol{U}=(\boldsymbol{u}_1,\boldsymbol{u}_2,\cdots,\boldsymbol{u}_S)\in\mathbb{R}^{S\times S}$ with $\boldsymbol{u}_i, 1\le i\le S$ being the left singular vectors of $\boldsymbol{A}$, and $\boldsymbol{V}=(\boldsymbol{v}_1,\boldsymbol{v}_2,\cdots,\boldsymbol{v}_T)\in\mathbb{R}^{T\times T}$ with $\boldsymbol{v}_i, 1\le i\le T$ being the right singular vectors of $\boldsymbol{A}$.

**Proof.** By the orthogonality of $\boldsymbol{U}$ and $\boldsymbol{V}$, we have

$$\|\boldsymbol{b}-\boldsymbol{A\phi}\|_2^2=\|\boldsymbol{U}'(\boldsymbol{b}-\boldsymbol{AVV}'\boldsymbol{\phi})\|_2^2=\|\boldsymbol{U}'\boldsymbol{b}-\boldsymbol{\Sigma V}'\boldsymbol{\phi}\|_2^2.\tag{S16}$$

We denote

$$\boldsymbol{z} = \boldsymbol{V}'\boldsymbol{\phi} = (z_1, z_2, \cdots, z_T)' \in \mathbb{R}^T,$$

then we have

$$\min_{\boldsymbol{\phi}} \|\boldsymbol{b} - \boldsymbol{A\phi}\|_2^2 = \min_{\boldsymbol{z}} \left[\sum_{i=1}^{T} (\sigma_i z_i - \boldsymbol{u}_i'\boldsymbol{b})^2 + \sum_{i=T+1}^{S} (\boldsymbol{u}_i'\boldsymbol{b})^2\right], \tag{S17}$$

Therefore, the solution of the LSP is given by $\boldsymbol{z} = \boldsymbol{V}'\boldsymbol{\phi}$ with

$$z_i = \frac{\boldsymbol{u}_i'\boldsymbol{b}}{\sigma_i}, 1 \le i \le T. \tag{S18}$$

In other words, the LSS of Eq. (S10) is given by

$$\boldsymbol{\phi} = \sum_{i=1}^{T} \frac{\boldsymbol{u}_i'\boldsymbol{b}}{\sigma_i}\boldsymbol{v}_i = \begin{bmatrix} \frac{\boldsymbol{u}_1'\boldsymbol{b}}{\sigma_1}v_{11} + \frac{\boldsymbol{u}_2'\boldsymbol{b}}{\sigma_2}v_{21} + \cdots + \frac{\boldsymbol{u}_T'\boldsymbol{b}}{\sigma_T}v_{T1} \\ \frac{\boldsymbol{u}_1'\boldsymbol{b}}{\sigma_1}v_{12} + \frac{\boldsymbol{u}_2'\boldsymbol{b}}{\sigma_2}v_{22} + \cdots + \frac{\boldsymbol{u}_T'\boldsymbol{b}}{\sigma_T}v_{T2} \\ \vdots \\ \frac{\boldsymbol{u}_1'\boldsymbol{b}}{\sigma_1}v_{1T} + \frac{\boldsymbol{u}_2'\boldsymbol{b}}{\sigma_2}v_{2T} + \cdots + \frac{\boldsymbol{u}_T'\boldsymbol{b}}{\sigma_T}v_{TT} \end{bmatrix}. \tag{S19}$$

According to Eq. (S19), we get

$$\boldsymbol{\phi} = \sum_{i=1}^{T} \frac{\boldsymbol{u}_i'\boldsymbol{b}}{\sigma_i}\boldsymbol{v}_i, \widehat{\boldsymbol{\phi}} = \boldsymbol{\phi} + \boldsymbol{\delta\phi} = \sum_{i=1}^{T} \frac{\boldsymbol{u}_i'(\boldsymbol{b} + \delta\boldsymbol{b})}{\sigma_i}\boldsymbol{v}_i.$$

Therefore,

$$\boldsymbol{\delta\phi} = \widehat{\boldsymbol{\phi}} - \boldsymbol{\phi} = \sum_{i=1}^{T} \frac{\boldsymbol{u}_i'\delta\boldsymbol{b}}{\sigma_i}\boldsymbol{v}_i = \begin{bmatrix} \frac{\boldsymbol{u}_1'\delta\boldsymbol{b}}{\sigma_1}v_{11} + \frac{\boldsymbol{u}_2'\delta\boldsymbol{b}}{\sigma_2}v_{21} + \cdots + \frac{\boldsymbol{u}_T'\delta\boldsymbol{b}}{\sigma_T}v_{T1} \\ \frac{\boldsymbol{u}_1'\delta\boldsymbol{b}}{\sigma_1}v_{12} + \frac{\boldsymbol{u}_2'\delta\boldsymbol{b}}{\sigma_2}v_{22} + \cdots + \frac{\boldsymbol{u}_T'\delta\boldsymbol{b}}{\sigma_T}v_{T2} \\ \vdots \\ \frac{\boldsymbol{u}_1'\delta\boldsymbol{b}}{\sigma_1}v_{1T} + \frac{\boldsymbol{u}_2'\delta\boldsymbol{b}}{\sigma_2}v_{2T} + \cdots + \frac{\boldsymbol{u}_T'\delta\boldsymbol{b}}{\sigma_T}v_{TT} \end{bmatrix}. \blacksquare$$

**Remark S5.** The perturbation in observed data $\boldsymbol{b}$ is the most common scenario in real RNA-seq data. Eq. (S15) gives an analytical expression for quantification error of the $T$ gene isoforms encoded by the same gene. The error is related to singular values and

singular vectors of the read-isoform alignment conditional probability matrix $\boldsymbol{A}$. Especially, quantification error $\delta\boldsymbol{\phi}$ in data can be magnified by small singular values of $\boldsymbol{A}$:

(i) when $K(\boldsymbol{A})$ is very low, i.e., $\frac{\sigma_1(\boldsymbol{A})}{\sigma_T(\boldsymbol{A})} \approx 1$, all singular values $\sigma_i$'s are very similar. Therefore, quantification errors $\delta\phi_t, 1 \leq t \leq T$ of the $T$ gene isoforms are likely to be similar;

(ii) when $K(\boldsymbol{A})$ is very high, especially when $\frac{\sigma_{T-1}(\boldsymbol{A})}{\sigma_T(\boldsymbol{A})} \gg 1$, the quantification errors $\delta\phi_t, 1 \leq t \leq T$ of the $T$ gene isoforms are determined mainly by $\frac{\boldsymbol{u}_T'\delta\boldsymbol{b}}{\sigma_T}\boldsymbol{v}_T$. Since the parameter $\frac{\boldsymbol{u}_T'\delta\boldsymbol{b}}{\sigma_T}$ are the same for the $T$ gene isoforms, their quantification errors are determined mainly by $\boldsymbol{v}_T$, the right singular vector corresponding to the smallest singular value $\sigma_T$. The larger the element $v_{Ti}$ in $\boldsymbol{v}_T$ is, the larger the quantification error of gene isoform $i$ is.

- **3.1.2. Sensitivity to only a perturbation in $\boldsymbol{A}$**

Accurate identification of truly expressed gene isoforms is crucial for precise isoform quantification. However, current techniques, especially with short reads, face challenges in accurately determining the structure of truly expressed gene isoforms in specific samples. Consequently, the read-isoform alignment conditional probability matrix $\boldsymbol{A}$ is also sometimes inaccurate for specific isoforms, given potentially missed or falsely identified regions. In this subsection, the magnitude of quantification error is explored when only $\boldsymbol{A}$ is perturbed.

**Theorem S3.** Assume $\boldsymbol{A}$ is perturbed to $\widehat{\boldsymbol{A}} = \boldsymbol{A} + \delta\boldsymbol{A}$ in linear system Eq. (S10). Suppose $\widehat{\boldsymbol{A}}$ is a full-column-rank matrix. Denote the LSS of Eq. (S10) as $\boldsymbol{\phi}$, and the solution to the perturbed LSP of Eq. (S10) as $\widehat{\boldsymbol{\phi}} = \boldsymbol{\phi} + \delta\boldsymbol{\phi}$. Then when $K(\boldsymbol{A})\frac{\|\delta\boldsymbol{A}\|_2}{\|\boldsymbol{A}\|_2} < 1$,

$$\frac{\|\delta\boldsymbol{\phi}\|_2}{\|\boldsymbol{\phi}\|_2} \leq \frac{K(\boldsymbol{A})}{1 - K(\boldsymbol{A})\frac{\|\delta\boldsymbol{A}\|_2}{\|\boldsymbol{A}\|_2}}\left(\tan\rho + \frac{\|\delta\boldsymbol{A}\|_2}{\|\boldsymbol{A}\|_2}\right), \tag{S20}$$

where $\tan\rho = \frac{\|\boldsymbol{b}-\boldsymbol{A\phi}\|_2}{\|\boldsymbol{A\phi}\|_2}$ and $\rho$ being the angle between $\boldsymbol{b}$ and $\boldsymbol{A\phi}$.

**Proof.** Rearranging the terms in

$$(\boldsymbol{A} + \delta\boldsymbol{A})(\boldsymbol{\phi} + \delta\boldsymbol{\phi}) = \boldsymbol{b},$$

we have

$$\boldsymbol{A\phi} + \boldsymbol{A}\delta\boldsymbol{\phi} + \delta\boldsymbol{A\phi} + \delta\boldsymbol{A}\delta\boldsymbol{\phi} = \boldsymbol{b}.$$

$$(\boldsymbol{A} + \delta\boldsymbol{A})\delta\boldsymbol{\phi} = \boldsymbol{b} - \boldsymbol{A\phi} - \delta\boldsymbol{A\phi}. \quad \text{(S21)}$$

Solving Eq. (S21), the LSS for $\delta\boldsymbol{\phi}$ is

$$\delta\boldsymbol{\phi} = (\boldsymbol{A} + \delta\boldsymbol{A})^{+}(\boldsymbol{b} - \boldsymbol{A\phi} - \delta\boldsymbol{A\phi}) = (\boldsymbol{A} + \delta\boldsymbol{A})^{+}(\boldsymbol{b} - \boldsymbol{A\phi}) - (\boldsymbol{A} + \delta\boldsymbol{A})^{+}\delta\boldsymbol{A\phi}.$$

By lemma 20.11 in ref [15], when $K(\boldsymbol{A})\frac{\|\delta\boldsymbol{A}\|_2}{\|\boldsymbol{A}\|_2} < 1$, we have

$$\|(\boldsymbol{A} + \delta\boldsymbol{A})^{+}\|_2 \leq \frac{1}{1 - K(\boldsymbol{A})\frac{\|\delta\boldsymbol{A}\|_2}{\|\boldsymbol{A}\|_2}}\|\boldsymbol{A}^{+}\|_2$$

According to the properties of matrix norm, we have

$$\|\delta\boldsymbol{\phi}\|_2 \leq \|(\boldsymbol{A} + \delta\boldsymbol{A})^{+}\|_2 \cdot \|\boldsymbol{b} - \boldsymbol{A\phi}\|_2 + \|(\boldsymbol{A} + \delta\boldsymbol{A})^{+}\|_2 \cdot \|\delta\boldsymbol{A}\|_2 \cdot \|\boldsymbol{\phi}\|_2$$

$$\leq \frac{1}{1 - K(\boldsymbol{A})\frac{\|\delta\boldsymbol{A}\|_2}{\|\boldsymbol{A}\|_2}}(\|\boldsymbol{A}^{+}\|_2 \cdot \|\boldsymbol{b} - \boldsymbol{A\phi}\|_2 + \|\boldsymbol{A}^{+}\|_2 \cdot \|\delta\boldsymbol{A}\|_2 \cdot \|\boldsymbol{\phi}\|_2)$$

$$= \frac{K(\boldsymbol{A})}{1 - K(\boldsymbol{A})\frac{\|\delta\boldsymbol{A}\|_2}{\|\boldsymbol{A}\|_2}}\left(\frac{\|\boldsymbol{b} - \boldsymbol{A\phi}\|_2}{\|\boldsymbol{A}\|_2} + \frac{\|\delta\boldsymbol{A}\|_2}{\|\boldsymbol{A}\|_2}\|\boldsymbol{\phi}\|_2\right).$$

Therefore,

$$\frac{\|\delta\boldsymbol{\phi}\|_2}{\|\boldsymbol{\phi}\|_2} \leq \frac{K(\boldsymbol{A})}{1 - K(\boldsymbol{A})\frac{\|\delta\boldsymbol{A}\|_2}{\|\boldsymbol{A}\|_2}}\left(\frac{\|\boldsymbol{b} - \boldsymbol{A\phi}\|_2}{\|\boldsymbol{A}\|_2\|\boldsymbol{\phi}\|_2} + \frac{\|\delta\boldsymbol{A}\|_2}{\|\boldsymbol{A}\|_2}\right).. \quad \text{(S22)}$$

Since

$$\frac{\|\boldsymbol{b}-\boldsymbol{A\phi}\|_2}{\|\boldsymbol{A}\|_2\cdot\|\boldsymbol{\phi}\|_2}\leq\frac{\|\boldsymbol{b}-\boldsymbol{A\phi}\|_2}{\|\boldsymbol{A\phi}\|_2}=\tan\rho,$$

combing with Eq. (S22), we get

$$\frac{\|\delta\boldsymbol{\phi}\|_2}{\|\boldsymbol{\phi}\|_2}\leq\frac{K(\boldsymbol{A})}{1-K(\boldsymbol{A})\frac{\|\delta\boldsymbol{A}\|_2}{\|\boldsymbol{A}\|_2}}\left(\tan\rho+\frac{\|\delta\boldsymbol{A}\|_2}{\|\boldsymbol{A}\|_2}\right).\blacksquare \tag{S22}$$

- **3.1.3. Sensitivity to perturbations in both $\boldsymbol{A}$ and $\boldsymbol{b}$**

In this subsection, the more general case is considered, where both $\boldsymbol{A}$ and $\boldsymbol{b}$ are perturbed.

**Theorem S4.** Assume $\boldsymbol{A}$ is perturbed to $\widehat{\boldsymbol{A}}=\boldsymbol{A}+\delta\boldsymbol{A}$, and $\boldsymbol{b}$ is perturbed to $\widehat{\boldsymbol{b}}=b+\delta\boldsymbol{b}$ in linear system Eq. (S10). Suppose $\widehat{\boldsymbol{A}}$ is a full-column-rank matrix. Denote the LSS of Eq. (S10) as $\boldsymbol{\phi}$, and the solution to the perturbed LSP of Eq. (S10) as $\widehat{\boldsymbol{\phi}}=\boldsymbol{\phi}+\delta\boldsymbol{\phi}$. Then when $K(\boldsymbol{A})\frac{\|\delta\boldsymbol{A}\|_2}{\|\boldsymbol{A}\|_2}<1$,

$$\frac{\|\delta\boldsymbol{\phi}\|_2}{\|\boldsymbol{\phi}\|_2}\leq\frac{K(\boldsymbol{A})}{1-K(\boldsymbol{A})\cdot\frac{\|\delta\boldsymbol{A}\|_2}{\|\boldsymbol{A}\|_2}}\left(\tan\rho+\sec\rho\cdot\frac{\|\delta\boldsymbol{b}\|_2}{\|\boldsymbol{b}\|_2}+\frac{\|\delta\boldsymbol{A}\|_2}{\|\boldsymbol{A}\|_2}\right), \tag{S23}$$

where $\sec\rho=\frac{\|\boldsymbol{b}\|_2}{\|\boldsymbol{A\phi}\|_2}$, $\tan\rho=\frac{\|\boldsymbol{b}-\boldsymbol{A\phi}\|_2}{\|\boldsymbol{A\phi}\|_2}$ and $\rho$ being the angle between $\boldsymbol{b}$ and $\boldsymbol{A\phi}$.

**Proof.** Rearranging the terms in

$$(\boldsymbol{A}+\delta\boldsymbol{A})(\boldsymbol{\phi}+\delta\boldsymbol{\phi})=\boldsymbol{b}+\delta\boldsymbol{b},$$

we have

$$\boldsymbol{A\phi}+\boldsymbol{A}\delta\boldsymbol{\phi}+\delta\boldsymbol{A\phi}+\delta\boldsymbol{A}\delta\boldsymbol{\phi}=\boldsymbol{b}+\delta\boldsymbol{b}.$$

$$(\boldsymbol{A}+\delta\boldsymbol{A})\delta\boldsymbol{\phi}=\boldsymbol{b}-\boldsymbol{A\phi}+\delta\boldsymbol{b}-\delta\boldsymbol{A\phi}. \tag{S24}$$

Solving Eq. (S24), the LSS for $\delta\boldsymbol{\phi}$ is

$$\delta\boldsymbol{\phi}=(\boldsymbol{A}+\delta\boldsymbol{A})^{+}(\boldsymbol{b}-\boldsymbol{A\phi}+\delta\boldsymbol{b}-\delta\boldsymbol{A\phi})$$

$$= (\boldsymbol{A} + \delta\boldsymbol{A})^{+}(\boldsymbol{b} - \boldsymbol{A}\boldsymbol{\phi}) + (\boldsymbol{A} + \delta\boldsymbol{A})^{+}\delta\boldsymbol{b} - (\boldsymbol{A} + \delta\boldsymbol{A})^{+}\delta\boldsymbol{A}\boldsymbol{\phi}.$$

Similarly, by lemma 20.11 in ref [15], when $K(\boldsymbol{A})\frac{\|\delta\boldsymbol{A}\|_2}{\|\boldsymbol{A}\|_2} < 1$, we have

$$\|(\boldsymbol{A} + \delta\boldsymbol{A})^{+}\|_2 \le \frac{1}{1 - K(\boldsymbol{A})\frac{\|\delta\boldsymbol{A}\|_2}{\|\boldsymbol{A}\|_2}}\|\boldsymbol{A}^{+}\|_2$$

According to the properties of matrix norm, we have

$$\|\delta\boldsymbol{\phi}\|_2 \le \|(\boldsymbol{A} + \delta\boldsymbol{A})^{+}\|_2 \cdot \|\boldsymbol{b} - \boldsymbol{A}\boldsymbol{\phi}\|_2 + \|(\boldsymbol{A} + \delta\boldsymbol{A})^{+}\|_2 \cdot \|\delta\boldsymbol{b}\|_2 + \|(\boldsymbol{A} + \delta\boldsymbol{A})^{+}\|_2 \cdot \|\delta\boldsymbol{A}\|_2 \cdot \|\boldsymbol{\phi}\|_2$$

$$\le \frac{1}{1 - K(\boldsymbol{A})\frac{\|\delta\boldsymbol{A}\|_2}{\|\boldsymbol{A}\|_2}}(\|\boldsymbol{A}^{+}\|_2 \cdot \|\boldsymbol{b} - \boldsymbol{A}\boldsymbol{\phi}\|_2 + \|\boldsymbol{A}^{+}\|_2 \cdot \|\delta\boldsymbol{b}\|_2 + \|\boldsymbol{A}^{+}\|_2 \cdot \|\delta\boldsymbol{A}\|_2 \cdot \|\boldsymbol{\phi}\|_2)$$

$$= \frac{K(\boldsymbol{A})}{1 - K(\boldsymbol{A})\frac{\|\delta\boldsymbol{A}\|_2}{\|\boldsymbol{A}\|_2}}\left(\frac{\|\boldsymbol{b} - \boldsymbol{A}\boldsymbol{\phi}\|_2}{\|\boldsymbol{A}\|_2} + \frac{\|\delta\boldsymbol{b}\|_2}{\|\boldsymbol{A}\|_2} + \frac{\|\delta\boldsymbol{A}\|_2}{\|\boldsymbol{A}\|_2}\|\boldsymbol{\phi}\|_2\right).$$

Therefore, we have

$$\frac{\|\delta\boldsymbol{\phi}\|_2}{\|\boldsymbol{\phi}\|_2} \le \frac{K(\boldsymbol{A})}{1 - K(\boldsymbol{A})\frac{\|\delta\boldsymbol{A}\|_2}{\|\boldsymbol{A}\|_2}}\left(\frac{\|\boldsymbol{b} - \boldsymbol{A}\boldsymbol{\phi}\|_2}{\|\boldsymbol{A}\|_2\|\boldsymbol{\phi}\|_2} + \frac{\|\boldsymbol{b}\|_2}{\|\boldsymbol{A}\|_2\|\boldsymbol{\phi}\|_2}\frac{\|\delta\boldsymbol{b}\|_2}{\|\boldsymbol{b}\|_2} + \frac{\|\delta\boldsymbol{A}\|_2}{\|\boldsymbol{A}\|_2}\right). \quad \text{(S25)}$$

Since

$$\frac{\|\boldsymbol{b} - \boldsymbol{A}\boldsymbol{\phi}\|_2}{\|\boldsymbol{A}\|_2 \cdot \|\boldsymbol{\phi}\|_2} \le \frac{\|\boldsymbol{b} - \boldsymbol{A}\boldsymbol{\phi}\|_2}{\|\boldsymbol{A}\boldsymbol{\phi}\|_2} = \tan\rho,$$

$$\frac{\|\boldsymbol{b}\|_2}{\|\boldsymbol{A}\|_2 \cdot \|\boldsymbol{\phi}\|_2} \le \frac{\|\boldsymbol{b}\|_2}{\|\boldsymbol{A}\boldsymbol{\phi}\|_2} = \sec\rho,$$

Combing with Eq. (S25), we get

$$\frac{\|\delta\boldsymbol{\phi}\|_2}{\|\boldsymbol{\phi}\|_2} \le \frac{K(\boldsymbol{A})}{1 - K(\boldsymbol{A}) \cdot \frac{\|\delta\boldsymbol{A}\|_2}{\|\boldsymbol{A}\|_2}}\left(\tan\rho + \sec\rho \cdot \frac{\|\delta\boldsymbol{b}\|_2}{\|\boldsymbol{b}\|_2} + \frac{\|\delta\boldsymbol{A}\|_2}{\|\boldsymbol{A}\|_2}\right). \blacksquare$$

## 3.2. Quantification errors for non-full-column-rank genes

In this section, the quantification error of genes is studied when matrix $\boldsymbol{A}$ is non-full-column-rank.

**Theorem S5.** Consider the linear system Eq. (S10) with non-full-column-rank i.e., $\mathrm{rank}(\boldsymbol{A}) = r < T$. Assume $\boldsymbol{A}$ is perturbed to $\widehat{\boldsymbol{A}} = \boldsymbol{A} + \delta\boldsymbol{A}$, and $\boldsymbol{b}$ is perturbed to $\widehat{\boldsymbol{b}} = \boldsymbol{b} + \delta\boldsymbol{b}$. Assume $\mathrm{rank}(\boldsymbol{A}) = \mathrm{rank}(\widehat{\boldsymbol{A}})$. Denote the LSS of Eq. (S10) as $\boldsymbol{\phi}$, and the solution to the perturbed LSP of Eq. (S10) as $\widehat{\boldsymbol{\phi}} = \boldsymbol{\phi} + \delta\boldsymbol{\phi}$. Then when $K(\boldsymbol{A})\frac{\|\delta\boldsymbol{A}\|_2}{\|\boldsymbol{A}\|_2} < 1$,

$$\frac{\|\delta\boldsymbol{\phi}\|_2}{\|\boldsymbol{\phi}\|_2} \leq \frac{K(\boldsymbol{A})}{1 - K(\boldsymbol{A}) \cdot \frac{\|\delta\boldsymbol{A}\|_2}{\|\boldsymbol{A}\|_2}}\left(\tan\rho \cdot K(\boldsymbol{A}) \cdot \frac{\|\delta\boldsymbol{A}\|_2}{\|\boldsymbol{A}\|_2} + \sec\rho \cdot \frac{\|\delta\boldsymbol{b}\|_2}{\|\boldsymbol{b}\|_2} + \frac{\|\delta\boldsymbol{A}\|_2}{\|\boldsymbol{A}\|_2}\right) + K(\boldsymbol{A}) \cdot \frac{\|\delta\boldsymbol{A}\|_2}{\|\boldsymbol{A}\|_2}. \quad \text{(S28)}$$

where $\sec\rho = \frac{\|\boldsymbol{b}\|_2}{\|\boldsymbol{A\phi}\|_2}$, $\tan\rho = \frac{\|\boldsymbol{b}-\boldsymbol{A\phi}\|_2}{\|\boldsymbol{A\phi}\|_2}$ and $\rho$ being the angle between $\boldsymbol{b}$ and $\boldsymbol{A\phi}$.

**Proof.** By Theorem 2.1 in ref[16],

$$\frac{\|\delta\boldsymbol{\phi}\|_2}{\|\boldsymbol{\phi}\|_2} \leq \frac{K(\boldsymbol{A})}{1 - K(\boldsymbol{A}) \cdot \frac{\|\delta\boldsymbol{A}\|_2}{\|\boldsymbol{A}\|_2}}\left(\frac{\|\boldsymbol{b}-\boldsymbol{A\phi}\|_2}{\|\boldsymbol{A}\|_2 \cdot \|\boldsymbol{\phi}\|_2} \cdot K(\boldsymbol{A}) \cdot \frac{\|\delta\boldsymbol{A}\|_2}{\|\boldsymbol{A}\|_2} + \frac{\|\boldsymbol{b}\|_2}{\|\boldsymbol{A}\|_2 \cdot \|\boldsymbol{\phi}\|_2} \cdot \frac{\|\delta\boldsymbol{b}\|_2}{\|\boldsymbol{b}\|_2} + \frac{\|\delta\boldsymbol{A}\|_2}{\|\boldsymbol{A}\|_2}\right) + K(\boldsymbol{A}) \cdot \frac{\|\delta\boldsymbol{A}\|_2}{\|\boldsymbol{A}\|_2}.$$

Since

$$\frac{\|\boldsymbol{b}-\boldsymbol{A\phi}\|_2}{\|\boldsymbol{A}\|_2 \cdot \|\boldsymbol{\phi}\|_2} \leq \frac{\|\boldsymbol{b}-\boldsymbol{A\phi}\|_2}{\|\boldsymbol{A\phi}\|_2} = \tan\rho,$$

$$\frac{\|\boldsymbol{b}\|_2}{\|\boldsymbol{A}\|_2 \cdot \|\boldsymbol{\phi}\|_2} \leq \frac{\|\boldsymbol{b}\|_2}{\|\boldsymbol{A\phi}\|_2} = \sec\rho,$$

we get

$$\frac{\|\delta\boldsymbol{\phi}\|_2}{\|\boldsymbol{\phi}\|_2} \leq \frac{K(\boldsymbol{A})}{1 - K(\boldsymbol{A}) \cdot \frac{\|\delta\boldsymbol{A}\|_2}{\|\boldsymbol{A}\|_2}}\left(\tan\rho \cdot K(\boldsymbol{A}) \cdot \frac{\|\delta\boldsymbol{A}\|_2}{\|\boldsymbol{A}\|_2} + \sec\rho \cdot \frac{\|\delta\boldsymbol{b}\|_2}{\|\boldsymbol{b}\|_2} + \frac{\|\delta\boldsymbol{A}\|_2}{\|\boldsymbol{A}\|_2}\right) + K(\boldsymbol{A}) \cdot \frac{\|\delta\boldsymbol{A}\|_2}{\|\boldsymbol{A}\|_2}. \blacksquare$$

**Remark S6.** Assume $\|\delta\boldsymbol{A}\|_2 \ll \|\boldsymbol{A}\|_2$. When $K(\boldsymbol{A})$ is low, $K(\boldsymbol{A})\frac{\|\delta\boldsymbol{A}\|_2}{\|\boldsymbol{A}\|_2} \ll 1$, $\frac{K(\boldsymbol{A})}{1-K(\boldsymbol{A})\frac{\|\delta\boldsymbol{A}\|_2}{\|\boldsymbol{A}\|_2}} \approx K(\boldsymbol{A})$. And if the error $\boldsymbol{b}-\boldsymbol{A\phi}$ is small, angle $\rho$ is small and $\tan\rho \approx 0$, $\sec\rho \approx 1$. For full-column-rank $\boldsymbol{A}$, in such cases, in Eqs. (S11), (S20), and (S23),

A perturbation in $\boldsymbol{b}$

$$\frac{\|\delta\boldsymbol{\phi}\|_2}{\|\boldsymbol{\phi}\|_2} \lesssim K(\boldsymbol{A}) \cdot \frac{\|\delta\boldsymbol{b}\|_2}{\|\boldsymbol{b}\|_2}, \tag{S29}$$

A perturbation in $\boldsymbol{A}$

$$\frac{\|\delta\boldsymbol{\phi}\|_2}{\|\boldsymbol{\phi}\|_2} \lesssim K(\boldsymbol{A}) \frac{\|\delta\boldsymbol{A}\|_2}{\|\boldsymbol{A}\|_2}, \tag{S30}$$

Perturbations in both $\boldsymbol{A}$ and $\boldsymbol{b}$

$$\frac{\|\delta\boldsymbol{\phi}\|_2}{\|\boldsymbol{\phi}\|_2} \lesssim K(\boldsymbol{A}) \cdot \frac{\|\delta\boldsymbol{b}\|_2}{\|\boldsymbol{b}\|_2} + K(\boldsymbol{A}) \frac{\|\delta\boldsymbol{A}\|_2}{\|\boldsymbol{A}\|_2}. \tag{S31}$$

And for non-full-column-rank $\boldsymbol{A}$, as shown in Eq. (S28),

$$\frac{\|\delta\boldsymbol{\phi}\|_2}{\|\boldsymbol{\phi}\|_2} \lesssim K(\boldsymbol{A}) \cdot \frac{\|\delta\boldsymbol{b}\|_2}{\|\boldsymbol{b}\|_2} + K(\boldsymbol{A}) \frac{2\|\delta\boldsymbol{A}\|_2}{\|\boldsymbol{A}\|_2}.$$

These results demonstrate that $K(\boldsymbol{A})$ has a great influence on gene isoform abundance quantification as a relative error (RE) magnification factor: given a perturbation $\delta\boldsymbol{b}$ in $\boldsymbol{b}$ or/and $\delta\boldsymbol{A}$ in $\boldsymbol{A}$, RE of the solution, $\frac{\|\delta\boldsymbol{\phi}\|_2}{\|\boldsymbol{\phi}\|_2}$, is less than or approximates $K(\boldsymbol{A})\frac{\|\delta\boldsymbol{b}\|_2}{\|\boldsymbol{b}\|_2}$, $K(\boldsymbol{A})\frac{\|\delta\boldsymbol{A}\|_2}{\|\boldsymbol{A}\|_2}$, $K(\boldsymbol{A}) \cdot \frac{\|\delta\boldsymbol{b}\|_2}{\|\boldsymbol{b}\|_2} + K(\boldsymbol{A})\frac{\|\delta\boldsymbol{A}\|_2}{\|\boldsymbol{A}\|_2}$, or $K(\boldsymbol{A}) \cdot \frac{\|\delta\boldsymbol{b}\|_2}{\|\boldsymbol{b}\|_2} + K(\boldsymbol{A})\frac{2\|\delta\boldsymbol{A}\|_2}{\|\boldsymbol{A}\|_2}$.

**Supplementary Notes 4: Community-wise model for miniQuant**

In this note, the community-wise model for gene isoform abundance estimation used in miniQuant is introduced.

For community $c$, separate likelihood functions are built for long reads and short reads, respectively. These likelihood functions are then integrated with a weighted approach to minimize the quantification error in this community effectively.

Recall the definition of $N^c$, $M^c$, and $T^c$. $N^c$ and $M^c$ are the number of short reads and long reads assigned to community $c$ that can be mapped to $T^c$ gene isoforms. The relative abundances of the $T^c$ gene isoforms in community $c$ are denoted as $\boldsymbol{\theta}^c = (\theta_1^c, \ldots, \theta_{T^c}^c)'$ such that $\sum_{t=1}^{T^c} \theta_t^c = 1$.

Denote $R_m$ as the sequence of the $m$-th read (fragment) in community $c$, $m \in \{1, \cdots, N^c + M^c\}$, which is observed. The gene isoform of origin, length, start position, and orientation of $R_m$ are denoted as $G_m, F_m, S_m$ and $O_m$, respectively.

Define indicator $W_{mt}(f_{mt}, s_{mt}, o_{mt})$ as

$$W_{mt}(f_{mt}, s_{mt}, o_{mt}) = \begin{cases} 1, R_m \text{ can be mapped to gene isoform } t \text{ at start position } s_{mt}, \\ \qquad\qquad \text{with length } f_{mt} \text{ and orientation } o_{mt}; \\ 0, \text{otherwise.} \end{cases}$$

Assume the alignment of $R_m$ is unique if the gene isoform of origin is known. That is, for each $m$ and each $t$, there is at most one set of $(f_{mt0}, s_{mt0}, o_{mt0})$ such that $W_{mt}(f_{mt0}, s_{mt0}, o_{mt0}) = 1$. Define observed compatibility variables $\boldsymbol{X}_m = (X_{m1}, \cdots, X_{mT^c})'$ as the indicators of the existence of such a set of $(f_{mt0}, s_{mt0}, o_{mt0})$, i.e.,

$$X_{mt} = \begin{cases} 1, R_m \text{ can be mapped to gene isoform } t; \\ 0, \text{otherwise.} \end{cases}$$

Let $\mathcal{S}_m = \{t: X_{mt} = 1, 1 \le t \le T^c\}$, $1 \le m \le N^c + M^c$ be the set consisting of isoforms to which $R_m$ can be mapped.

- **Model for long reads (miniQuant-L)**

Consider a long read $R_m$ in community $c$. Without loss of generality, assume reads with indices $N^c + 1 \leq m \leq N^c + M^c$ are long reads.

The likelihood of $R_m$ is

$$L_{LR}(\boldsymbol{\theta}^c|R_m) = \sum_{t=1}^{T^c} P(R_m|G_m = t, \boldsymbol{\theta}^c)P(G_m = t|\boldsymbol{\theta}^c).$$

Specifically,

$$P(G_m = t|\boldsymbol{\theta}^c) = \theta_t^c, 1 \leq t \leq T^c,$$

If $t \in \mathcal{S}_m$,

$$P(R_m|G_m = t, \boldsymbol{\theta}^c)$$

$$= \sum_{F_m, S_m, O_m} P(R_m|F_m, S_m, O_m, G_m, \boldsymbol{\theta}^c)P(F_m|G_m = t, \boldsymbol{\theta}^c)P(S_m|F_m, G_m = t, \boldsymbol{\theta}^c)P(O_m|G_m = t, \boldsymbol{\theta}^c)$$

Without considering sequencing errors, the read is uniquely defined by $f_{mt}$, $s_{mt}$, $o_{mt}$, and $t$, thus

$$W_{mt} = P(R_m = r_m|F_m = f_{mt}, S_m = s_{mt}, O_m = o_{mt}, G_m = t)$$
$$= P(R_m = r_m|F_m = f_{mt}, S_m = s_{mt}, O_m = o_{mt}, G_m = t, \boldsymbol{\theta}^c)$$

Then by the assumption of alignment uniqueness,

$$P(R_m|G_m = t, \boldsymbol{\theta}^c)$$
$$= P(F_m = f_{mt0}|G_m = t, \boldsymbol{\theta}^c)$$
$$\times P(S_m = s_{mt0}|F_m = f_{mt0}, G_m = t, \boldsymbol{\theta}^c)P(O_m = o_{mt0}|G_m = t, \boldsymbol{\theta}^c)$$

Otherwise $P(R_m|G_m = t, \boldsymbol{\theta}^c) = 0$.

*Length distribution*

For long-read sequencing, without considering sequencing error, $f_{mt0} = f_m, \forall t \in \mathcal{S}_m$ . $f_m$ is the read length, which is the same for $\forall t \in \mathcal{S}_m$. The read length density function $\mathscr{f}(f_m)$ of long reads is given by

$$\mathscr{f}(f_m) = \mathcal{F}(f_m + 1) - \mathcal{F}(f_m).$$

$\mathcal{F}(f_m) = P(F_m < f_m)$ can be estimated by the Kaplan-Meier estimator based on real data. Then, given a gene isoform $t$ in community $c$, the isoform-specific read length probability for long reads can be approximated by

$$P(F_m = f_m | G_m = t) = \frac{\mathscr{f}(f_m)}{\sum_{F_m=1}^{l_t^c} \mathscr{f}(F_m)}.$$

Optionally, if the length distribution is not isoform specific,

$$P(F_m = f_m | G_m = t) = \mathscr{f}(f_m).$$

If length distribution is not modeled

$$P(F_m = f_m | G_m = t) = 1.$$

*Start site distribution*

The start position $S_n$ follows a uniform distribution

$$P(S_m = s_{mt0} | F_m = f_m, G_m = t) = \frac{1}{l_t^c - f_m + 1} = \frac{1}{\tilde{l}_{mt}^c}.$$

where $l_t^c$ are the length of gene isoform $t$ in community $c$.

*Orientation distribution*

The orientation $O_m$ is binary, with $O_m = 0$ indicating that the sequence of read $m$ is in the same orientation as that of its gene isoform of origin, and $O_m = 1$ indicating that it is reverse complemented. This random variable allows us to model RNA-seq protocols that are either strand-specific or not strand-specific:

$$P(O_m = 0|G_m = t) = \begin{cases} 1, & \text{protocol is strand} - \text{specific,} \\ 0.5, & \text{protocol is not strand} - \text{specific;} \end{cases}$$

$$P(O_m = 1|G_m = t) = \begin{cases} 0, & \text{protocol is strand} - \text{specific,} \\ 0.5, & \text{protocol is not strand} - \text{specific.} \end{cases}$$

For simplicity, we assume the protocol is strand-specific.

*Read sequence distribution*

Denote $b_{mt} = P(R_m = r_m |\, G_m = t,\, \boldsymbol{\theta}^c)$, then

$$b_{mt} = \frac{\mathscr{f}(f_m)}{\sum_{F_m=1}^{l_t^c} \mathscr{f}(F_m)} \cdot \frac{X_{mt}}{\tilde{l}_{mt}^c}.$$

Optional formats of $b_{mt}$ are as follows,

$$b_{mt} = \begin{cases} \mathscr{f}(f_m) \cdot \dfrac{X_{mt}}{\tilde{l}_{mt}^c}, & \text{length distribution is not isoform} - \text{specific,} \\ \dfrac{X_{mt}}{\tilde{l}_{mt}^c}, & \text{length distribution is not modeled.} \end{cases}$$

As shown in Supplementary Notes 10, the non-isoform-specific length distribution and unmodeled length distribution are equivalent in the following steps, leading to the same result.

Therefore, the complete likelihood function for all long reads in this community is

$$L_{LR}(\boldsymbol{\theta}^c|\boldsymbol{R}) = \prod_{m=1}^{M^c} \sum_{t \in \mathcal{S}_m} b_{mt}\theta_t^c \,. \qquad \text{(S32)}$$

- **Model for short reads**

To distinguish short reads from long reads, short reads are indexed by $n$ instead of by $m$. Consider a short read $R_n$ in community $c$, $1 \leq n \leq N^c$.

The likelihood of $R_n$ is

$$L_{SR}(\boldsymbol{\theta}^c|R_n) = \sum_{t=1}^{T^c} P(R_n|G_n = t, \boldsymbol{\theta}^c)P(G_n = t|\boldsymbol{\theta}^c).$$

Specifically,

$$P(G_n = t|\boldsymbol{\theta}^c) = \frac{\theta_t^c \tilde{l}_t^c}{\sum_{r=1}^{T^c} \theta_r^c \tilde{l}_r^c}, 1 \le t \le T^c,$$

where $\tilde{l}_t^c = l_t^c - \bar{F} + 1$ and $l_t^c$ are the effective length and the original length of gene isoform $t$ in community $c$, respectively. $\bar{F}$ is the mean of the truncated empirical fragment length distribution[12].

And similar to the model for long reads, if $t \in \mathcal{S}_n$,

$$P(R_n|G_n = t, \boldsymbol{\theta}^c)$$
$$= P(F_n = f_{nt0}|G_n = t, \boldsymbol{\theta}^c)$$
$$\times P(S_n = s_{nt0}|F_n = f_{nt0}, G_n = t, \boldsymbol{\theta}^c)P(O_n = o_{nt0}|G_n = t, \boldsymbol{\theta}^c)$$

Otherwise $P(R_n|G_n = t, \boldsymbol{\theta}^c) = 0$.

*Length distribution*

**For pair-end reads**, assume $f_{nt0} = f_n, \forall t \in \mathcal{S}_n$. And assume the fragment length $F_n$ follows a normal distribution $N(u, \sigma^2)$, i.e.,

$$P(F_n = f_n|G_n = t) = \frac{1}{\sigma\sqrt{2\pi}} e^{-\frac{1}{2}\left(\frac{f_n - u}{\sigma}\right)^2},$$

where $u$ and $\sigma$ are estimated based on the fragment length distribution of real data.

**For single-end read**, the probability of the read length $F_n$ is given by

$$P(F_n = L|G_n = t) = 1,$$

where $L$ is a constant read length determined by the sequencing platform.

As shown in Supplementary Notes 10, the normalized length distribution and un-

normalized length distribution are equivalent in the following steps.

*Start site distribution*

The start position $S_n$ follows a uniform distribution, with the fragment length approximated by $\bar{F}$, i.e.,

$$P(S_n = s_{nt0}|F_n = f_n, G_n = t) \approx \frac{1}{l_t^c - \bar{F} + 1} = \frac{1}{\tilde{l}_t^c}.$$

*Orientation distribution*

$$P(O_n = 0|G_n = t) = \begin{cases} 1, & \text{protocol is strand} - \text{specific}, \\ 0.5, & \text{protocol is not strand} - \text{specific}; \end{cases}$$

$$P(O_n = 1|G_n = t) = \begin{cases} 0, & \text{protocol is strand} - \text{specific}, \\ 0.5, & \text{protocol is not strand} - \text{specific}. \end{cases}$$

Similar to the long-read orientation distribution, we assume the protocol is strand-specific.

*Read sequence distribution*

Denote $a_{nt} = P(R_n|G_n = t, \boldsymbol{\theta}^c)$, then

$$a_{nt} = \begin{cases} \frac{1}{\sigma\sqrt{2\pi}} e^{-\frac{1}{2}\left(\frac{f_n-u}{\sigma}\right)^2} \cdot \frac{X_{nt}}{\tilde{l}_t^c}, R_n \text{ is pair} - \text{end}, \\ \frac{X_{nt}}{\tilde{l}_t^c}, & R_n \text{ is single} - \text{end}. \end{cases}$$

For all short reads in this community, therefore, the likelihood function is

$$L_{SR}(\boldsymbol{\theta}^c|\boldsymbol{R}) = \prod_{n=1}^{N^c} \sum_{t \in S_n} a_{nt} \frac{\theta_t^c \tilde{l}_t^c}{\sum_{r=1}^{T^c} \theta_r^c \tilde{l}_r^c}. \quad \text{(S33)}$$

- **Hybrid model to integrate short reads and long reads (miniQuant-H)**

To reduce the quantification error of each community, long and short reads in each community are given community-specific weights $\alpha_c$, and weights $1 - \alpha_c$, respectively. For a fix weight $\alpha_c$, combining equations Eq. (S32) and Eq. (S33), the likelihood function

for the hybrid model can be written as

$$L(\boldsymbol{\theta}^c|\boldsymbol{R}) = [L_{LR}(\boldsymbol{\theta}^c|\boldsymbol{R})]^{\alpha_c}[L_{SR}(\boldsymbol{\theta}^c|\boldsymbol{R})]^{1-\alpha_c},$$

and the log-likelihood function is

$$\begin{aligned} l(\boldsymbol{\theta}^c|\boldsymbol{R}) &= \alpha_c \log L_{LR}(\boldsymbol{\theta}^c|\boldsymbol{R}) + (1-\alpha_c)\log L_{SR}(\boldsymbol{\theta}^c|\boldsymbol{R}) \\ &= \alpha_c \sum_{m=1}^{M^c} \log\left(\sum_{t=1}^{\mathcal{S}_m} b_{mt}\theta_t^c\right) + (1-\alpha_c)\sum_{n=1}^{N^c}\log\left(\sum_{t=1}^{\mathcal{S}_n} a_{nt}\frac{\theta_t^c \tilde{l}_t^c}{\sum_{r=1}^{T^c}\theta_r^c \tilde{l}_r^c}\right). \qquad \text{(S34)} \end{aligned}$$

Gene isoform relative abundance $\boldsymbol{\theta}^c$ is estimated by maximizing the likelihood function, which is equivalent to the following optimization problem,

$$\widehat{\boldsymbol{\theta}}^c = \underset{\boldsymbol{\theta}^c}{\operatorname{argmax}}\ l(\boldsymbol{\theta}^c|\boldsymbol{R}),$$

$$s.t.\begin{cases}\sum_{t=1}^{T^c}\theta_t^c = 1, \\ \theta_t^c \geq 0, \quad 0 \leq t \leq T^c.\end{cases}$$

Given the difficulty of solving the above optimization problem, unobserved latent variables $\boldsymbol{Z}_n = (Z_{n1}, \cdots, Z_{nT^c})'$ are defined, with $Z_{nt}$ being the indicator variable that the gene isoform of origin for read $R_n$ is isoform $t$, i.e.,

$$Z_{nt} = \begin{cases}1, \text{gene isoform of origin for } R_n \text{ is isoform } t\,; \\ 0, \text{otherwise.}\end{cases}$$

For each read $R_n$, $\sum_{t=1}^{T^c} Z_{nt} = 1$, $1 \leq n \leq N^c + M^c$, i.e., different from $X_{nt}$'s, there is one and only one of the $Z_{nt}$'s that equal 1 for each $n$. As defined above, $\mathcal{S}_n = \{t: X_{nt} = 1, 1 \leq t \leq T^c\}$, $1 \leq n \leq N^c + M^c$, then $Z_{nt} = 1$ only if $t \in \mathcal{S}_n$; that is, $P(Z_{nt} = 1|\boldsymbol{\theta}^c) \geq 0$ if $t \in \mathcal{S}_n$ and $P(Z_{nt} = 1|\boldsymbol{\theta}^c) = 0$ if $t \notin \mathcal{S}_n$.

Further conditioning on variables $\boldsymbol{Z}$,

$$L_{LR}(\boldsymbol{\theta}^c|\boldsymbol{R},\boldsymbol{Z}) = \prod_{m=1}^{M^c}\sum_{t\in\mathcal{S}_m} P(R_m, Z_m|G_m = t, \boldsymbol{\theta}^c)P(G_m = t|\boldsymbol{\theta}^c).$$

The major difference lies in the following term:

$$P(R_m|F_m = f_{mt0}, S_m = s_{mt0}, O_m = o_{mt0}, G_m = t, \boldsymbol{\theta}^c) = 1, \text{when } t \in \mathcal{S}_m,$$

$$P(R_m, Z_m|F_m = f_{mt0}, S_m = s_{mt0}, O_m = o_{mt0}, G_m = t, \boldsymbol{\theta}^c) = 1, \text{when } Z_{mt} = 1.$$

Then, the complete data likelihood for long reads and short reads are

$$L_{LR}(\boldsymbol{\theta}^c|\boldsymbol{R}, \boldsymbol{Z}) = \prod_{m=1}^{M^c} \prod_{t \in \mathcal{S}_m} (b_{mt}\theta_t^c)^{Z_{mt}},$$

and

$$L_{SR}(\boldsymbol{\theta}^c|\boldsymbol{R}, \boldsymbol{Z}) = \prod_{n=1}^{N^c} \prod_{t \in \mathcal{S}_n} \left(a_{nt} \frac{\theta_t^c \tilde{l}_t^c}{\sum_{r=1}^{T^c} \theta_r^c \tilde{l}_r^c}\right)^{Z_{nt}},$$

respectively. Therefore, the complete data log-likelihood for the hybrid model is

$$l(\boldsymbol{\theta}^c|\boldsymbol{R}, \boldsymbol{Z}) = \alpha_c \log L_{LR}(\boldsymbol{\theta}^c|\boldsymbol{R}, \boldsymbol{Z}) + (1 - \alpha_c) \log L_{SR}(\boldsymbol{\theta}^c|\boldsymbol{R}, \boldsymbol{Z})$$

$$= \alpha_c \sum_{m=1}^{M^c} \sum_{t \in \mathcal{S}_m} Z_{mt} \log(b_{mt}\theta_t^c) + (1 - \alpha_c) \sum_{n=1}^{N^c} \sum_{t \in \mathcal{S}_n} Z_{nt} \log\left(a_{nt} \frac{\theta_t^c \tilde{l}_t^c}{\sum_{r=1}^{T^c} \theta_r^c \tilde{l}_r^c}\right).$$

Optimization is realized by EM algorithm.

**Supplementary Notes 5: Detailed discussion of the three gene sets in Fig. 3e**

In this note, the details of the three sets in Fig. 3e and their example genes are presented. By fixing the number of long reads at 1 million and increasing the number of short read pairs from 10 million to 80 million, the gap of quantification error (|ΔMARD|; MARD: mean absolute relative difference) between kallisto and miniQuant-L for the genes in Set 2 (Extended Data Fig. 7a, top left; Online Methods) with low abundance ($0<\log_2(\text{TPM}+1)\leq 2$) and slight difference of errors between kallisto and miniQuant-L ($-0.2\leq\Delta\text{MARD}<0$) becomes more extensive, leading to its merging with Set 1 with low abundance ($0<\log_2(\text{TPM}+1)\leq 2$) and larger ΔMARD ($-1\leq\Delta\text{MARD}\leq -0.7$). Since the sampling error dominates the deconvolution error for Set 2 because of their low abundance (median TPM 1.51) and small K-value (median 1.14) (see example of the gene *CPLANE2* in Fig. 3f, middle), the increase in short-read sequencing depth greatly reduces the quantification error of short reads. ΔMARD of Set 2 remains stable after the sequencing depth increases to 80 million, as the reduction in sampling error becomes marginal while the deconvolutional error becomes dominant. On the other hand, the difference of quantification error for the genes in Set 3 with high abundance ($3\leq\log_2(\text{TPM}+1)\leq 6$) and considerable gap of quantification error ($-0.35\leq\Delta\text{MARD}<0$) increases more mildly compared to Set 2, given the deconvolutional error dominates the sampling error as the K-values of these genes (median 10.29) is higher than Set 2.

With 80 million short read pairs, kallisto yields smaller MARD than miniQuant-L with 0.5 million long reads on 96.90% (31,216/32,215) genes, over half (51.81%) of which can be grouped into two sets (Extended Data Fig. 7b, top left; Online Methods): Set 4 (13,302 genes) with low abundance ($0<\log_2(\text{TPM}+1)\leq 2$) and significant difference in quantification

error between kallisto and miniQuant-L ($-1 \leq \Delta MARD \leq -0.7$) and Set 5 (3,390 genes) with high abundance ($3 \leq \log_2(TPM+1) \leq 6$) and medium gap of errors ($-0.7 \leq \Delta MARD < 0.5$). By fixing the number of short read pairs at 80 million and increasing the number of long reads from 0.5 million to 30 million, the difference in quantification error for genes in Set 4 becomes smaller, thanks to the reduction in sampling error by higher sequencing depth. Different from the behavior of Set 3 (Extended Data Fig. 7a, top left; Online Methods), the increase in long-read sequencing depth considerably lessens the difference in quantification errors of Set 5. While the K-value and abundance in Set 5 are high (median K-value 13.40 and median TPM 18.81), both deconvolution and sampling error can be reduced by long reads data of deeper sequencing depth.

## Supplementary Notes 6: Missing isoforms in the quantification output of current long read-based tools

Long read-based tools may not output quantification results for all the isoforms in the annotation. For example, TALON outputs only a few quantification results of annotated ERCCs, while LIQA does not output quantification results for genes with a single isoform.

**Supplementary Notes 7: Technical issues of the three SIRV genes excluded from analysis**

In this note, the technical issues of SIRV2, SIRV5 and SIRV7 are introduced.

SIRV2 and SIRV7 have abnormal read coverage for short and long reads, respectively (Supplementary Fig. 3c). And SIRV5 and SIRV7 suffer from misalignment when aligned using Minimap2 (v2.24) (Supplementary Fig. 4). All reads aligned to this position consistently exhibit a 6 bp deletion, resulting in a wrong allocation of reads between two SIRV transcripts with close splicing sites. Thus, SIRV2, SIRV5 and SIRV7 are excluded from the analysis.

Except technical issues of TALON and LIQA, long read-based tools exhibit higher quantification errors on SIRV5 and SIRV7 compared to short read-based methods (Supplementary Fig. 5), while miniQuant-H and short read-based tools exhibit higher quantification errors on SIRV2.

**Supplementary Notes 8: Comparison between miniQuant-H and short read-based tools using sample-specific annotation**

In this note, details about the comparison between miniQuant-H and short read-based tools using sample-specific annotation are introduced.

In the analysis of real data of spike-in RNAs, ground truth annotation, which is equivalent to sample-specific annotation, is used by all tools, including the short read-based tools. Similarly, although the annotation identified by short reads could result in great increase in quantification error, it is still practical to generate a more accurate sample-specific annotation using long reads and then perform quantification using short reads.

In this comparison, the same sample-specific annotation is used by miniQuant-H and the five short read-based tools. And similar to the spike-in RNAs where the K-values are calculated based on the ground truth annotation, in this analysis, genes are classified based on sample-specific K-values, that are calculated based on sample-specific annotation.

**Supplementary Notes 9: Fragmental reads in long-read RNA-seq**

In this note, fragmental reads in long-read RNA-seq are introduced.

While long-read sequencing techniques have the capability to produce full splice match (FSM) reads[17] that match a corresponding reference transcript at all its splice junctions, a substantial proportion of long reads exhibit fragmental matches (FM). The classification of the reads is determined using SQANTI3 (ref. [17]), with all classes except FSM reads assigned to the FM group.

Notably, cDNA-ONT sequencing tends to yield a lower proportion of FSM reads, whereas cDNA-PacBio demonstrates the highest FSM proportion among the three long-read techniques (Supplementary Fig. 1). Correspondingly, cDNA-ONT data exhibit a larger median MARD compared to cDNA-PacBio data in quantification (Fig. 3d). Reads collected with dRNA-ONT sequencing have a higher proportion of FSM reads than cDNA-ONT, but also a higher sequencing error rate. The quantification performance of dRNA-ONT data is comparable to that of cDNA-ONT data.

**Supplementary Notes 10: Details of the EM algorithm in miniQuant method**

In this note, the details of the EM algorithm are presented.

**Derivation of EM algorithm for solving the miniQuant model**

The update procedure of the EM algorithm is as follows.

*E-step*

$$
\begin{aligned}
Q\left(\boldsymbol{\theta}^c \middle| \boldsymbol{\theta}^{c,(k)}\right) &= E_{\boldsymbol{Z}|\boldsymbol{R},\boldsymbol{\theta}^{c,(k)}}\left[l(\boldsymbol{\theta}^c|\boldsymbol{R},\boldsymbol{Z})\right] \\
&= E_{\boldsymbol{Z}|\boldsymbol{R},\boldsymbol{\theta}^{c,(k)}}\left[\alpha_c \sum_{m=1}^{M^c}\sum_{t\in\mathcal{S}_m} Z_{mt}\log(b_{mt}\theta_t^c)\right. \\
&\quad \left. + (1-\alpha_c)\sum_{n=1}^{N^c}\sum_{t\in\mathcal{S}_n} Z_{nt}\log\left(a_{nt}\frac{\theta_t^c\tilde{l}_t^c}{\sum_{r=1}^{T^c}\theta_r^c\tilde{l}_r^c}\right)\right] \\
&= \alpha_c \sum_{m=1}^{M^c}\sum_{t\in\mathcal{S}_m} E_{Z_{mt}|R_m,\boldsymbol{\theta}^{c,(k)}}[Z_{mt}]\log(b_{mt}\theta_t^c) \\
&\quad + (1-\alpha_c)\sum_{n=1}^{N^c}\sum_{t\in\mathcal{S}_n} E_{Z_{nt}|R_n,\boldsymbol{\theta}^{c,(k)}}[Z_{nt}]\log\left(a_{nt}\frac{\theta_t^c\tilde{l}_t^c}{\sum_{r=1}^{T^c}\theta_r^c\tilde{l}_r^c}\right) \\
&= \alpha_c \sum_{m=1}^{M^c}\sum_{t\in\mathcal{S}_m} q(Z_{mt})\log(b_{mt}\theta_t^c) + (1-\alpha_c)\sum_{n=1}^{N^c}\sum_{t\in\mathcal{S}_n} q(Z_{nt})\log\left(a_{nt}\frac{\theta_t^c\tilde{l}_t^c}{\sum_{r=1}^{T^c}\theta_r^c\tilde{l}_r^c}\right),
\end{aligned}
$$

where $\boldsymbol{\theta}^{c,(k)}$ is the estimated $\boldsymbol{\theta}^c$ in the $k$-th iteration. For short reads,

$$
\begin{aligned}
q(Z_{nt}) &= E_{Z_{nt}|R_n,\boldsymbol{\theta}^{c,(k)}}[Z_{nt}] \\
&= P\left(Z_{nt}|R_n,\boldsymbol{\theta}^{c,(k)}\right) \\
&= \frac{P\left(Z_{nt},R_n|\boldsymbol{\theta}^{c,(k)}\right)}{P(R_n|\boldsymbol{\theta}^{c,(k)})} \\
&= \frac{P\left(R_n,Z_{nt}=1|\boldsymbol{\theta}^{c,(k)}\right)}{\sum_{i\in\mathcal{S}_n}P(R_n,Z_{ni}=1|\boldsymbol{\theta}^{c,(k)})}
\end{aligned}
$$

$$= \frac{a_{nt} \frac{\theta_t^{c,(k)} \tilde{l}_t^c}{\sum_{r=1}^{T^c} \theta_r^{c,(k)} \tilde{l}_r^c}}{\sum_{i \in \mathcal{S}_n} a_{ni} \frac{\theta_i^{c,(k)} \tilde{l}_i^c}{\sum_{r=1}^{T^c} \theta_r^{c,(k)} \tilde{l}_r^c}}.$$

Similarly, for long reads

$$q(Z_{mt}) = \frac{b_{mt} \theta_t^{c,(k)}}{\sum_{i \in \mathcal{S}_m} b_{mi} \theta_i^{c,(k)}}.$$

*M-step*

$\boldsymbol{\theta}^c$ that maximizes $Q\left(\boldsymbol{\theta}^c \middle| \boldsymbol{\theta}^{c,(k)}\right)$ is found under the constraint $\sum_{t=1}^{T^c} \theta_t^c = 1$, i.e.,

$$\underset{\boldsymbol{\theta}^c}{\operatorname{argmax}}\, Q\left(\boldsymbol{\theta}^c | \boldsymbol{\theta}^{c,(k)}\right)$$

$$s.t. \begin{cases} \sum_{t=1}^{T^c} \theta_t^c = 1, \\ \theta_t^c \geq 0, \quad\quad 0 \leq t \leq T^c. \end{cases}$$

Lagrange multiplier method is used to solve this constrained maximization problem. The Lagrangian function is

$$h(\boldsymbol{\theta}^c, \lambda) = Q\left(\boldsymbol{\theta}^c \middle| \boldsymbol{\theta}^{c,(k)}\right) - \lambda \left( \sum_{t=1}^{T^c} \theta_t^c - 1 \right). \quad \text{(S35)}$$

Take the derivatives of Eq. (S35) with respect to $\theta_t^c, 1 \leq t \leq T^c$ and $\lambda$, and set them to 0, we have

$$\frac{\partial h(\boldsymbol{\theta}^c, \lambda)}{\partial \theta_t^c} = \frac{\partial Q\left(\boldsymbol{\theta}^c \middle| \boldsymbol{\theta}^{c,(k)}\right)}{\partial \theta_t^c} - \frac{\partial \lambda \left(\sum_{t=1}^{T^c} \theta_t^c - 1\right)}{\partial \theta_t^c} = 0, \quad \text{(S36)}$$

and

$$\frac{\partial h(\boldsymbol{\theta}^c, \lambda)}{\partial \lambda} = \sum_{t=1}^{T^c} \theta_t^c - 1 = 0.$$

Eq. (S36) is equivalent to

$$\alpha_c \frac{1}{\theta_t^c} \sum_{m=1}^{M^c} q(Z_{mt}) + (1-\alpha_c) \frac{1}{\theta_t^c} \sum_{n=1}^{N^c} q(Z_{nt}) = (1-\alpha_c) \frac{\tilde{l}_t^c}{\sum_{r=1}^{T^c} \theta_r^c \tilde{l}_r^c} \sum_{n=1}^{N^c} \sum_{t' \in \mathcal{S}_n} q(Z_{nt'}) + \lambda. \quad \text{(S37)}$$

Taking summation over $t$ on both sides of Eq. (S37), we have

$$\alpha_c \sum_{t=1}^{T^c} \sum_{m=1}^{M^c} q(Z_{mt}) + (1-\alpha_c) \sum_{t=1}^{T^c} \sum_{n=1}^{N^c} q(Z_{nt})$$

$$= (1-\alpha_c) \sum_{t=1}^{T^c} \frac{\theta_t^c \tilde{l}_t^c}{\sum_{r=1}^{T^c} \theta_r^c \tilde{l}_r^c} \sum_{n=1}^{N^c} \sum_{t' \in \mathcal{S}_n} q(Z_{nt'}) + \lambda \sum_{t=1}^{T^c} \theta_t^c.$$

Then, we get

$$\lambda = \alpha_c \sum_{t=1}^{T^c} \sum_{m=1}^{M^c} q(Z_{mt}) = \alpha_c \sum_{m=1}^{M^c} \sum_{t \in \mathcal{S}_m} q(Z_{mt}),$$

and

$$\theta_t^c = \frac{\alpha_c \sum_{m=1}^{M^c} q(Z_{mt}) + (1-\alpha_c) \sum_{n=1}^{N^c} q(Z_{nt})}{\alpha_c \sum_{m=1}^{M^c} \sum_{t' \in \mathcal{S}_m} q(Z_{mt'}) + (1-\alpha_c) \frac{\tilde{l}_t^c}{\sum_{r=1}^{T^c} \theta_r^c \tilde{l}_r^c} \sum_{n=1}^{N^c} \sum_{t' \in \mathcal{S}_n} q(Z_{nt'})}$$

$$= \frac{\alpha_c \sum_{m=1}^{M^c} q(Z_{mt}) + (1-\alpha_c) \sum_{n=1}^{N^c} q(Z_{nt})}{\alpha_c M^c + (1-\alpha_c) \frac{\tilde{l}_t^c}{\sum_{r=1}^{T^c} \theta_r^c \tilde{l}_r^c} N^c}.$$

Therefore,

$$\theta_t^{c,(k+1)} = \frac{\alpha_c \sum_{m=1}^{M^c} q(Z_{mt}) + (1-\alpha_c) \sum_{n=1}^{N^c} q(Z_{nt})}{\alpha_c M^c + (1-\alpha_c) \frac{\tilde{l}_t^c}{\sum_{r=1}^{T^c} \theta_r^{c,(k)} \tilde{l}_r^c} N^c},$$

where the denominator is approximated by the value of $\boldsymbol{\theta}^c$ in the $k$-th iteration.

Additionally, as shown above, $\theta_t^{c,(k+1)}$ is correlated with $a_{nt}$ and $b_{mt}$ only via $q(Z_{nt})$ and $q(Z_{mt})$. Considering the structure of $q(Z_{nt})$, only the terms correlated with $t$ in $a_{nt}$ and $b_{mt}$ are left after simplifying the fraction. Consequently, the non-isoform-specific length distribution and unmodeled length distribution are equivalent, and similarly, the normalized and unnormalized non-isoform-specific length distributions are also equivalent.

**Supplementary Notes 11: Filtering of GENCODE annotation**

In this note, the filtering of GENCODE annotation is introduced.

The isoforms with the biotypes of “Mt_rRNA, Mt_tRNA, miRNA, misc_RNA, rRNA, scRNA, snRNA, snoRNA, ribozyme, sRNA, scaRNA” (see https://www.gencodegenes.org/pages/biotypes.html), and the isoforms located within the less reliable regions of the reference genome are filtered out. The less reliable regions are the regions with the presence of unknown nucleotides N instead of A, C, G, and T in the initial release of the hg38 reference genome. We also exclude genes for which two or more isoforms, despite having distinct isoform ID numbers, share identical structures.

## Supplementary Notes 12: Estimation of long read characteristics

In this note, the estimation of long read characteristics is introduced.

The read lengths and the overall error rates for long reads are estimated from real data using AlignQC[18]. The cDNA-PacBio (accession: ENCFF235QXW), cDNA-ONT (accession: GSM8239068), and dRNA-ONT (accession: GSM8239069) data (Supplementary Tables 4 and 5) for ESC are used.

The proportion of full splice match reads for long reads are estimated from real data using SQANTI3[19]. The cDNA-PacBio (accession: ENCFF235QXW), cDNA-ONT (accession: ENCFF326IBB), and dRNA-ONT (accession: ENCFF207KVO) for ESC in LRGASP consortium (Supplementary Table 4) are used.

**Supplementary Notes 13: Details of the EM algorithm in miniQuant method**

In this note, additional simulation datasets used in specific analyses are introduced. Simulation datasets for the typical gene examples (Figs. 2a and 3a) contain only the corresponding target gene. In Fig. 2a, for gene *FAM219A* (ENSG00000164970, 10 isoforms), 200 replicates of isoform ground truths are simulated using a zero-truncated Gaussian distribution, and then scaled to add up to $10^6$. At least one isoform is ensured to have a ground truth >0. Polyester and miniSim simulators are used to generate 100 pairs of 150 bp short reads and 100 long reads for each replicate of ground truth. The same procedure is followed for *SPINDOC* (ENSG00000168005, three isoforms). In Fig. 3a, for gene *FAM219A* (ENSG00000164970, 10 isoforms), five isoforms (ENST00000379080.5, ENST00000379081.5, ENST00000379084.5, ENST00000379087.5, and ENST00000379089.5) are assumed to be the truly expressed isoforms with high expression level. The ground truths for these selected isoforms are simulated using a zero-truncated Gaussian distribution, while the ground truths for the remaining five isoforms are simulated using a uniform distribution ranging between 0 and 1. Then, the expression levels of truly expressed isoforms are scaled so that the TPMs of all the 10 isoforms sum up to $10^6$. This process is repeated to generate 200 sets of ground truth, and for each set, 100 pairs of 150 bp short reads and 100 long reads are simulated using polyester and miniSim.

In Fig. 5g, to evaluate the uncertainty of isoform usage ratio evaluation, 100 bootstrap samples of reads mapped only to the example genes *PEMT* (ENSG00000133027.18), *MAT2B* (ENSG00000038274.17), *RPL39L* (ENSG00000163923.10), and *TERF1* (ENSG00000147601.15) are generated at each of the four expression levels for each of

the three samples (ESC, PE and PGC). Based on the quantification using miniQuant-L with annotations derived using IsoQuant from cDNA-ONT, the four expression levels are chosen as the quantification results of the example genes, and the 25$^{th}$, 50$^{th}$, and 75$^{th}$ percentiles of TPMs among all the genes in the annotation in ESC cDNA-ONT data. The sequencing depth is the same as the ESC cDNA-ONT data. The expression level for each example gene is the same across the three samples (ESC, PE and PGC) in the bootstrap samples using quantification results. And in the bootstrap samples for the 25$^{th}$, 50$^{th}$, and 75$^{th}$ percentiles, the expression levels for the example genes are all the same. FLAMES and TALON are excluded from the analysis, since FLAMES does not return the quantification result for *TERF1* in the bootstrap samples for the 25$^{th}$, 50$^{th}$, and 75$^{th}$ percentiles, and TALON forces the identification of novel genes and isoforms from the reads. The R package "ggtern" is used for visualization.

**Supplementary Notes 14: Isoforms TPM truncation in evaluation**

In this note, the reason for truncating isoforms TPM at 0.01 in evaluation is introduced. The competing methods exhibit diverse output precisions. For instance, the long read-based methods TALON, FLAIR, and LIQA provide quantification outputs in counts, which lead to a relatively lower precision when subsequently transformed into TPM. However, evaluation metrics such as ARD (absolute relative difference), MARD, and RE are exceptionally sensitive to tiny quantification errors when the true relative abundance is close to 0. For example, for an isoform with a true TPM of 0.0001, quantification tools with precision limited to two decimals may yield results such as 0 or at least 0.01 TPM, resulting in an ARD close to 1. To ensure comparability across all methods, only gene isoforms with true abundances of TPM≥0.01 are retained in the ground truths.

## References


1 Li, W. V. & Li, J. J. Modeling and analysis of RNA-seq data: a review from a statistical perspective. *Quant Biol* **6**, 195-209 (2018). https://doi.org/10.1007/s40484-018-0144-7

2 Au, K. F. *et al.* Characterization of the human ESC transcriptome by hybrid sequencing. *Proc Natl Acad Sci U S A* **110**, E4821-4830 (2013). https://doi.org/10.1073/pnas.1320101110

3 Jiang, H. & Wong, W. H. Statistical inferences for isoform expression in RNA-Seq. *Bioinformatics* **25**, 1026-1032 (2009). https://doi.org/10.1093/bioinformatics/btp113

4 Salzman, J., Jiang, H. & Wong, W. H. Statistical Modeling of RNA-Seq Data. *Stat Sci* **26** (2011). https://doi.org/10.1214/10-STS343

5 Love, M. I., Hogenesch, J. B. & Irizarry, R. A. Modeling of RNA-seq fragment sequence bias reduces systematic errors in transcript abundance estimation. *Nat Biotechnol* **34**, 1287-1291 (2016). https://doi.org/10.1038/nbt.3682

6 Li, J. J., Jiang, C. R., Brown, J. B., Huang, H. & Bickel, P. J. Sparse linear modeling of next-generation mRNA sequencing (RNA-Seq) data for isoform discovery and abundance estimation. *Proc Natl Acad Sci U S A* **108**, 19867-19872 (2011). https://doi.org/10.1073/pnas.1113972108

7 Li, W., Feng, J. X. & Jiang, T. IsoLasso: A LASSO Regression Approach to RNA-Seq Based Transcriptome Assembly. *J Comput Biol* **18**, 1693-1707 (2011). https://doi.org/10.1089/cmb.2011.0171

8 Canzar, S., Andreotti, S., Weese, D., Reinert, K. & Klau, G. W. CIDANE: comprehensive isoform discovery and abundance estimation. *Genome Biol* **17**, 16 (2016). https://doi.org/10.1186/s13059-015-0865-0

9 Bohnert, R. & Ratsch, G. rQuant.web: a tool for RNA-Seq-based transcript quantitation. *Nucleic Acids Res* **38**, W348-351 (2010). https://doi.org/10.1093/nar/gkq448

10 Pachter, L. *Models for transcript quantification from RNA-Seq* (arXiv preprint 1104.3889, 2011).

11 Li, B. & Dewey, C. N. RSEM: accurate transcript quantification from RNA-Seq data with or without a reference genome. *BMC Bioinformatics* **12**, 323 (2011). https://doi.org/10.1186/1471-2105-12-323

12 Patro, R., Duggal, G., Love, M. I., Irizarry, R. A. & Kingsford, C. Salmon provides fast and bias-aware quantification of transcript expression. *Nat Methods* **14**, 417-419 (2017). https://doi.org/10.1038/nmeth.4197

13 Bray, N. L., Pimentel, H., Melsted, P. & Pachter, L. Near-optimal probabilistic RNA-seq quantification. *Nat Biotechnol* **34**, 525-527 (2016). https://doi.org/10.1038/nbt.3519

14 Fahrmeir, L. & Kaufmann, H. Consistency and Asymptotic Normality of the Maximum-Likelihood Estimator in Generalized Linear-Models. *Ann Stat* **13**, 342-368 (1985). https://doi.org/DOI 10.1214/aos/1176346597

15 Higham, N. J. *Accuracy and Stability of Numerical Algorithms*. (Society for Industrial and Applied Mathematics, 2002).

16 Wei, M. Perturbation of the least squares problem. *Linear Algebra and its*

*Applications* **141**, 177-182 (1990). https://doi.org/https://doi.org/10.1016/0024-3795(90)90316-5
17 Francisco, J. P.-P. *et al.* SQANTI3: curation of long-read transcriptomes for accurate identification of known and novel isoforms. *bioRxiv*, 2023.2005.2017.541248 (2023). https://doi.org/10.1101/2023.05.17.541248
18 Weirather, J. L. *et al.* Comprehensive comparison of Pacific Biosciences and Oxford Nanopore Technologies and their applications to transcriptome analysis. *F1000Res* **6**, 100 (2017). https://doi.org/10.12688/f1000research.10571.2
19 Tardaguila, M. *et al.* SQANTI: extensive characterization of long-read transcript sequences for quality control in full-length transcriptome identification and quantification. *Genome Res* **28**, 396-411 (2018). https://doi.org/10.1101/gr.222976.117
