# Supplementary material for: Improving gene isoform quantification with miniQuant
Source: Nat Biotechnol. 2025 Jun 3;44(3):477–89. doi: 10.1038/s41587-025-02633-9 (PMC12346831; doi:10.1038/s41587-025-02633-9)
Supplement: Supplementary file 2 — Reporting Summary [file 41587_2025_2633_MOESM2_ESM.pdf]

nature portfolio

Corresponding author(s): Kin Fai Au

Last updated by author(s): 2/21/2025

Reporting Summary

Nature Portfolio wishes to improve the reproducibility of the work that we publish. This form provides structure for consistency and transparency in reporting. For further information on Nature Portfolio policies, see our Editorial Policies and the Editorial Policy Checklist.

Statistics

For all statistical analyses, confirm that the following items are present in the figure legend, table legend, main text, or Methods section.

n/a	Confirmed	
☐	☒	The exact sample size (*n*) for each experimental group/condition, given as a discrete number and unit of measurement
☐	☒	A statement on whether measurements were taken from distinct samples or whether the same sample was measured repeatedly
☐	☒	The statistical test(s) used AND whether they are one- or two-sided<br>*Only common tests should be described solely by name; describe more complex techniques in the Methods section.*
☒	☐	A description of all covariates tested
☒	☐	A description of any assumptions or corrections, such as tests of normality and adjustment for multiple comparisons
☐	☒	A full description of the statistical parameters including central tendency (e.g. means) or other basic estimates (e.g. regression coefficient) AND variation (e.g. standard deviation) or associated estimates of uncertainty (e.g. confidence intervals)
☐	☒	For null hypothesis testing, the test statistic (e.g. *F*, *t*, *r*) with confidence intervals, effect sizes, degrees of freedom and *P* value noted<br>*Give P values as exact values whenever suitable.*
☒	☐	For Bayesian analysis, information on the choice of priors and Markov chain Monte Carlo settings
☒	☐	For hierarchical and complex designs, identification of the appropriate level for tests and full reporting of outcomes
☒	☐	Estimates of effect sizes (e.g. Cohen's *d*, Pearson's *r*), indicating how they were calculated

Our web collection on statistics for biologists contains articles on many of the points above.

Software and code

Policy information about availability of computer code

Data collection	The cDNA-ONT sequencing is conducted using ONT MiniON/GridION flow cells with R9.4.1 chemistry, with real-time base calling performed by the Guppy (v4.2.3). Illumina RNA-seq sequencing adapters are trimmed by Cutadapt (v1.8.1) with the parameter "-q 20,20 -m 20 --max-n 0.01 --trim-n". Details on software used for sequencing and basecalling has been described in Online Methods.
Data analysis	Datasets are aligned to the hg38 reference genome using minimap2 (v2.24-r1122) for long reads and Bowtie2 (v2.4.1) or Hisat2 (v2.1.0) for short reads. The parameters used for minimap2, Bowtie2 and Hisat2 are shown in Supplementary Table 12.<br>MiniQuant (v1.0) is compared with five short read-based quantification tools (kallisto (v0.46.1), Salmon (v1.4.0), RSEM (v1.3.3), Cufflinks (v2.2.1), StringTie (v2.2.1)), seven long read-based quantification tools (IsoQuant (v3.3.0), Bambu (v3.2.4), FLAIR (v1.5.0), FLAMES (download in April 2022), TALON (v5.0), StringTie2 (v2.2.1), LIQA (v1.1.22)) and the mix mode of StringTie (v2.2.1) that integrates short and long reads to evaluate its effectiveness. See Supplementary Table 12 for details about the application of existing methods.<br>XGBoost (v2.0.3) is used for training gradient boosting model to determine the community-specific weight.<br>For differential expression analysis, the fold changes of gene isoform are calculated using edgeR(v3.40.2).<br>For analysis of isoform switching, identification of gene isoforms is conducted by IsoQuant (v3.3.0) and gene Ontology (GO) analysis is carried out using DAVID (version: 2021).

For manuscripts utilizing custom algorithms or software that are central to the research but not yet described in published literature, software must be made available to editors and reviewers. We strongly encourage code deposition in a community repository (e.g. GitHub). See the Nature Portfolio guidelines for submitting code & software for further information.

Data

Policy information about availability of data

All manuscripts must include a data availability statement. This statement should provide the following information, where applicable:

- Accession codes, unique identifiers, or web links for publicly available datasets
- A description of any restrictions on data availability
- For clinical datasets or third party data, please ensure that the statement adheres to our policy

The long-read (cDNA-ONT, dRNA-ONT) and short-read (Illumina) RNA-seq data generated in this study are available at the NCBI Gene Expression Omnibus (GEO) under the accession number GSE265988. Details of data generation is described in the Online Methods and Data Availability Statement.

Human research participants

Policy information about studies involving human research participants and Sex and Gender in Research.

Reporting on sex and gender	Not applicable since there is no human participants involved in this study and the study are not categorized by sex or gender.
Population characteristics	Not applicable since there is no human participants involved in this study and the study are not categorized by human populations.
Recruitment	Not applicable since there is no human participants involved in this study.
Ethics oversight	Not applicable since there is no human participants involved in this study.

Note that full information on the approval of the study protocol must also be provided in the manuscript.

Field-specific reporting

Please select the one below that is the best fit for your research. If you are not sure, read the appropriate sections before making your selection.

☒ Life sciences ☐ Behavioural & social sciences ☐ Ecological, evolutionary & environmental sciences

For a reference copy of the document with all sections, see nature.com/documents/nr-reporting-summary-flat.pdf

Life sciences study design

All studies must disclose on these points even when the disclosure is negative.

Sample size	Illumina RNA-seq data is generated with three replicates for each of cell lines and long-read RNA-seq data is generated with one replicate for each of cell line. This is a minimum standard in RNA-seq field.
Data exclusions	No dataset by Illumina RNA-seq or long-read RNA-seq technology is excluded in this study.
Replication	Illumina RNA-seq data were generated with three replicates for each of cell lines. All replications are successful.
Randomization	The randomization is irrelevant because the gene isoform quantification is conducted transcriptome-wide without bias.
Blinding	The developers of the miniQuant software is blinded to the validation dataset while running the software for gene isoform quantification.

Reporting for specific materials, systems and methods

We require information from authors about some types of materials, experimental systems and methods used in many studies. Here, indicate whether each material, system or method listed is relevant to your study. If you are not sure if a list item applies to your research, read the appropriate section before selecting a response.

Materials & experimental systems

n/a	Involved in the study
☒	☐ Antibodies
☐	☒ Eukaryotic cell lines
☒	☐ Palaeontology and archaeology
☒	☐ Animals and other organisms
☒	☐ Clinical data
☒	☐ Dual use research of concern

Methods

n/a	Involved in the study
☒	☐ ChIP-seq
☒	☐ Flow cytometry
☒	☐ MRI-based neuroimaging

Eukaryotic cell lines

Policy information about cell lines and Sex and Gender in Research

Cell line source(s)	H1-hESC (WA01 from WiCell)
Authentication	Short tandem repeat authentication was performed.
Mycoplasma contamination	No mycoplasma contamination was detected.
Commonly misidentified lines (See ICLAC register)	None of the cell lines are included in the ver13 update on commonly misidentified cell lines.
