# Supplementary figures and images for: Improving gene isoform quantification with miniQuant
Source: Nat Biotechnol. 2025 Jun 3;44(3):477–89. doi: 10.1038/s41587-025-02633-9 (PMC12346831; doi:10.1038/s41587-025-02633-9)

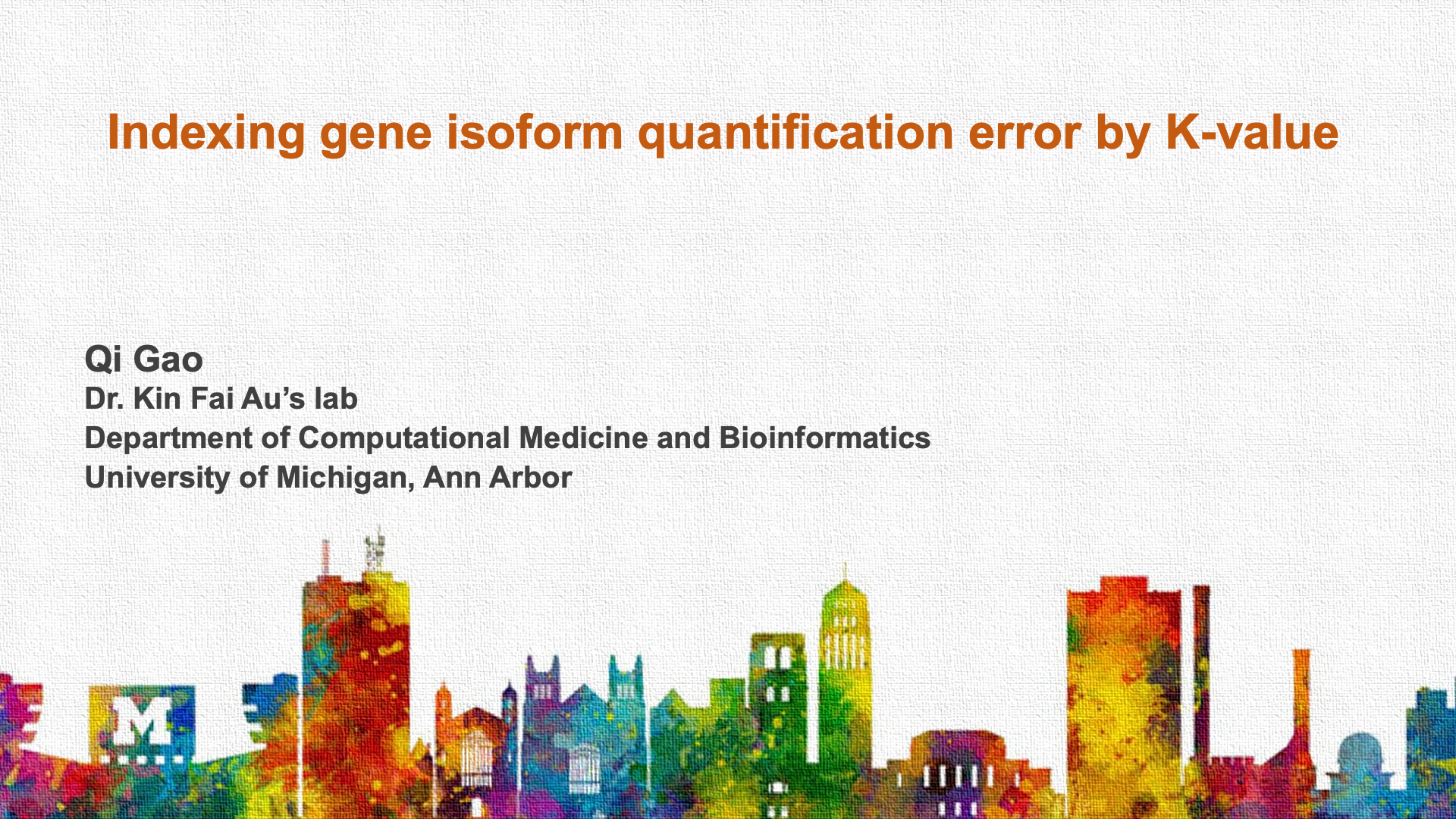

Supplement: Supplementary file 4 — Supplementary code to reproduce the results. The folder ‘Figure_visualization’ stores the code to reproduce the figures. The folder ‘MetricCalculation’ stores the code to calculate the evaluation metrics used in the results. The folder ‘miniQuant’ stores the software of miniQuant to reproduce the quantification results. [file 41587_2025_2633_MOESM4_ESM.zip › Supplementary_code/miniQuant/kvalue_intro_Qi1.png]
